# Diagonalization of the finite Hilbert transform on two adjacent intervals: the Riemann–Hilbert approach

**DOI:** 10.1007/s13324-020-00371-6

**Published:** 2020-06-10

**Authors:** Marco Bertola, Elliot Blackstone, Alexander Katsevich, Alexander Tovbis

**Affiliations:** 1 Department of Mathematics and Statistics, Concordia University, 1455 de Maisonneuve W., Montréal, Québec H3G 1M8 Canada; 2 SISSA, International School for Advanced Studies, Via Bonomea 265, Trieste, Italy; 3 Department of Mathematics, KTH Royal Institute of Technology, Lindstedtsvägen 25, 114 28 Stockholm, Sweden; 4 Department of Mathematics, University of Central Florida, P.O. Box 161364, 4000 Central Florida Blvd, Orlando, FL 32816-1364 USA

**Keywords:** Diagonalization of integral operators with continuous spectrum, Finite Hilbert transforms on multi intervals, Spectral theory of finite Hilbert transforms, Riemann–Hilbert problem, Asymptotics of hypergeometric functions

## Abstract

In this paper we study the spectra of bounded self-adjoint linear operators that are related to finite Hilbert transforms $\mathcal {H}_L:L^2([b_L,0])\rightarrow L^2([0,b_R])$ and $\mathcal {H}_R:L^2([0,b_R])\rightarrow L^2([b_L,0])$. These operators arise when one studies the interior problem of tomography. The diagonalization of $\mathcal {H}_R,\mathcal {H}_L$ has been previously obtained, but only asymptotically when $b_L\ne -b_R$. We implement a novel approach based on the method of matrix Riemann–Hilbert problems (RHP) which diagonalizes $\mathcal {H}_R,\mathcal {H}_L$ explicitly. We also find the asymptotics of the solution to a related RHP and obtain error estimates.

## Introduction

Let $I_{{1}},I_{{2}}\subset \mathbb {R}$ be multiintervals, i.e. the unions of finitely many non-overlapping intervals (but the sets $I_{{1}}, I_{{2}}$ can overlap). The intervals can be bounded or unbounded. Consider the Finite Hilbert transform (FHT) and its adjoint:

1 $$\begin{aligned} \mathcal {H}:\,L^2(I_{{1}})\rightarrow L^2(I_{{2}})\,\ (\mathcal {H}f)(y)= & {} \frac{1}{\pi }\int _{I_{{1}}}\frac{f(x)}{x-y}dx;\ \mathcal {H}^*:\, L^2(I_{{2}})\rightarrow L^2(I_{{1}}),\nonumber \\ (\mathcal {H}^* g)(x)= & {} \frac{1}{\pi }\int _{I_{{2}}}\frac{g(y)}{x-y}dy. \end{aligned}$$

The general problem we consider is to study the spectral properties of $\mathcal {H}$ (and the associated self-adjoint operators $\mathcal {H}^*\mathcal {H}$, $\mathcal {H}\mathcal {H}^*$) depending on the geometry of the sets $I_{{1}},I_{{2}}$. The properties we are interested in include finding the spectrum, establishing the nature of the spectrum (e.g., discrete vs. continuous), and finding the associated resolution of the identity. In the case when $I_{{1}}=I_{{2}}$, these problems were studied starting in the 50s and 60s, see e.g. [20–22, 27–29, 33].

More recently, the problem of the diagonalization of $\mathcal {H}^*\mathcal {H}$ and $\mathcal {H}\mathcal {H}^*$ occurred when solving the problem of image reconstruction from incomplete tomographic data, e.g. when solving the interior problem of tomography [4, 8, 23, 34–36]. In these applications, a significant diversity of different arrangements of $I_{{1}}$, $I_{{2}}$ was encountered: the two sets can be disjoint (i.e., $\text {dist}(I_{{1}}, I_{{2}})>0$), touch each other (i.e., have a common endpoint), or overlap over an interval. Each of these arrangements leads to different spectral properties of the associated FHT. Generally, if $\text {dist}(I_{{1}}, I_{{2}})>0$, the operators $\mathcal {H}^*\mathcal {H}$ and $\mathcal {H}\mathcal {H}^*$ are Hilbert–Schmidt with discrete spectrum [6, 17]. In one particular case of overlap, when $I_{{1}}=[a_1,a_3]$, $I_{{2}}=[a_2,a_4]$, $a_1<a_2<a_3<a_4$, the spectrum is discrete, but has two accumulation points: $\lambda =0$ and $\lambda =1$ [3, 4], where $\lambda$ denotes the spectral parameter. In two cases when $I_{{1}}$, $I_{{2}}$ touch each other, the spectrum is purely absolutely continuous. The case $I_{{1}}=[a_1,a_2]$, $I_{{2}}=[a_2,a_3]$, $a_1<a_2<a_3$, was considered in [19]. The case when $I_{{1}}$, $I_{{2}}$ are unions of more than one sub-interval each and $I_{{1}}\cup I_{{2}}=\mathbb {R}$ was considered in [7].

To obtain the above mentioned results, three methods have been employed. In very few exceptional cases, e.g. when $I_{{1}}\cup I_{{2}}=\mathbb {R}$, it is possible to diagonalize the FHT explicitly via some ingenious transformations. When $I_{{1}}$, $I_{{2}}$ each consist of a single interval (the intervals can be separated, touch, or overlap), the method of a commuting differential operator is used [3, 4, 17–19]. Here the associated singular functions (and kernels of the unitary transformations) are obtained as solutions of the special Sturm-Liouville problems for the differential operator that commutes with the FHT. As is seen, both of these approaches are fairly limited.

In [6], a new powerful approach based on the method of the Riemann–Hilbert Problem (RHP) and the nonlinear steepest descent method of Deift–Zhou was proposed. This approach allows one to treat fairly general cases of $I_{{1}}$, $I_{{2}}$. However, the limitation of this approach so far was the assumption $\text {dist}(I_{{1}}, I_{{2}})>0$, which ensured that the associated operators $\mathcal {H}^*\mathcal {H}$ and $\mathcal {H}\mathcal {H}^*$ are Hilbert–Schmidt. In the case when $I_{{1}}$, $I_{{2}}$ touch each other, the RHP approach encountered several challenges that have not been studied before. Here are the three main ones:

1. In the case of a purely discrete spectrum, residues are used to compute singular functions in [6]. However, in the limit $\text {dist}(I_{{1}}, I_{{2}})\rightarrow 0$ the spectra of the operators change from discrete to continuous. Thus, a different technique is needed to extract the spectral properties of the FHT from the solution of the corresponding RHP;
2. Construction of small $\lambda$ approximations of the RHP solution near the common endpoints (parametrices) was not known, and it had to be developed;
3. The proper boundary conditions near the common endpoints in the formulation of the RHP was not well understood.

As stated earlier, in the particular case when $I_{{1}}=[a_1,a_2]$, $I_{{2}}=[a_2,a_3]$, $a_1<a_2<a_3$, the spectral analysis of the FHT was performed in [19]. Since the method in [19] is based on a commuting differential operator, this method cannot be extended to more general situations of touching intervals, for example, to the case when $I_{{2}}$ consists of two disjoint intervals. The goal of this paper is to extend the RHP and Deift–Zhou approach from [6] to the case when $I_{{1}}$, $I_{{2}}$ touch each other. This problem in its most general setting is very complicated. For example, if $I_{{1}}$, $I_{{2}}$ touch each other at several points, the continuous spectrum may have multiplicity greater than 1 (see [7]). If there are also pieces of $I_{{1}}$ that are at a positive distance from $I_{{2}}$ (or, vice versa), then there will be discrete spectrum accumulating at $\lambda =0$ embedded in the continuous spectrum. In this paper we build the foundation for using the RHP/Deift–Zhou method by studying the case $I_{{1}}=[a_1,a_2]$, $I_{{2}}=[a_2,a_3]$, $a_1<a_2<a_3$, as a model example. Our results include:

1. Formulating the corresponding RHP (including the proper boundary conditions) and explicitly calculating its solution $\varGamma (z;\lambda )$ in terms of hypergeometric functions;
2. Complete spectral analysis of the operators $\mathcal {H}^*\mathcal {H}$ and $\mathcal {H}\mathcal {H}^*$ as well as diagonalization of the operators $\mathcal {H}$, $\mathcal {H}^*$;
3. Calculating the leading order asymptotics of $\varGamma (z;\lambda )$ in the limit $\lambda \rightarrow 0$ in various regions of the complex *z* plane. These regions include, in particular, a small annulus centered at the common endpoint $a_2$.
4. Finally, we also show that the spectral asymptotics of [19] match the explicit spectral results of this paper.

The asymptotics in the annulus around $a_2$ from Item 3 allows us to use $\varGamma (z;\lambda )$ as a parametrix near any common endpoint for more general multiintervals $I_{{1}}$, $I_{{2}}$. This parametrix is the key missing link that is needed to construct the leading order asymptotics of $\varGamma (z;\lambda )$ for the general $I_{{1}}$, $I_{{2}}$. This will be the subject of future research.

The paper is organized as follows. In Sect. 2 we introduce the integral operator

$$\begin{aligned} \hat{K}:L^2([b_L,b_R])\rightarrow L^2([b_L,b_R]),\ b_L<0<b_R, \end{aligned}$$

which, when restricted to $L^2([b_L,0])$ and $L^2([0,b_R])$, coincides with $\frac{1}{2i}\mathcal {H}_L$ and $\frac{1}{2i}\mathcal {H}_R$, respectively (see equation (3)). Here $\mathcal {H}_L$ is the FHT from $L^2([b_L,0])\rightarrow L^2([0,b_R])$, and $\mathcal {H}_R$ is the FHT from $L^2([0,b_R])\rightarrow L^2([b_L,0])$. In the spirit of the above notation, we have $a_1=b_L$, $a_2=0$, and $a_3=b_R$. It can easily be shown that the knowledge of the spectrum of $\hat{K}^2$ allows one to find the spectra of $\mathcal {H}_L^*\mathcal {H}_L$ and $\mathcal {H}_R\mathcal {H}_R^*$. Similarly to the case of disjoint intervals studied in [5], the key observation is that the kernel *K* of $\hat{K}$, see (4), is a kernel of integrable type in the sense of [16]. Therefore, the kernel of the resolvent $\hat{R}$ of $\hat{K}$ is readily available in terms of the solution $\varGamma (z;\lambda )$ of the matrix RHP 1.

Thus, RHP 1 plays a fundamental role in our paper. Because the jump matrix of this RHP is constant in *z*, its solution $\varGamma (z;\lambda )$ satisfies a Fuchsian system of linear differential equations with three singular points and, therefore, can be expressed in terms of hypergeometric functions. An explicit expression for $\varGamma (z;\lambda )$ is obtained in Theorem 1. Using this expression, we show that the matrix $\varGamma (z;\lambda )$ is analytic for $\lambda \in \overline{\mathbb {C}}{\setminus }[-1/2,1/2]$ and has analytic continuations across $(-1/2,0)$ and (0, 1/2) from above and from below (Proposition 2).

In Sect. 3 we explicitly find the unitary operators $U_L: L^2([b_L,0]) \rightarrow L^2([0,1],\sigma _{\chi _L})$ and ${U_R: L^2([0,b_R])\rightarrow L^2([0,1],\sigma _{\chi _R})}$, which diagonalize the operators $\mathcal {H}^*_L\mathcal {H}_L$ and $\mathcal {H}^*_R\mathcal {H}_R$, respectively, see Theorem 8. Here $\sigma _{\chi _L}$, $\sigma _{\chi _R}$ denote the corresponding spectral measures. We prove in Theorems 6 and 7 that the spectrum of the operators $\mathcal {H}^*_L\mathcal {H}_L$ and $\mathcal {H}^*_R\mathcal {H}_R$ is simple, purely absolutely continuous, and consists of the interval [0, 1]. Our approach is based on the resolution of the identity Theorem 3 (see, for example, [2]) and explicit calculation of the jump of the kernel of the resolvent $\hat{R}$ over the spectral set in terms of the hypergeometric functions (Theorem 5). In the process, we find that $\varGamma (z;\lambda )$ satisfies the jump condition in the spectral variable $\lambda$ over the segment $[-\frac{1}{2},\frac{1}{2}]$, see Theorem 4, which, in some sense, is dual to the jump condition in the *z* variable, see RHP 1. This observation is a RHP analogue of certain bispectral problems [15].

The spectrum and diagonalization of the operators $\mathcal {H}_L, \mathcal {H}_R$ was studied in [19], where the authors used a second order differential operator *L*, see (90), which commutes with $\mathcal {H}_L$ and $\mathcal {H}_R$. The Titchmarsh-Weyl theory was utilized in [19] to obtain the small-$\lambda$ asymptotics of the unitary operators $U_1,U_2$ that diagonalize $\mathcal {H}_L$, $\mathcal {H}_R$, i.e. $U_2\mathcal {H}_L U_1^*$ and $U_1\mathcal {H}_R U_2^*$ are multiplication operators. In Sect. 4, we construct the operators $U_1,U_2$, see (115), (116), explicitly in terms of the hypergeometric functions everywhere on the continuous spectrum. We also show, see Theorem 13, that the diagonalization of $\mathcal {H}^*_L\mathcal {H}_L$ and $\mathcal {H}^*_R\mathcal {H}_R$, obtained in Sect. 3, is equivalent to the diagonalization obtained through the operators $U_1,U_2$.

In Sect. 5 we obtain the leading order behavior of $\varGamma (z;\lambda )$ as $\lambda \rightarrow 0$ in different regions of the complex *z*-plane, see Theorem 17. The main tool we use here is the Deift–Zhou nonlinear steepest descent method combined with the *g*-function mechanism, which reduces (asymptotically) the original RHP 1 for $\varGamma (z;\lambda )$ to the so called model RHP 4 for $\varPsi (z)$. The latter represents the leading order approximation of $\varGamma (z;\lambda )$ on compact subsets of $\mathbb {C}{\setminus } [-\frac{1}{2},\frac{1}{2}]$. Since the jump matrices in RHP 4 commute with each other, the model RHP has a simple algebraic solution (Theorem 16). However, due to a singularity at the common endpoint $z=0$, this solution is not unique. In order to select the appropriate $\varPsi (z)$, we need to match it with the leading order behavior of $\varGamma (z;\lambda )$ as $\lambda \rightarrow 0$ in a small annulus centered at $z=0$, which is derived in Theorem 15. This, in turn, requires calculating the leading order approximation of the hypergeometric function $F(a,b;c;\eta )$, where the parameters *a*, *b*, *c* go to infinity in a certain way as $\lambda \rightarrow 0$. Moreover, this approximation must be uniform in a certain large radius annulus $\varOmega$ in the complex $\eta$-plane. Such asymptotics was recently obtained in [26] based on the saddle point method for complex integrals of the type of (133), but error estimates and uniformity needed for our purposes were not addressed there. Thus, we state and prove Theorem 14 for such complex integrals.

Details of the construction of $\varGamma (z;\lambda )$, the proof of Theorem 14, and other auxiliary material can be found in the Appendix.

## Integral operator $\hat{K}$ and RHP

Let us begin by defining the finite Hilbert transforms $\mathcal {H}_L:L^2([b_L,0])\rightarrow L^2([0,b_R])$ and $\mathcal {H}_R:L^2([0,b_R])\rightarrow L^2([b_L,0])$ by

2 $$\begin{aligned} \mathcal {H}_L[f](y):=\frac{1}{\pi }\int _{b_L}^0\frac{f(x)}{x-y}dx,~~ \mathcal {H}_R[g](x):=\frac{1}{\pi }\int _{0}^{b_R}\frac{g(y)}{y-x}dy. \end{aligned}$$

Notice that the adjoint of $\mathcal {H}_L$ is $-\mathcal {H}_R$.

### Definition and properties of $\hat{K}$

We define the integral operator $\hat{K}:L^2([b_L,b_R])\rightarrow L^2([b_L,b_R])$ by the requirements

3 $$\begin{aligned} \hat{K}\big |_{L^2([b_L,0])}=\frac{1}{2i}\mathcal {H}_L, ~~~ \hat{K}\big |_{L^2([0,b_R])}=\frac{1}{2i}\mathcal {H}_R. \end{aligned}$$

Explicitly,

4 $$\begin{aligned} \hat{K}[f](z):=\int _{b_L}^{b_R} K(z,x)f(x)~dx, ~~ \text { where } ~~ K(z,x):=\frac{\chi _L(x)\chi _R(z)+\chi _R(x)\chi _L(z)}{2\pi i(x-z)} \end{aligned}$$

and $\chi _{L},\chi _R$ are indicator functions on $[b_L,0],[0,b_R]$, respectively.

#### Proposition 1

The integral operator $\hat{K}:L^2([b_L,b_R])\rightarrow L^2([b_L,b_R])$ is self-adjoint and bounded.

#### Proof

The boundedness of $\hat{K}$ follows from the boundedness of the Hilbert transform on $L^2(\mathbb {R})$ and we can see that $\hat{K}$ is self-adjoint because $K(z,x)=\overline{K(x,z)}$. $\square$

### Resolvent of $\hat{K}$ and the Riemann–Hilbert problem

The operator $\hat{K}$ falls within the class of “integrable kernels” (see [16]) and it is known that its spectral properties are intimately related to a suitable Riemann–Hilbert problem. In particular, the kernel of the resolvent integral operator $\hat{R}=\hat{R}(\lambda ):L^2([b_L,b_R])\rightarrow L^2([b_L,b_R])$, defined by

5 $$\begin{aligned} (I+\hat{R}(\lambda ))\left( I-\frac{1}{\lambda }\hat{K}\right) =I, \end{aligned}$$

can be expressed through the solution $\varGamma (z;\lambda )$ of the following RHP.

#### Riemann–Hilbert Problem 1

Find a $2\times 2$ matrix-function $\varGamma (z;\lambda )$, $\lambda \in \overline{\mathbb {C}}{\setminus }[-1/2,1/2]$, analytic for $z\in \overline{\mathbb {C}}{\setminus } [b_L,b_R]$ and satisfying

6 $$\begin{aligned} \varGamma (z_+;\lambda )&=\varGamma (z_-;\lambda )\begin{bmatrix} 1 &{}\quad -\frac{i}{\lambda } \\ 0 &{} \quad 1 \end{bmatrix}, ~~ z\in [b_L,0], \end{aligned}$$

7 $$\begin{aligned} \varGamma (z_+;\lambda )&= \varGamma (z_-;\lambda )\begin{bmatrix} 1 &{} \quad 0 \\ \frac{i}{\lambda } &{} \quad 1 \end{bmatrix}, ~~ z\in [0,b_R], \end{aligned}$$

8 $$\begin{aligned} \varGamma (z;\lambda )&= \begin{bmatrix} {\text {O}}\left( 1\right)&\quad {\text {O}}\left( \log (z-b_L)\right) \end{bmatrix}, ~~ z\rightarrow b_L, \end{aligned}$$

9 $$\begin{aligned} \varGamma (z;\lambda )&= \begin{bmatrix} {\text {O}}\left( \log (z-b_R)\right)&\quad {\text {O}}\left( 1\right) \end{bmatrix}, ~~ z\rightarrow b_R, \end{aligned}$$

10 $$\begin{aligned} \varGamma (z;\lambda )&\in L^2([b_L,b_R]), \end{aligned}$$

11 $$\begin{aligned} \varGamma (z;\lambda )&= \mathbf{1}+ {\text {O}}\left( z^{-1}\right) , ~~ z\rightarrow \infty , \end{aligned}$$

where $\mathbf{1}$ denotes the identity matrix. The endpoint behavior of $\varGamma (z;\lambda )$ is described column-wise, the intervals $(b_L,0)$ and $(0,b_R)$ are positively oriented, and $z_\pm$ denotes the values on the positive/negative side of the jump contour $(b_L,b_R)$ respectively.

As described in Appendix A, we are able to construct the solution of RHP 1 in terms of the hypergeometric functions1213where $h_\infty ,s_\infty$ are linearly independent solutions of the ODE

14 $$\begin{aligned} z(1-z)w''(z)+a(a+1)w(z)=0, \end{aligned}$$

and $$a=a(\lambda )$$, where

15 $$\begin{aligned} a(\lambda ):=\frac{1}{i\pi }\ln \left( \frac{i+\sqrt{4\lambda ^2-1}}{2\lambda }\right) . \end{aligned}$$

The principle branch of the log is taken and $\sqrt{4\lambda ^2-1}=2\lambda +{\text {O}}\left( 1\right)$ as $\lambda \rightarrow \infty$, so it can be shown that $a(\lambda )$ is analytic for $\lambda \in \overline{\mathbb {C}}{\setminus }[-1/2,1/2]$. This function $a(\lambda )$ will occur frequently throughout this paper so we have listed its relevant properties in Appendix B. We will often write *a* in place of $a(\lambda )$ for convenience. Recall that the standard Pauli matrices are

16 $$\begin{aligned} \sigma _1=\begin{bmatrix} 0 &{} \quad 1 \\ 1 &{} \quad 0 \end{bmatrix}, \qquad \sigma _2=\begin{bmatrix} 0 &{} \quad -i \\ i &{} \quad 0 \end{bmatrix}, \qquad \sigma _3=\begin{bmatrix} 1 &{} \quad 0 \\ 0 &{} \quad -1 \end{bmatrix}. \end{aligned}$$

In Appendix A, we describe how to construct the (unique!) solution to RHP 1.

#### Theorem 1

For $\lambda \in \overline{\mathbb {C}}{\setminus }[-1/2,1/2]$, the unique solution to RHP 1 is

17 $$\begin{aligned} \varGamma (z;\lambda )=\sigma _2Q^{-1}(\lambda )\hat{\varGamma }^{-1}\left( M_1(\infty )\right) \begin{bmatrix} 1 &{} \quad \frac{b_Lb_R}{z(b_R-b_L)(a+1)} \\ 0 &{} \quad 1 \end{bmatrix}\hat{\varGamma }\left( M_1(z)\right) Q(\lambda )\sigma _2, \end{aligned}$$

where $$M_1(z)=\frac{b_R(z-b_L)}{z(b_R-b_L)}$$ and

18 $$\begin{aligned}&Q=\begin{bmatrix} -\tan (a\pi ) &{} \quad 0 \\ 0 &{} \quad 4^{2a+1}e^{a\pi i}\frac{\varGamma (a+3/2)\varGamma (a+1/2)}{\varGamma (a)\varGamma (a+2)} \end{bmatrix}\begin{bmatrix} 1 &{} \quad e^{a\pi i} \\ -e^{a\pi i} &{} \quad 1 \end{bmatrix}, \nonumber \\&\hat{\varGamma }(z)=\begin{bmatrix} h_\infty \left( z\right) &{} \quad s_\infty \left( z\right) \\ h_\infty '\left( z\right) &{} \quad s_\infty '\left( z\right) \end{bmatrix}. \end{aligned}$$

Here $a:=a(\lambda )$ which is defined in (15) and $h_\infty ,s_\infty$ are defined in (12),(13).

#### Remark 1

For any $\lambda \in \mathbb {C}{\setminus }[-1/2,1/2]$, the function $\varGamma (z;\lambda )$ has the symmetries

19 $$\begin{aligned} \overline{\varGamma (\overline{z};\overline{\lambda })}=\varGamma (z;\lambda ), \quad \sigma _3\varGamma (z;-\lambda )\sigma _3=\varGamma (z;\lambda ) \end{aligned}$$

which follow from the observation that $\sigma _3\varGamma (z;-\lambda )\sigma _3$ and $\overline{\varGamma (\overline{z};\overline{\lambda })}$ also solve RHP 1.

#### Proposition 2

The matrix $\varGamma (z;\lambda )$, defined in (17), is analytic for $\lambda \in \overline{\mathbb {C}}{\setminus }[-1/2,1/2]$ and can be analytically continued across the intervals $(-1/2,0)$ and (0, 1/2) from above and from below.

#### Proof

Recall from [24] 15.2 that the hypergeometric function *F*(*a*, *b*; *c*; *z*) (here *a*, *b*, *c* are generic parameters) is an entire function of *a*, *b* and meromorphic in *c* with poles at non positive integers. It easy to see that $\varGamma (z;\lambda )$ is analytic for $\lambda \in \overline{\mathbb {C}}{\setminus }[-1/2,1/2]$ because $a(\lambda )$ is analytic and $|\mathfrak {R}[a(\lambda )]|<\frac{1}{2}$ for $\lambda \in \mathbb {C}{\setminus }[-1/2,1/2]$. Note that $a(\lambda )=0$ only for $\lambda =\infty$, so we can see that the second row of $Q(\lambda )$ has a simple zero when $\lambda =\infty$ and the second column of $\hat{\varGamma }(z,\lambda )$ has a simple pole when $\lambda =\infty$. Thus the product $\hat{\varGamma }(z,\lambda )Q(\lambda )={\text {O}}\left( 1\right)$ as $\lambda \rightarrow \infty$ so $\varGamma (z;\lambda )$ is analytic at $\lambda =\infty$ as well. Using Appendix B, we can see that $a(\lambda )\not \in \mathbb {R}$ for $\lambda \in (-1/2,0)\cup (0,1/2)$. Thus, $\varGamma (z;\lambda )$ can be analytically continued across the intervals $(-1/2,0)$ and (0, 1/2) from above and from below (but these continuations do not coincide on $(-1/2,0)$ and on (0, 1/2)). $\square$

We now show the relation between $\hat{K}$ and $\varGamma (z;\lambda )$.

#### Theorem 2

With the resolvent operator $\hat{R}$ defined by (5), let the kernel of $\hat{R}$ be denoted by *R*. Then,

20 $$\begin{aligned} R(z,x;\lambda )= & {} \frac{\mathbf {g}_1^t(x)\varGamma ^{-1}(x;\lambda )\varGamma (z;\lambda )\mathbf {f}_1(z)}{2\pi i\lambda (z-x)} \text { where } \mathbf {f}_1(z)=\begin{bmatrix} i\chi _L(z) \\ \chi _R(z) \end{bmatrix}, \nonumber \\ \mathbf {g}_1(x)= & {} \begin{bmatrix} -i\chi _R(x) \\ \chi _L(x) \end{bmatrix}. \end{aligned}$$

The matrix $\varGamma (z;\lambda )$ is defined in (17) and functions $\chi _L,\chi _R$ are indicator functions on $[b_L,0],[0,b_R]$, respectively.

The proof is the same as in [6] (Lemma 3.16) so it will be omitted here. An important ingredient of the proof is the observation that the jump of $\varGamma (z;\lambda )$ can be compactly written as

21 $$\begin{aligned} \varGamma (z_+;\lambda )=\varGamma (z_-;\lambda )\left( \mathbf{1}-\frac{1}{\lambda }\mathbf {f}_1(z)\mathbf {g}_1^t(z)\right) \end{aligned}$$

for $$z\in [b_L,b_R]$$ and

22 $$\begin{aligned} K(z,x)=\frac{\mathbf {f}_1^t(z)\mathbf {g}_1(x)}{2\pi i(x-z)}, \end{aligned}$$

where *K*(*z*, *x*) is the kernel of $\hat{K}$, see (4).

## Spectral properties and diagonalization $\mathcal {H}_R^*\mathcal {H}_R$ and $\mathcal {H}_L^*\mathcal {H}_L$

The goal of this section is to construct unitary operators ${U_R:L^2([0,b_R])\rightarrow L^2(J,\sigma _R)}$ and $U_L:L^2([0,b_R])\rightarrow L^2(J,\sigma _L)$ such that

23 $$\begin{aligned} U_R\mathcal {H}^*_R\mathcal {H}_RU_R^*=\lambda ^2, ~~ U_L\mathcal {H}^*_L\mathcal {H}_LU_L^*=\lambda ^2 \end{aligned}$$

where $\lambda ^2$ is a multiplication operator (the space is clear by context), $J:=\{\lambda ^2:0\le \lambda ^2\le 1\}$, and the spectral measures $\sigma _L,\sigma _R$ are to be determined. This is to be understood in the sense of operator equality on $L^2(J,\sigma _R),L^2(J,\sigma _L)$, respectively. We will begin this section with a brief summary of the spectral theory for a self-adjoint operator with simple spectrum.

### Basic facts about diagonalizing a self-adjoint operator with simple spectrum

For an in-depth review of the spectral theorem for self-adjoint operators, see [2, 11, 32]. We present a short summary of this topic which is directly related to the needs of this paper. Let $\mathcal {K}$ be a Hilbert space and let *A* be a self-adjoint operator with simple spectrum acting on $\mathcal {K}$. Recall from [2], that a self-adjoint operator has simple spectrum if there is a vector $g\in \mathcal {K}$ so that the span of $\hat{E}_\varDelta [g]$, where $\varDelta$ runs through the set of all subintervals of the real line, is dense in $\mathcal {K}$. Here the family of operators $\hat{E}_t$ denotes the so-called *resolution of the identity* for the operator *A*, which we define in (25). Define $\hat{R}$, the resolvent of *A*, via the formula

24 $$\begin{aligned} \hat{R}(t)=(tI-A)^{-1} \end{aligned}$$

for $t\not \in \mathbb {R}$. Then, according to [11] p.921, the resolution of the identity is computed by the formula

25 $$\begin{aligned} \hat{E}_{(\alpha ,\beta )}:=\hat{E}_\beta -\hat{E}_\alpha =\lim _{\epsilon \rightarrow 0^+}\lim _{\delta \rightarrow 0^+}\int _{\alpha +\delta }^{\beta -\delta }\frac{-1}{2\pi i}\left[ \hat{R}(t+i\epsilon )-\hat{R}(t-i\epsilon )\right] ~dt, \end{aligned}$$

where $\alpha <\beta$. Once we obtain $\hat{E}_t$, we can construct the unitary operators which will diagonalize *A*, as described in the following Theorem from [2] p.279.

#### Theorem 3

If *A* is a self-adjoint operator with simple spectrum, if *g* is any generating element, and if $\sigma (t)=\langle \hat{E}_t[g],g \rangle$, then the formula

26 $$\begin{aligned} f=\int _{\mathbb {R}}\tilde{f}(t)~d\hat{E}_t[g] \end{aligned}$$

associates with each function $\tilde{f}\in L^2(\mathbb {R},\sigma )$ a vector $f\in \mathcal {K}$, and this correspondence is an isometric mapping of $L^2(\mathbb {R},\sigma )$ onto $\mathcal {K}$. It maps the domain *D*(*Q*) of the multiplication operator *Q* in $L^2(\mathbb {R},\sigma )$ into the domain *D*(*A*) of the operator *A*, and if the element $f\in D(A)$ corresponds to the function $\tilde{f}\in L^2(\mathbb {R},\sigma )$, then the element *Af* corresponds to the function $t\tilde{f}(t)$.

#### Remark 2

In short, Theorem 3 says that $\sigma (t):=\langle \hat{E}_t[g],g \rangle$ defines the spectral measure (*g* is any generating element) and the operator $U^*:L^2(\mathbb {R},\sigma )\rightarrow \mathcal {K}$ defined by

27 $$\begin{aligned} U^*[\tilde{f}](x):=\int _\mathbb {R}\tilde{f}(t)~d\hat{E}_t[g](x;t) \end{aligned}$$

is unitary. Moreover,

28 $$\begin{aligned} UAU^*=t \end{aligned}$$

in the sense of operator equality on $L^2(\mathbb {R},\sigma )$.

Thus our immediate goal moving forward is to construct the resolution of the identity for $\mathcal {H}_R^*\mathcal {H}_R$ and $\mathcal {H}^*_L\mathcal {H}_L$.

### Resolution of the identity for $\mathcal {H}_R^*\mathcal {H}_R$ and $\mathcal {H}^*_L\mathcal {H}_L$

From (25), knowledge of the resolvent operator is paramount. We are able to express the resolvents of $\mathcal {H}_R^*\mathcal {H}_R,\mathcal {H}_L^*\mathcal {H}_L$ in terms of the resolvent of $\hat{K}$.

#### Proposition 3

The resolvent of $\mathcal {H}_R^*\mathcal {H}_R$ and $\mathcal {H}_L^*\mathcal {H}_L$ is

29 $$\begin{aligned} \mathcal {R}_R(\lambda ^2)&:=\left( I-\frac{1}{\lambda ^2}\mathcal {H}_R^*\mathcal {H}_R\right) ^{-1}=I+\pi _R\hat{R}(\lambda /2)\pi _R, \end{aligned}$$

30 $$\begin{aligned} \mathcal {R}_L(\lambda ^2)&:=\left( I-\frac{1}{\lambda ^2}\mathcal {H}_L^*\mathcal {H}_L\right) ^{-1}=I+\pi _L\hat{R}(\lambda /2)\pi _L, \end{aligned}$$

where $\pi _R:L^2([b_L,b_R])\rightarrow L^2([0,b_R])$, $\pi _L:L^2([b_L,b_R])\rightarrow L^2([b_L,0])$ are orthogonal projections (i.e. restrictions), $\hat{R}(\lambda )$ is defined by the relation (5) and the kernel of $\hat{R}(\lambda )$ is computed in Theorem 2.

#### Remark 3

The resolvents of $\mathcal {H}_R^*\mathcal {H}_R$ and $\mathcal {H}_L^*\mathcal {H}_L$, defined in (29), (30), slightly differ from the standard definition given by (24). It is easy to see that

31 $$\begin{aligned} \frac{\mathcal {R}_R(\lambda ^2)}{\lambda ^2}=\left( \lambda ^2I-\mathcal {H}^*_R\mathcal {H}_R\right) ^{-1}, \end{aligned}$$

and the same is true for the resolvent of $\mathcal {H}_L^*\mathcal {H}_L$.

#### Proof

In the direct sum decomposition $L^2([b_L,b_R])=L^2([b_L,0])\oplus L^2([0,b_R])$, $\hat{K}$ has the block structure

32 $$\begin{aligned} \hat{K}=\begin{bmatrix} 0 &{} \quad -\frac{i}{2}\mathcal {H}_L \\ -\frac{i}{2}\mathcal {H}_R &{} \quad 0 \end{bmatrix}=\begin{bmatrix} 0 &{}\quad \frac{i}{2}\mathcal {H}_R^* \\ -\frac{i}{2}\mathcal {H}_R &{}\quad 0 \end{bmatrix}=\begin{bmatrix} 0 &{} \quad -\frac{i}{2}\mathcal {H}_L \\ \frac{i}{2}\mathcal {H}_L^* &{}\quad 0 \end{bmatrix}. \end{aligned}$$

For $\lambda$ sufficiently large, we can write (recall that $\hat{K}$ is bounded, see Proposition 1)

33 $$\begin{aligned} I+\hat{R}(\lambda )=\left( I-\frac{1}{\lambda }\hat{K}\right) ^{-1}=\sum _{n=0}^\infty \left( \frac{\hat{K}}{\lambda }\right) ^n \end{aligned}$$

where all the even powers in the right hand side of (33) are block diagonal and all the odd powers in (33) are block off-diagonal. The result is extended to all $\lambda \not \in \mathbb {R}$ via analytic continuation. Similarly, we can write

34 $$\begin{aligned} \left( I-\frac{1}{\lambda ^2}\mathcal {H}_R^*\mathcal {H}_R\right) ^{-1}=\sum _{n=0}^\infty \left( \frac{\mathcal {H}_R^*\mathcal {H}_R}{\lambda ^2}\right) ^n \end{aligned}$$

and comparing with the series in (33) gives our result for the resolvent of $\mathcal {H}_R^*\mathcal {H}_R$. The proof for the resolvent of $\mathcal {H}_L^*\mathcal {H}_L$ is nearly identical. $\square$

To construct the resolution of the identity (see (25)), we need to compute the jump of the resolvent of $\mathcal {H}_R^*\mathcal {H}_R$ and $\mathcal {H}_L^*\mathcal {H}_L$ in the $\lambda$-plane. The kernel of the resolvent is expressed in terms of $\varGamma (z;\lambda )$ (see Theorem 2), so we need to compute the jump of $\varGamma (z;\lambda )$ in the $\lambda$-plane.

#### Remark 4

In the remaining sections of this paper we will frequently encounter the following Möbius transformations:

35 $$\begin{aligned} M_1(x)&=\frac{b_R(x-b_L)}{x(b_R-b_L)}, ~~ M_2(x)=\frac{b_Rb_Lx}{x(b_R+b_L)-b_Rb_L}, \end{aligned}$$

36 $$\begin{aligned} M_3(x)&=M_1(M_2(x))=\frac{-b_L(x-b_R)}{x(b_R-b_L)}, ~~ M_4(x)=\frac{x(b_R-b_L)}{x(b_R+b_L)-2b_Rb_L}. \end{aligned}$$

Define functions

37 $$\begin{aligned} d_R(z;\lambda )&:=\alpha (\lambda )h_\infty '\left( M_1(z)\right) +\beta (\lambda )s_\infty '\left( M_1(z)\right) , \end{aligned}$$

38 $$\begin{aligned} d_L(z;\lambda )&:=-e^{a\pi i}\alpha (\lambda )h_\infty '\left( M_1(z)\right) +e^{-a\pi i}\beta (\lambda )s_\infty '\left( M_1(z)\right) , \end{aligned}$$

where $h_\infty ',s_\infty '$ is defined (12), (13), $a:=a(\lambda )$ is defined in (15), and coefficients $\alpha (\lambda ),\beta (\lambda )$ are

39 $$\begin{aligned} \alpha (\lambda ):=\frac{e^{-a\pi i}\tan (a\pi )\varGamma (a)}{4^{a+1}\varGamma (a+3/2)}, ~~ \beta (\lambda ):=\frac{4^{a}e^{a\pi i}\varGamma (a+1/2)}{\varGamma (a+2)}. \end{aligned}$$

The functions $d_L,d_R$ will play a major role in this section so we have compiled all of their relevant properties in Appendix C. Importantly, it is shown that both $d_L(z;\lambda )$ and $d_R(z;\lambda )$ are single valued when $\lambda \in (-1/2,0)$. For $\lambda \in (-1/2,0)\cup (0,1/2)$, define vectors

40 $$\begin{aligned} \mathbf {f}_2(z,\lambda )&:=\frac{-2b_Rb_L|\lambda |(2a_-(-|\lambda |)+1)}{b_R-b_L}\begin{bmatrix} d_R(z;-|\lambda |) \\ \text {sgn}(\lambda )d_L(z;-|\lambda |) \end{bmatrix}, \nonumber \\ \mathbf {g}_2(z,\lambda )&:=\begin{bmatrix} -\text {sgn}(\lambda )d_L(z;-|\lambda |) \\ d_R(z;-|\lambda |) \end{bmatrix}. \end{aligned}$$

We are now ready to compute the jump of $\varGamma (z;\lambda )$ in the $\lambda -$plane.

#### Theorem 4

For $$\lambda \in (-1/2,0)\cup (0,1/2)$$,

41 $$\begin{aligned} \varGamma (z;\lambda _+)=\varGamma (z;\lambda _-)\left[ \mathbf{1}-\frac{1}{z}\mathbf {f}_2(z,\lambda )\mathbf {g}_2^t(z,\lambda )\right] , \end{aligned}$$

where $\varGamma (z;\lambda )$ is defined in (17).

#### Proof

The proof is straightforward; we have

42 $$\begin{aligned} \varGamma (z;\lambda _+)=\varGamma (z;\lambda _-)\varGamma ^{-1}(z;\lambda _-)\varGamma (z;\lambda _+) \end{aligned}$$

and thus we compute $\varGamma ^{-1}(z;\lambda _-)\varGamma (z;\lambda _+)$ to obtain the stated result. We see from (17) that $\varGamma (z;\lambda )$ is defined in terms of $\hat{\varGamma }, Q$ which are defined in (18). For $\lambda \in (-1/2,0)$ (standard orientation), we find that

43 $$\begin{aligned} \hat{\varGamma }\left( z,\lambda _+\right)&=\hat{\varGamma }\left( z,\lambda _-\right) \sigma _1, \end{aligned}$$

44 $$\begin{aligned} Q_+(\lambda )&=\left[ \frac{-\tan (a\pi )\varGamma (a)\varGamma (a+2)}{e^{2\pi ia}4^{2a+1}\varGamma (a+3/2)\varGamma (a+1/2)}\right] _-\sigma _1Q_-(\lambda ). \end{aligned}$$

Recall from Proposition 7 that $a_+(\lambda )+a_-(\lambda )=-1$ for $\lambda \in (-1/2,0)$ thus the jump for *Q* follows. Then by inspection, we see from (12), (13)

45 $$\begin{aligned} h_\infty (z,a_+(\lambda ))=h_\infty (z,-1-a_-(\lambda ))=s_\infty (z,a_-(\lambda )) \end{aligned}$$

so the jump of $\hat{\varGamma }$ follows. Now using (17), (43), (44) we obtain

46 $$\begin{aligned}&\varGamma ^{-1}(z;\lambda _-)\varGamma (z;\lambda _+)\nonumber \\&=\mathbf{1}-\frac{b_Lb_R}{z(b_R-b_L)}\left( \frac{2a+1}{a(a+1)}\right) _-M^{-1}(z,\lambda _-)\begin{bmatrix} 0 &{} \quad 1 \\ 0 &{}\quad 0 \end{bmatrix}M(z,\lambda _-) \end{aligned}$$

where we have used $\hat{\varGamma }_-(M_1(\infty ))Q_-Q^{-1}_+\hat{\varGamma }_+(M_1(\infty ))=c_-(\lambda )\mathbf{1}$ and $c_-(\lambda )$ is the scalar appearing on the right hand side of (44). Here $M(z,\lambda ):=\hat{\varGamma }\left( M_1(z)\right) Q\sigma _2$. Let $m_{21},m_{22}$ denote the (2,1), (2,2) elements of the matrix *M*, respectively. Then,

$$\begin{aligned} M^{-1}\begin{bmatrix} 0 &{} \quad 1 \\ 0 &{}\quad 0 \end{bmatrix}M&=|M|^{-1}\begin{bmatrix} m_{22}m_{21} &{}\quad m_{22}^2 \\ -m_{21}^2 &{}\quad -m_{21}m_{22} \end{bmatrix}=|M|^{-1}\begin{bmatrix} m_{22} \\ -m_{21} \end{bmatrix}\begin{bmatrix} m_{21}&\quad m_{22} \end{bmatrix} \end{aligned}$$

and, explicitly,

$$\begin{aligned}&m_{21}(z;\lambda )=\frac{ie^{a\pi i}\varGamma (a+3/2)}{4^{-a-1}\varGamma (a)}d_L(z;\lambda ), \qquad m_{22}(z;\lambda )=\frac{ie^{a\pi i}\varGamma (a+3/2)}{4^{-a-1}\varGamma (a)}d_R(z;\lambda ), \\&|M(z;\lambda )|=-\frac{2e^{2\pi ia}4^{2a+1}\varGamma ^2\left( a+\frac{3}{2}\right) }{\lambda a(a+1)\varGamma ^2(a)}. \end{aligned}$$

To compute |*M*| we have used (224). Finally, we calculate

$$\begin{aligned}&\left( \frac{2a+1}{a(a+1)}\right) _-M^{-1}(z,\lambda _-)\begin{bmatrix} 0 &{} \quad 1 \\ 0 &{} \quad 0 \end{bmatrix}M(z,\lambda _-)\\&\quad =\left( \frac{2a+1}{a(a+1)|M|}\right) _-\begin{bmatrix} m_{22}(z;\lambda _-) \\ -m_{21}(z;\lambda _-) \end{bmatrix}\begin{bmatrix} m_{21}(z;\lambda _-)&\quad m_{22}(z;\lambda _-) \end{bmatrix} \\&\quad =2\lambda \left( 2a_-+1\right) \begin{bmatrix} d_R(z;\lambda ) \\ -d_L(z;\lambda ) \end{bmatrix}\begin{bmatrix} d_L(z;\lambda )&\quad d_R(z;\lambda ) \end{bmatrix} \end{aligned}$$

which can be used to obtain the desired result for $\lambda \in (-1/2,0)$.

Now, to calculate the jump of $\varGamma (z;\lambda )$ when $\lambda \in (0,1/2)$, we take advantage of the symmetry $\varGamma (z;\lambda )=\sigma _3\varGamma (z;-\lambda )\sigma _3$, see Remark 1. For brevity, let

47 $$\begin{aligned} J(z;\lambda ):=\mathbf{1}-\frac{1}{z}\mathbf {f}_2(z,\lambda )\mathbf {g}_2^t(z,\lambda ). \end{aligned}$$

Using (41) for $\lambda \in (-1/2,0)$, we obtain

48 $$\begin{aligned} \varGamma (z;\lambda _+)=\varGamma (z;\lambda _-)J(z;\lambda _-) \end{aligned}$$

only for $\lambda \in (-1/2,0)$. For $\lambda \in (0,1/2)$,

49 $$\begin{aligned} \varGamma (z;\lambda _+)&=\sigma _3\varGamma (z,(-\lambda )_-)\sigma _3=\sigma _3\varGamma (z,(-\lambda )_+)J^{-1}(z;(-\lambda )_-)\sigma _3\nonumber \\&=\varGamma (z;\lambda _-)\sigma _3J^{-1}(z;(-\lambda )_-)\sigma _3. \end{aligned}$$

It can now be verified that

50 $$\begin{aligned} \sigma _3J^{-1}(z;(-\lambda )_-)\sigma _3=\mathbf{1}-\frac{1}{z}\mathbf {f}_2(z,\lambda )\mathbf {g}_2^t(z,\lambda _-) \end{aligned}$$

for $$\lambda \in (0,1/2)$$. $$\square $$

Recall from Proposition 3 and Theorem 2 that the resolvents of $\mathcal {H}^*_L\mathcal {H}_L$, $\mathcal {H}^*_R\mathcal {H}_R$ are expressed in terms of $\varGamma (z;\lambda )$. In light of the previous Theorem, we can now compute the jump of the resolvents of $\mathcal {H}^*_L\mathcal {H}_L$, $\mathcal {H}^*_R\mathcal {H}_R$ in the $\lambda$ plane, which is required to construct the resolution of the identity, see (25).

#### Theorem 5

The kernel of $\hat{R}(\lambda )$ is single valued for $\lambda \in \overline{\mathbb {C}}{\setminus }[-1/2,1/2]$ and satisfies the jump property

51 $$\begin{aligned} R(z,x;\lambda _+)-R(z,x;\lambda _-)=\frac{\begin{bmatrix} -i\chi _R(x)&\chi _L(x) \end{bmatrix}\mathbf {f}_2(x,\lambda )\mathbf {g}_2^t(z,\lambda )\begin{bmatrix} i\chi _L(z) \\ \chi _R(z) \end{bmatrix}}{2\pi i\lambda xz} \end{aligned}$$

for $\lambda \in (-1/2,0)\cup (0,1/2)$, where $\chi _L,\chi _R$ are the characteristic functions on $(b_L,0), (0,b_R)$ and $R(z,x;\lambda )$, $\mathbf {f}_2(x,\lambda ),\mathbf {g}_2(z,\lambda )$ are defined in (20), (40), respectively.

#### Proof

Recall, from (2), that

52 $$\begin{aligned} R(z,x;\lambda )=\frac{\mathbf {g}_1^t(x)\varGamma ^{-1}(x;\lambda )\varGamma (z;\lambda )\mathbf {f}_1(z)}{2\pi i\lambda (z-x)}. \end{aligned}$$

From Proposition 2 we can see that $\varGamma (z;\lambda )$ is single valued for $\lambda \in \overline{\mathbb {C}}{\setminus }[-1/2,1/2]$ so from (2) it immediately follows that the kernel of $\hat{R}(\lambda )$ is also single valued for $\lambda \in \overline{\mathbb {C}}{\setminus }[-1/2,1/2]$. Let $\varDelta _\lambda F(\lambda ):=F(\lambda _+)-F(\lambda _-)$ for any *F*. To prove the result we need to compute $\varDelta _\lambda [\varGamma ^{-1}(x;\lambda )\varGamma (z;\lambda )]$. For $\lambda \in (-1/2,0)$, we calculate (see proof of Theorem 4)

53 $$\begin{aligned} \varGamma ^{-1}(x;\lambda _-)\varGamma (z;\lambda _-)&=M^{-1}(x,\lambda _-)\begin{bmatrix} 1 &{} \frac{b_Lb_R(x-z)}{xz(b_R-b_L)(a+1)} \\ 0 &{} 1 \end{bmatrix}_-M(z,\lambda _-). \end{aligned}$$

Using $M(z,\lambda _+)=c_-(\lambda )M(z,\lambda _-)$, where $c_-(\lambda )$ is the scalar found in the right hand side of (44),

54 $$\begin{aligned} \varGamma ^{-1}(x;\lambda _+)\varGamma (z;\lambda _+)&=M^{-1}(x,\lambda _+)\begin{bmatrix} 1 &{} \quad \frac{b_Lb_R(x-z)}{xz(b_R-b_L)(a+1)} \\ 0 &{} \quad 1 \end{bmatrix}_+M(z,\lambda _+) \nonumber \\&=M^{-1}(x,\lambda _-)\begin{bmatrix} 1 &{}\quad \frac{-b_Lb_R(x-z)}{axz(b_R-b_L)} \\ 0 &{} \quad 1 \end{bmatrix}_-M(z,\lambda _-), \end{aligned}$$

so we have that

55 $$\begin{aligned} \varDelta _\lambda [\varGamma ^{-1}(x;\lambda )\varGamma (z;\lambda )]&=\left( \frac{-b_Lb_R(x-z)(2a+1)}{xza(a+1)(b_R-b_L)}\right) _-M^{-1}(x,\lambda _-)\begin{bmatrix} 0 &{} 1 \\ 0 &{} 0 \end{bmatrix}M(z,\lambda _-) \nonumber \\&=\frac{z-x}{xz}\mathbf {f}_2(x,\lambda )\mathbf {g}_2^t(z,\lambda ). \end{aligned}$$

Now for $\lambda \in (0,1/2)$, we again take advantage of the symmetry $\varGamma (z;\lambda )=\sigma _3\varGamma (z;-\lambda )\sigma _3$, see Remark 1. The process is the same as in the proof of Theorem 4. Directly from (20) we have

56 $$\begin{aligned} \varDelta _\lambda R(z,x;\lambda )=\frac{\mathbf {g}_1^t(x)\varDelta _\lambda \left[ \varGamma ^{-1}(x;\lambda )\varGamma (z;\lambda )\right] \mathbf {f}_1(z)}{2\pi i\lambda (z-x)} \end{aligned}$$

and plugging in our calculation of $\varDelta _\lambda \left[ \varGamma ^{-1}(x;\lambda )\varGamma (z;\lambda )\right]$ gives the result. $\square$

#### Remark 5

From Theorem 5 we can immediately see that when $\lambda \in (-1/2,0)\cup (0,1/2)$ and $x,z\in (0,b_R)$,

57 $$\begin{aligned} R(z,x;\lambda _+)-R(z,x;\lambda _-)=\frac{b_Lb_R(2a_-(-|\lambda |)+1)}{\mathrm{sgn}(\lambda )\pi xz(b_R-b_L)}d_R(x;-|\lambda |)d_R(z;-|\lambda |) \end{aligned}$$

and when $$x,z\in (b_L,0)$$,

58 $$\begin{aligned} R(z,x;\lambda _+)-R(z,x;\lambda _-)=\frac{b_Lb_R(2a_-(-|\lambda |)+1)}{\mathrm{sgn}(\lambda )\pi xz(b_R-b_L)}d_L(x;-|\lambda |)d_L(z;-|\lambda |), \end{aligned}$$

where $d_R,d_L$ are defined in (37).

#### Proposition 4

The operators $\mathcal {H}_R^*\mathcal {H}_R, \mathcal {H}_L^*\mathcal {H}_L$ do not have eigenvalues.

#### Proof

We will prove this statement for $\mathcal {H}_R^*\mathcal {H}_R$ only, as the proof for $\mathcal {H}_L^*\mathcal {H}_L$ is similar. We show that the resolvent of $\mathcal {H}_R^*\mathcal {H}_R$ has no poles in the $\lambda$ plane. Recall from (29) that

59 $$\begin{aligned} \mathcal {R}_R(\lambda ^2)=\left( I-\frac{1}{\lambda ^2}\mathcal {H}_R^*\mathcal {H}_R\right) ^{-1}=I+\pi _R\hat{R}(\lambda /2)\pi _R, \end{aligned}$$

and using (2), we can see that the kernel of $\mathcal {R}_R$ is

60 $$\begin{aligned} \frac{[\varGamma ^{-1}(x;\lambda /2)\varGamma (z;\lambda /2)]_{1,2}}{\pi \lambda (x-z)}\chi _R(x)\chi _R(z). \end{aligned}$$

Using Proposition 2, we know that $\varGamma (z;\lambda /2)$ can (potentially) have a pole only when $\lambda =0,\pm 1$. Since (60) is single-valued for $x,z\in (0,b_R)$ and pole-free for $\lambda \in \overline{\mathbb {C}}{\setminus }\{0,\pm 1\}$, $\mathcal {R}_R(\lambda ^2)$ is pole-free for $\lambda ^2\in \overline{\mathbb {R}}{\setminus }\{0,1\}$. We can see that $\lambda ^2=0$ is not an eigenvalue of $\mathcal {H}_R^*\mathcal {H}_R$ because the null space of $\mathcal {H}_R$ contains only the zero vector. Likewise, it is easy to see that $\lambda ^2=1$ is not an eigenvalue as well. Otherwise $\mathcal {H}_R^*\mathcal {H}_Rf=f$ for some $f\in L^2([0,b_R])$, $f\not \equiv 0$, and the contradiction

61 $$\begin{aligned} \Vert f\Vert _{L^2([0,b_R])}&=\Vert \mathcal {H}_R^*\mathcal {H}_Rf \Vert _{L^2([0,b_R])}<\Vert \mathcal {H}^*_R\mathcal {H}_Rf \Vert _{\mathbb {R}}=\Vert \mathcal {H}_Rf \Vert _{L^2([b_L,0])} \nonumber \\&<\Vert \mathcal {H}_Rf \Vert _{\mathbb {R}}=\Vert f \Vert _{L^2([0,b_R])} \end{aligned}$$

proves the desired assertion. Here we used the convention that whenever the norm $\Vert \cdot \Vert _\mathbb {R}$ is computed, the left-most Hilbert transform inside the norm is evaluated over the entire line. $\square$

For convenience we define

62 $$\begin{aligned} D_R(z;\lambda ):=d_R(z;-|\lambda |/2), ~~ D_L(z;\lambda ):=d_L(z;-|\lambda |/2), \end{aligned}$$

where $d_L,d_R$ are defined in (37). Recall from Appendix C that $d_L(z;-|\lambda |), d_R(z;-|\lambda |)$ are single-valued for $\lambda \in (-1/2,1/2)$. Now for $y\in [b_L,0], x\in [0,b_R], \lambda ^2\in [0,1]$, define the kernels

63 $$\begin{aligned} \phi _L(y,\lambda ^2):=\frac{D_L(y;\lambda )}{\pi y|\lambda |D_R(\infty ;\lambda )}, ~~~ \phi _R(x,\lambda ^2):=\frac{-D_R(x;\lambda )}{\pi x|\lambda |D_L(\infty ;\lambda )}. \end{aligned}$$

Using Proposition 8 we can see that $\phi _L(y,\lambda ^2)={\text {O}}\left( 1\right)$ as $y\rightarrow b_L$ and $\phi _R(x,\lambda ^2)={\text {O}}\left( 1\right)$ as $x\rightarrow b_R$. We can see from (12), (13) that $h_\infty '(M_1(z))={\text {O}}\left( \sqrt{z}\right)$, $s_\infty '(M_1(z))={\text {O}}\left( \sqrt{z}\right)$ as $z\rightarrow 0$ whenever $\lambda ^2\in [0,1]$. Since both $D_R, D_L$ are linear combinations of $h_\infty ', s_\infty '$, it follows that $\phi _L(y,\lambda ^2)={\text {O}}\left( y^{-1/2}\right)$ as $y\rightarrow 0^-$ and $\phi _R(x,\lambda ^2)={\text {O}}\left( x^{-1/2}\right)$ as $x\rightarrow 0^+$. Also define the weights

64 $$\begin{aligned} \frac{d\sigma _L(\lambda ^2)}{d\lambda ^2}=\frac{|b_L|b_R(a+1/2)}{i(b_R-b_L)}D_R^2(\infty ;\lambda ), ~~~ \frac{d\sigma _R(\lambda ^2)}{d\lambda ^2}=\frac{|b_L|b_R(a+1/2)}{i(b_R-b_L)}D_L^2(\infty ;\lambda ), \end{aligned}$$

where $a:=a_-(-|\lambda |/2)$. Notice that both $\sigma _L'(\lambda ^2), \sigma _R'(\lambda ^2)$ (here ’ denotes differentiation with respect to $\lambda ^2$) are non-negative real analytic for $\lambda ^2\in (0,1)$, by Propositions 7 and 8. With (25) in mind, we can now prove the following theorem.

#### Theorem 6

The spectrum of $\mathcal {H}_R^*\mathcal {H}_R$, $\mathcal {H}_L^*\mathcal {H}_L$ is $\{\lambda ^2\in [0,1]\}$. Moreover, for $0\le \lambda ^2\le 1$, $g\in L^2([b_L,0])$, $f\in L^2([0,b_R])$, the operators

65 $$\begin{aligned} \hat{E}_{R,\lambda ^2}[f](x)&=\int _0^{\lambda ^2}\int _0^{b_R}\phi _R(x,\mu ^2)\phi _R(z,\mu ^2)f(z)~dzd\sigma _R(\mu ^2), \end{aligned}$$

66 $$\begin{aligned} \hat{E}_{L,\lambda ^2}[g](x)&=\int _0^{\lambda ^2}\int _{b_L}^0\phi _L(x,\mu ^2)\phi _L(z,\mu ^2)g(z)~dzd\sigma _L(\mu ^2), \end{aligned}$$

where $\phi _L,\phi _R$ and $\sigma _L,\sigma _R$ are defined in (63), (64), respectively, are the resolution of the identity for $\mathcal {H}_R^*\mathcal {H}_R$, $\mathcal {H}_L^*\mathcal {H}_L$, respectively.

#### Proof

We will consider only the operator $\mathcal {H}_R^*\mathcal {H}_R$. The proofs for $\mathcal {H}_L^*\mathcal {H}_L$ are completely analogous. It follows from Proposition 3 and Theorem 5 that

67 $$\begin{aligned} \mathcal {R}_{R+}(\lambda ^2)-\mathcal {R}_{R-}(\lambda ^2){\left\{ \begin{array}{ll} =0, &{}\lambda ^2\in {\mathbb {R}}{\setminus }[0,1] \\ \ne 0, &{}\lambda ^2\in [0,1], \end{array}\right. } \end{aligned}$$

where $\mathcal {R}_R$ is the resolvent of $\mathcal {H}_R^*\mathcal {H}_R$, see (29). Thus the spectral set is $\{\lambda ^2\in [0,1]\}$. It was shown in Proposition 4 that $\mathcal {H}_R^*\mathcal {H}_R$ has no eigenvalues.

Next, we construct $\hat{E}_{R,\lambda ^2}$. It was shown in Theorem 5 that $\hat{R}(\lambda )$ is single-valued for $\lambda \in \mathbb {C}{\setminus }[-1/2,1/2]$, thus from (25) and Proposition 3 we have

68 $$\begin{aligned} \hat{E}_{R,\lambda ^2}=\lim _{\epsilon \rightarrow 0^+}\lim _{\delta \rightarrow 0^+}\frac{-1}{2\pi i}\int _{\delta }^{\lambda ^2-\delta }\frac{1}{\mu ^2}\left[ \mathcal {R}_R(\mu ^2+i\epsilon )-\mathcal {R}_R(\mu ^2-i\epsilon )\right] d\mu ^2. \end{aligned}$$

We have shown in Proposition 4 that $\mathcal {H}_R^*\mathcal {H}_R$ has no eigenvalues. According to [2] section 82, the lack of eigenvalues guarantees that $\hat{E}_{R,\lambda ^2}$ has no points of discontinuity. Now returning to (68), we can take $\delta =0$ and we can move the $\epsilon$ limit inside the integral as the kernel of $\mathcal {R}_R$ has analytic continuation above and below the interval (0, 1). So from (25), (29), and Remark 3 we obtain

69 $$\begin{aligned} \hat{E}_{R,\lambda ^2}&=\frac{-1}{2\pi i}\int _0^{\lambda ^2}\frac{1}{\mu ^2}\left[ \mathcal {R}_{R+}(\mu ^2)-\mathcal {R}_{R-}(\mu ^2)\right] d\mu ^2 \nonumber \\&=\int _0^{\lambda ^2}\frac{-\text {sgn}(\mu )}{2\pi i\mu ^2}\pi _R\left[ \hat{R}_+(\mu /2)-\hat{R}_-(\mu /2)\right] \pi _Rd\mu ^2, \end{aligned}$$

and now plugging in (57) gives the result. Note that $\varDelta _{\lambda ^2}\mathcal {R}_R(\lambda ^2)=\text {sgn}(\lambda )\varDelta _\lambda \hat{R}(\lambda /2)$, because when $\lambda$ is on the upper shore of $(-1,0)$, $\lambda ^2$ is on the lower shore of (0, 1). $\square$

### Nature of the spectrum of $\mathcal {H}_R^*\mathcal {H}_R$ and $\mathcal {H}_L^*\mathcal {H}_L$

In this subsection, we show that the spectrum of $\mathcal {H}_R^*\mathcal {H}_R$ and $\mathcal {H}_L^*\mathcal {H}_L$ is simple and purely absolutely continuous. We will prove statements in this section for $\mathcal {H}_R^*\mathcal {H}_R$ only because the statements and ideas for proofs are nearly identical for $\mathcal {H}_L^*\mathcal {H}_L$. Notice that the resolution of the identity of $\mathcal {H}_R^*\mathcal {H}_R$ [see (65)] can be compactly written as

70 $$\begin{aligned} \hat{E}_{R,\lambda ^2}[f](x)=\int _0^{\lambda ^2}\phi _R(x,\mu ^2)U_R [f](\mu ^2)~d\sigma _R(\mu ^2), \end{aligned}$$

where $\phi _R, \sigma _R$ are defined in (63), (64), respectively and the operator $U_R:C_0^\infty ([0,b_R])\rightarrow C^\infty ((0,1))$ is defined as

71 $$\begin{aligned} U_R[f](\mu ^2):=\int _0^{b_R}\phi _R(z,\mu ^2)f(z)dz. \end{aligned}$$

For any interval $\varDelta \subset [0,1]$, which is at a positive distance from 0 and 1 (to avoid the singularities of $\phi _R(z,\mu ^2)$ as $\mu ^2\rightarrow 0$ or 1), we have

72 $$\begin{aligned} \big \Vert \hat{E}_{R,\varDelta }[f]\big \Vert ^2_{L^2([0,b_R])}= & {} \langle \hat{E}_{R,\varDelta }[f](\cdot ),\hat{E}_{R,\varDelta }[f](\cdot )\rangle =\langle \hat{E}_{R,\varDelta }^2[f](\cdot ),f\rangle \nonumber \\= & {} \langle \hat{E}_{R,\varDelta }[f](\cdot ),f\rangle ,\ f\in C_0^\infty ([0,b_R]), \end{aligned}$$

where we have used that $\hat{E}_{R,\varDelta }$ is a self-adjoint projection operator, see [2] p.214. Using (70) and (71), it is easy to show that

73 $$\begin{aligned} \langle \hat{E}_{R,\varDelta }[f],f\rangle =\int _{\varDelta }\left| U_R[f](\mu ^2)\right| ^2~d\sigma _R(\mu ^2)=\big \Vert U_R[f]\big \Vert ^2_{L^2(\varDelta ,\sigma _R)},\ f\in C_0^\infty ([0,b_R]). \end{aligned}$$

By (72) and (73) and by continuity, we can extend $U_R$ to all of $L^2([0,b_R])$ and

74 $$\begin{aligned} \big \Vert \hat{E}_{R,\varDelta }[f]\big \Vert ^2_{L^2([0,b_R])} = \big \Vert U_R[f]\big \Vert ^2_{L^2(\varDelta ,\sigma _R)},\ f\in L^2([0,b_R]). \end{aligned}$$

Taking the limit $\varDelta \rightarrow [0,1]$ in (72), (73) and using that $\hat{E}_{R,\lambda ^2}$ is the resolution of the identity, the spectrum is confined to [0, 1], and there are no eigenvalues, we prove the following Lemma.

#### Lemma 1

The operator $U_R$ extends to an isometry from $L^2([0,b_R])\rightarrow L^2([0,1],\sigma _R)$.

We are now ready to conclude this section.

#### Theorem 7

The spectrum of $\mathcal {H}_R^*\mathcal {H}_R$, $\mathcal {H}_L^*\mathcal {H}_L$ is simple and purely absolutely continuous.

#### Proof

To prove that the spectrum is simple we will show that

75 $$\begin{aligned} g(x):=\chi _R(x) \end{aligned}$$

is a generating vector. So for any $f\in L^2([0,b_R])$ we want to show that

76 $$\begin{aligned} \lim _{n\rightarrow \infty } \bigg \Vert f-\sum _{j=1}^n\alpha _{jn}\hat{E}_{R,I_{jn}}[g](x) \bigg \Vert _{L^2([0,b_R])}=0, \end{aligned}$$

where $\alpha _{jn}$ are some constants, and $I_{jn}:=[(j-1)/n,j/n)$. Thus, the intervals $I_{jn}$, $1\le j\le n$, partition the spectral interval [0, 1]. Using the properties of $\hat{E}_{R,\lambda ^2}$, we calculate

77 $$\begin{aligned}&f(x)-\sum _{j=1}^n\alpha _{jn}\hat{E}_{R,I_{jn}}[g](x)\nonumber \\&\quad =\int _0^1\phi _R(x,\mu ^2)\left\{ U_R[f](\mu ^2)-\tilde{\phi }_n(\mu ^2)U_R[g](\mu ^2)\right\} ~d\sigma _R(\mu ^2) \end{aligned}$$

where $\tilde{\phi }_n$ is the simple function

78 $$\begin{aligned} \tilde{\phi }_n(\mu ^2)=\sum _{j=1}^n \alpha _{jn}\chi _{I_{jn}}(\mu ^2). \end{aligned}$$

Using the properties of $\hat{E}_{R,\lambda ^2}$, we can write the left hand side of (77) as

79 $$\begin{aligned} \sum _{j=1}^n\hat{E}_{R,I_{jn}}[f-\alpha _{jn}g](x). \end{aligned}$$

Now using (74), (78), (79), and the fact that $\hat{E}_{R,\varDelta _j}\hat{E}_{R,\varDelta _k}=0$ whenever $\varDelta _j\cap \varDelta _k=\emptyset$ (see [2], p. 214), we see that

80 $$\begin{aligned}&\bigg \Vert f-\sum _{j=1}^n\alpha _{jn}\hat{E}_{R,I_{jn}}[g](x) \bigg \Vert _{L^2([0,b_R])}^2=\bigg \Vert \sum _{j=1}^n\hat{E}_{R,I_{jn}}[f-\alpha _{jn}g](x)\bigg \Vert _{L^2([0,b_R])}^2 \nonumber \\&\quad = \sum _{j=1}^n\big \Vert \hat{E}_{R,I_{jn}}[f-\alpha _{jn}g](x)\big \Vert _{L^2([0,b_R])}^2 =\sum _{j=1}^n\big \Vert U_R[f]-\alpha _{jn}U_R[g]\big \Vert _{L^2(I_{jn},\sigma _R)}^2\nonumber \\&\quad =\big \Vert U_R[f]-\tilde{\phi }_n U_R[g]\big \Vert _{L^2([0,1],\sigma _R)}^2, \end{aligned}$$

since the intervals $I_{jn}$ are disjoint. Now our goal is to show that any $U_R[f]\in L^2([0,1],\sigma _R)$ can be approximated by $\tilde{\phi }_n U_R[g]$. Using statement (6) of Proposition 8 in Appendix C and (62), (63), it follows that

81 $$\begin{aligned} U_R[g](\lambda ^2)=\int _0^{b_R}\phi _R(x,\lambda ^2)g(x)dx=1. \end{aligned}$$

It is clear that any $U_R[f]$ can be approximated by a sequence of simple function $\tilde{\phi }_n$, so we have

82 $$\begin{aligned} \bigg \Vert f-\sum _{j=1}^n\alpha _{jn}\hat{E}_{R,I_{jn}}[g](x) \bigg \Vert _{L^2([0,b_R])}^2=\big \Vert U_R[f]-\tilde{\phi }_n\big \Vert _{L^2([0,1],\sigma _R)}^2\rightarrow 0,\ n\rightarrow \infty , \end{aligned}$$

as desired. Thus, the spectrum of $\mathcal {H}_R^*\mathcal {H}_R$ is simple and $g=\chi _R$ is a generating vector.

Lastly, to show that the spectrum of $\mathcal {H}^*_R\mathcal {H}_R$ is purely absolutely continuous, we need to show that the function

83 $$\begin{aligned} \sigma _f(\lambda ^2):=\langle \hat{E}_{R,\lambda ^2}[f],f\rangle \end{aligned}$$

is absolutely continuous for any $f\in C_0^\infty ([0,b_R])$ (such functions are dense in $L^2([0,b_R])$), see [2], Vol. 2, Section 95. Similarly to the proof of Lemma 1, we have

84 $$\begin{aligned} \sigma _f(\lambda ^2)=\int _0^{\lambda _0^2}\left| U_R[f](\mu ^2)\right| ^2d\sigma _R(\mu ^2)+\int _{\lambda _0^2}^{\lambda ^2}\left| U_R[f](\mu ^2)\right| ^2d\sigma _R(\mu ^2) \end{aligned}$$

for any $\lambda _0\in (0,1)$. The operator $\mathcal {H}^*_R\mathcal {H}_R$ has no eigenvalues, so $\sigma _f(\lambda ^2)$ is continuous. Hence it suffices to show that $d\sigma _f(\lambda ^2)/d\lambda ^2$ is continuous on any interval $[\epsilon ,1-\epsilon ]$, $0<\epsilon <1$. Since both $d\sigma _R(\mu ^2)/d\mu ^2$ and the kernel of $U_R$ are real analytic for $\mu ^2\in (0,1)$ (see Proposition 8) and $f\in C_0^\infty ([0,b_R])$, the desired assertion follows immediately. $\square$

### Diagonalization of $\mathcal {H}_R^*\mathcal {H}_R$ and $\mathcal {H}_L^*\mathcal {H}_L$

We are now ready to use Theorem 3 and build the unitary operators which will diagonalize $\mathcal {H}_R^*\mathcal {H}_R$ and $\mathcal {H}_L^*\mathcal {H}_L$. Recall from Theorem 7 that $\chi _R, \chi _L$ are generating vectors for $\mathcal {H}_R^*\mathcal {H}_R, \mathcal {H}_L^*\mathcal {H}_L$, respectively. Following Theorem 3 and Remark 2, we define $U_L^*:L^2([0,1],\sigma _{\chi _L})\rightarrow L^2([b_L,0])$ and ${U_R^*:L^2([0,1],\sigma _{\chi _R})\rightarrow L^2([0,b_R])}$ by

85 $$\begin{aligned} U_L^*[\tilde{g}](y):=\int _0^1 \tilde{g}(\lambda ^2)~d\hat{E}_{L,\lambda ^2}[\chi _L](y), ~~~ U_R^*[\tilde{f}](x):=\int _0^1 \tilde{f}(\lambda ^2)~d\hat{E}_{R,\lambda ^2}[\chi _R](x), \end{aligned}$$

where $\hat{E}_{L,\lambda ^2},\hat{E}_{R,\lambda ^2}$ are the resolutions of the identity for $\mathcal {H}_L^*\mathcal {H}_L, \mathcal {H}_R^*\mathcal {H}_R$, respectively, see (65), (66). The spectral measures are $\sigma _{\chi _L}(\lambda ^2):=\langle \hat{E}_{L,\lambda ^2}[\chi _L],\chi _L\rangle$ and $\sigma _{\chi _R}(\lambda ^2):=\langle \hat{E}_{R,\lambda ^2}[\chi _R],\chi _R\rangle$.

#### Remark 6

Using Proposition 8, it can be verified that

86 $$\begin{aligned} \frac{d\sigma _{\chi _L}(\lambda ^2)}{d\lambda ^2}=\frac{d\sigma _L(\lambda ^2)}{d\lambda ^2}, ~~~ \frac{d\sigma _{\chi _R}(\lambda ^2)}{d\lambda ^2}=\frac{d\sigma _R(\lambda ^2)}{d\lambda ^2}, \end{aligned}$$

where $\sigma _L'(\lambda ^2), \sigma _R'(\lambda ^2)$ (here $'$ denotes differentiation with respect to $\lambda ^2$) are defined in (64).

#### Remark 7

Again, using Proposition 8, it can be verified that

87 $$\begin{aligned} U_L^*[\tilde{g}](y)=\int _0^1\phi _L(y,\lambda )\tilde{g}(\lambda ^2)~d\sigma _L(\lambda ^2), ~~~ U_R^*[\tilde{f}](x)=\int _0^1\phi _R(x,\lambda )\tilde{f}(\lambda ^2)~d\sigma _R(\lambda ^2), \end{aligned}$$

where $\phi _L, \phi _R$ are defined in (63). It is now clear that the adjoint of $U_R$, defined in (71), is $U_R^*$, and

88 $$\begin{aligned} U_L[g](\lambda ^2)=\int _{b_L}^0\phi _L(y,\lambda ^2)g(y)~dy. \end{aligned}$$

We conclude this section with the following main result.

#### Theorem 8

The operators $U_R:L^2([0,b_R])\rightarrow L^2([0,1],\sigma _R)$, $U_L:L^2([b_L,0])\rightarrow L^2([0,1],\sigma _L)$, defined in (71), (88), respectively, are unitary and

89 $$\begin{aligned} U_R\mathcal {H}_R^*\mathcal {H}_RU_R^*=\lambda ^2, ~~ U_L\mathcal {H}_L^*\mathcal {H}_LU_L^*=\lambda ^2 \end{aligned}$$

in the sense of operator equality on $L^2([0,1],\sigma _R)$, $L^2([0,1],\sigma _L)$, respectively, where $\lambda ^2$ is to be understood as a multiplication operator.

#### Proof

We state the proof for $\mathcal {H}_R^*\mathcal {H}_R$ only as the proof for $\mathcal {H}_L^*\mathcal {H}_L$ is nearly identical. The resolution of the identity of $\mathcal {H}_R^*\mathcal {H}_R$ was constructed in Theorems 6 and in Theorem 7 it was shown that the spectrum of $\mathcal {H}_R^*\mathcal {H}_R$ is simple and $\chi _R$ is a generating vector. Notice that both $U_R^*$, defined in (85), and $\sigma _{\chi _R}=\sigma _R$, defined in (64), were constructed in accordance with Theorem 3 and Remark 2. The combination of Remarks 6, 7 and Theorem 3 complete the proof. $\square$

We later obtain a different proof of this Theorem, see Corollary 1 and Theorem 13.

## Diagonalization of $\mathcal {H}_R, \mathcal {H}_L$ via Titchmarsh–Weyl theory

Using recent developments in the Titchmarsh-Weyl theory obtained in [13], it was shown in [19] that the operator

90 $$\begin{aligned} Lf(x):=\left[ P(x)f'(x)\right] '+2\left( x-\frac{b_R+b_L}{4}\right) ^2f(x), ~~ P(x):=x^2(x-b_L)(x-b_R) \end{aligned}$$

has only continuous spectrum and commutes with the FHTs $\mathcal {H}_L,\mathcal {H}_R$, defined in (2). We now state the main result of [19] and refer the reader to this paper for more details.

### Theorem 9

The operators $U_1:L^2([b_L,0])\rightarrow L^2(J,\rho _1)$ and $U_2:L^2([0,b_R])\rightarrow L^2(J,\rho _2)$, where $J=\left[ (b_L^2+b_R^2)/8,\infty \right)$, are isometric transformations. Moreover, in the sense of operator equality on $L^2(J,\rho _2)$ one has

91 $$\begin{aligned} U_2\mathcal {H}_LU_1^*=\sigma (\omega ), \end{aligned}$$

where

92 $$\begin{aligned} \sigma (\omega )=\frac{-b_R}{b_L\cosh (\mu (\omega )\pi )}\left( 1+{\text {O}}\left( \epsilon ^{\frac{1}{2}-\delta }\right) \right) , ~~~ \omega \rightarrow \infty ,~~~ \epsilon =\omega ^{-1/2}, \end{aligned}$$

$\mu (\omega )=\sqrt{\frac{\omega -(b_L+b_R)^2/8}{-b_Lb_R}-\frac{1}{4}}$, $\rho _1'(\omega )=\frac{1}{b_L^2(b_R-b_L)}\left( 1+{\text {O}}\left( \epsilon ^{\frac{1}{2}-\delta }\right) \right)$, $\rho _2'(\omega )=\frac{1}{b_R^2(b_R-b_L)}\left( 1+{\text {O}}\left( \epsilon ^{\frac{1}{2}-\delta }\right) \right)$, and $0<\delta<<1$ is fixed.

There is a minor typo in this theorem in [19]; when describing $\sigma (\lambda )$, the factor $\frac{a_2^3}{a_1}$ is incorrect and should be $-\frac{a_2}{a_1}$. The operators $U_1, U_2$ in Theorem 9 were obtained asymptotically when $\omega \rightarrow \infty$. Here we obtain these operators explicitly. According to [13], the kernels of $U_1, U_2$ and the spectral measures $\rho _1, \rho _2$ are defined through particular solutions of $Lf=\omega f$. Such solutions will be constructed in the following subsections.

### Interval $[0,b_R]$

Define the function93where $M_4(x)$ and $\mu$ are defined in Remark 4 and Theorem 9, respectively. Notice that $M_4(x)$ maps $b_L\rightarrow -1, 0\rightarrow 0, b_R\rightarrow 1$. If we take $b_R=-b_L=a$ in (93), where *a* is a constant, we obtain (4.9) of [19]. Now define

94 $$\begin{aligned} \varphi _2(x,\omega ):=kf_R(x,\omega )+\overline{k}\overline{f}_R(x,\omega ), ~~~ \vartheta _2(x,\omega ):=l_2f_R(x,\omega )+\overline{l}_2\overline{f}_R(x,\omega ), \end{aligned}$$

where

95 $$\begin{aligned} k&:=\frac{\varGamma (-i\mu )}{\varGamma \left( \frac{1}{4}-\frac{i\mu }{2}\right) \varGamma \left( \frac{3}{4}-\frac{i\mu }{2}\right) },\nonumber \\ l_2&:=\frac{k}{b_R^2(b_R-b_L)}\left[ 2\gamma +2\varPsi \left( \frac{1}{2}-i\mu \right) +\ln \left( \frac{-b_L}{b_R(b_R-b_L)}\right) \right] . \end{aligned}$$

Here $\gamma$ is Euler’s constant and $\varPsi$ is the Digamma function, see [1] 6.3.1.

#### Remark 8

Using properties of the Gamma functions, see [1] 6.1.30, it can be shown that

96 $$\begin{aligned} |k|^2=\frac{\coth (\mu \pi )}{2\pi \mu }, \end{aligned}$$

provided $$\mu \ge 0$$.

#### Remark 9

Notice that if we take

97 $$\begin{aligned} i\mu (\omega )=a+\frac{1}{2}\implies \omega =\frac{(b_R+b_L)^2}{8}+b_Lb_Ra(a+1) \end{aligned}$$

for $\lambda \in [-1,1]$ (this implies $\mu \in [0,\infty )$ iff $\omega \in [(b_L^2+b_R^2)/8,\infty )$), where $a:=a_-(-|\lambda |/2)$, it can be verified via [1] 15.3.16 that

98 $$\begin{aligned} \frac{-b_R\alpha (-|\lambda |/2)}{x\sqrt{\pi }}h_\infty '\left( M_1(x)\right) =kf_R(x,\omega ), ~~ \frac{-b_R\beta (-|\lambda |/2)}{x\sqrt{\pi }}s_\infty '\left( M_1(x)\right) =\overline{k}\overline{f}_R(x,\omega ) \end{aligned}$$

where $M_1(x)$ is defined in Remark 4, $\alpha ,\beta$ and $h_\infty ',s_\infty '$ are defined in (39) and (12), (13), respectively. This relation immediately implies that

99 $$\begin{aligned} \varphi _2(x,\omega )=\frac{-b_R}{x\sqrt{\pi }}D_R(x,\lambda ) \end{aligned}$$

for $\lambda \in [-1,1]$, see (62) for $D_R(x,\lambda )$.

#### Theorem 10

The functions $\varphi _2, \vartheta _2$ defined in (94) satisfy the following properties:

1. For $x\in [0,b_R]$ and $\omega \in [(b_L^2+b_R^2)/8,\infty )$, $\varphi _2(x,\omega ),\vartheta _2(x,\omega )$ are linearly independent solutions of $Lf=\omega f$, where *L* is defined in (90),
2. $$\varphi _2(x,\omega ),\vartheta _2(x,\omega )\in \mathbb {R}$$, for all $$x\in [0,b_R]$$, $$\omega \in \mathbb {R}$$,
3. $$P(x)\varphi _2'(x,\omega )\rightarrow 0$$ as $$x\rightarrow b_R^-$$,
4. $$P(x)W_x(\vartheta _2(x,\omega ),\varphi _2(x,\omega ))=1$$ for all $$x\in [0,b_R]$$, $$\omega \in \mathbb {C}$$,
5. $$\displaystyle {\lim _{x\rightarrow b_R^-}P(x)W_x(\vartheta _2(x,\omega ),\varphi _2(x,\omega '))=1}$$ for all $$\omega ,\omega '\in \mathbb {C}$$.

#### Proof

1. By definition, $\varphi _2, \vartheta _2$ are linear combinations of $f_R$ and $\overline{f}_R$, which can be expressed in terms of $h_\infty ', s_\infty '$, see Remark 9. Recall that $h_\infty (x), s_\infty (x)$ are solutions of (14). It can now be verified that $\frac{1}{x}h_\infty '\left( M_1(x)\right) , \frac{1}{x}s_\infty '\left( M_1(x)\right)$ are solutions of
  100 $$\begin{aligned} L[g](x)=\left[ \frac{(b_L+b_R)^2}{8}+b_Lb_R\cdot a(a+1)\right] g(x), \end{aligned}$$
  where $a:=a_-(-|\lambda |/2)$. Thus $\varphi _2, \vartheta _2$ solve $Lf=\omega f$ for $x\in [0,b_R]$ and $\omega \in [(b_L^2+b_R^2)/8,\infty )$. To show $\varphi _2, \vartheta _2$ are linearly independent, we compute their Wronskian. It is a simple exercise to show that
  101 $$\begin{aligned} W_x[\vartheta _2,\varphi _2]=2i\mathfrak {I}[\overline{k}l_2]W_x[f_R,\overline{f}_R]. \end{aligned}$$
  Using Remark 8, [1] 6.3.12 and Remark 9, (210), (224), it can be verified that
  102 $$\begin{aligned} \mathfrak {I}[\overline{k}l_2]=\frac{-1}{2\mu b_R^2(b_R-b_L)}, ~~~ W_x[f_R,\overline{f}_R]=\frac{i\mu b_R^2(b_R-b_L)}{P(x)}. \end{aligned}$$
  Thus $\varphi _2, \vartheta _2$ are linearly independent since $W_x[\vartheta _2,\varphi _2]=1/P(x)$.
2. This is clear by the definition of $\vartheta _2,\varphi _2$, see (94).
3. This follows from Remark 9 and Proposition 8 because $D_R(x,\lambda )$ is analytic at $x=b_R$. Moreover, using [1] 15.3.6, it can be shown that $\varphi _2(b_R,\omega )=1$.
4. We have previously shown that $P(x)W_x[\vartheta _2(x,\omega ),\varphi _2(x,\omega )]=1$ for $x\in (0,b_R)$, ${\omega \in [(b_L^2+b_R^2)/8,\infty )}$. This can be extended to the entire complex $\omega$ plane.
5. Using [1] 15.3.6, 15.3.10 and Remark 9, for $\omega \in ((b_L^2+b_R^2)/8,\infty )$ (which implies $\mu \in (0,\infty )$), we have
  103 $$\begin{aligned} \varphi _2(x,\omega )&=1+{\text {O}}\left( x-b_R\right) , ~~~ \vartheta _2(x,\omega )=-2\mathfrak {R}[i\mu l_2\overline{k}]\ln (b_R-x)+\text {o}(1) \end{aligned}$$
  as $x\rightarrow b_R^-$. Thus for $\omega ,\omega '\in ((b_L^2+b_R^2)/8,\infty )$ we have
  104 $$\begin{aligned} W_x\left[ \vartheta _2(x,\omega ),\varphi _2(x,\omega ')\right] =\frac{2\mathfrak {R}[i\mu l_2\overline{k}]}{b_R-x}+{\text {O}}\left( \ln (b_R-x)\right) \end{aligned}$$
  as $x\rightarrow b_R^-$. So by (104),
  105 $$\begin{aligned} \lim _{x\rightarrow b_R^-}P(x)W_x\left[ \vartheta _2(x,\omega ),\varphi _2(x,\omega ')\right] =1, \end{aligned}$$
  which holds for $\omega ,\omega '\in ((b_L^2+b_R^2)/8,\infty )$, and can be extended to any $\omega ,\omega '\in \mathbb {C}$.

$$\square $$

### Interval $[b_L,0]$

This subsection will be similar to the last so many proofs will be omitted, as the ideas have been previously presented. Define the function106where $M_4(x)$ and $\mu (\omega )$ are defined in Remark 4 and Theorem 9, respectively. Now define

107 $$\begin{aligned} \varphi _1(x,\omega ):=kf_L(x,\omega )+\overline{k}\overline{f}_L(x,\omega ), ~~~ \vartheta _1(x,\omega ):=l_1f_L(x,\omega )+\overline{l}_1\overline{f}_L(x,\omega ), \end{aligned}$$

where *k* is defined in (95) and

108 $$\begin{aligned} l_1:=\frac{k}{b_L^2(b_R-b_L)}\left[ 2\gamma +2\varPsi \left( \frac{1}{2}-i\mu \right) +\ln \left( \frac{-b_R}{b_L(b_R-b_L)}\right) \right] . \end{aligned}$$

#### Remark 10

The functions $f_L, f_R$, defined in (106), (93), respectively, share the relation

109 $$\begin{aligned} \frac{b_L^2f_R\left( M_2(x),\omega \right) }{x(b_R+b_L)-b_Lb_R}=f_L(x,\omega ), \end{aligned}$$

where $M_2(x)$ is defined in (4). This relation combined with Remark 9 shows that

110 $$\begin{aligned} \varphi _1(x,\omega )=\frac{b_LM_2(x)}{b_Rx}\varphi _2(M_2(x),\omega )=\frac{-b_L}{x\sqrt{\pi }}D_L(x,\lambda ), \end{aligned}$$

where $D_L, \varphi _2$ are defined in (62), (94), respectively, and the relation between $\omega$ and $\lambda$ is described in (97).

#### Theorem 11

The functions $\varphi _1, \vartheta _1$ defined in (94) satisfy the following properties:

1. For $x\in [b_L,0]$ and $\omega \in [(b_L^2+b_R^2)/8,\infty )$, $\varphi _1(x,\omega ), \vartheta _1(x,\omega )$ are linearly independent solutions of $Lf=\omega f$, where *L* is defined in (90),
2. $$\varphi _1(x,\omega ),\vartheta _1(x,\omega )\in \mathbb {R}$$, for all $$x\in [b_L,0]$$, $$\omega \in \mathbb {R}$$,
3. $$P(x)\varphi _1'(x,\omega )\rightarrow 0$$ as $$x\rightarrow b_L^+$$,
4. $$-P(x)W_x(\vartheta _1(x,\omega ),\varphi _1(x,\omega ))=1$$ for all $$x\in [b_L,0]$$, $$\omega \in \mathbb {C}$$,
5. $$\displaystyle {\lim _{x\rightarrow b_L^+}-P(x)W_x(\vartheta _1(x,\omega ),\varphi _1(x,\omega '))=1}$$ for all $$\omega ,\omega '\in \mathbb {C}$$.

### Diagonalization of $\mathcal {H}_L, \mathcal {H}_R$

According to the spectral theory developed in [13], we have gathered nearly all the necessary ingredients to diagonalize $\mathcal {H}_L, \mathcal {H}_R$. It remains to construct two functions $m_1(\omega )$ and $m_2(\omega )$ so that

111 $$\begin{aligned} \vartheta _1(x,\omega )+m_1(\omega )\varphi _1(x,\omega )\in L^2([b_L,0]), ~~~ \vartheta _2(x,\omega )+m_2(\omega )\varphi _2(x,\omega )\in L^2([0,b_R]) \end{aligned}$$

whenever $\mathfrak {I}\omega >0$. It can be verified that

112 $$\begin{aligned} m_j=-\frac{l_j}{k}, ~~ j=1,2 \end{aligned}$$

where $l_1$ and $l_2, k$ are defined in (108), (95), respectively. The spectral measures $\rho _1,\rho _2$ are constructed via the formula

113 $$\begin{aligned} \rho _j(\omega _2)-\rho _j(\omega _1)=\lim _{\epsilon \rightarrow 0^+}\frac{1}{\pi }\int _{\omega _1}^{\omega _2}\mathfrak {I}m_j(s+i\epsilon )~ ds, \end{aligned}$$

for $j=1,2$ (see [13] for more details). From (113) we obtain

114 $$\begin{aligned} \rho _1'(\omega )=\frac{\tanh (\mu \pi )}{b_L^2(b_R-b_L)}, ~~~ \rho _2'(\omega )=\frac{\tanh (\mu \pi )}{b_R^2(b_R-b_L)}, \end{aligned}$$

where we have used (112), Remark 8, and (102). Define the operators $U_1:L^2([b_L,0])\rightarrow L^2(J,\rho _1)$ and $U_2:L^2([0,b_R])\rightarrow L^2(J,\rho _2)$, where $J=\left( \frac{b_R^2+b_L^2}{8},\infty \right)$, as

115 $$\begin{aligned} U_1[f](\omega )&:=\int _{b_L}^0\varphi _1(x,\omega )f(x)~dx, ~~~ U_1^*[f](\omega )=\int _J\varphi _1(x,\omega )\tilde{f}(\omega )~d\rho _1(\omega ), \end{aligned}$$

116 $$\begin{aligned} U_2[f](\omega )&:=\int _0^{b_R}\varphi _2(x,\omega )f(x)~dx, ~~~ U_2^*[\tilde{f}](\omega )=\int _J\varphi _2(x,\omega )\tilde{f}(\omega )~d\rho _2(\omega ), \end{aligned}$$

where $\varphi _1,\varphi _2$ are defined in (107), (94), respectively. We are now ready to prove the main result of this section.

#### Theorem 12

The operators $U_1, U_2$, defined in (115), (116), are unitary and in the sense of operator equality on $L^2(J,\rho _2)$, where $J=\left( (b_R^2+b_L^2)/8,\infty \right)$, one has

117 $$\begin{aligned} U_2\mathcal {H}_LU_1^*=-\frac{b_R}{b_L}{} \mathrm{sech}(\mu \pi ), \end{aligned}$$

where $\rho _2'$ is defined in (114).

#### Proof

First, the operators $U_1, U_2$ are unitary by [13]. According to Proposition 8 and Remark 10,

118 $$\begin{aligned} \mathcal {H}_L[\varphi _1](y,\omega )=\mathcal {H}_L\left[ \frac{-b_LD_L(x;\lambda )}{x\sqrt{\pi }}\right] (y,\omega )=\frac{-b_L}{b_R}\text {sech}(\mu \pi )\varphi _2(x,\omega ), \end{aligned}$$

since (97) implies that $|\lambda |=\text {sech}(\mu \pi )$. Using (118), we calculate that

119 $$\begin{aligned} \mathcal {H}_LU_1^*[\tilde{f}](x)=U_2^*\left[ \frac{-b_L\rho _1'(\omega )}{b_R\rho _2'(\omega )}\text {sech}(\mu \pi )\tilde{f}(\omega )\right] (x) \end{aligned}$$

for any $\tilde{f}\in L^2(J,\rho _1)$, which is equivalent to

120 $$\begin{aligned} U_2\mathcal {H}_LU_1^*=-\frac{b_R}{b_L}\text {sech}(\mu \pi ). \end{aligned}$$

$$\square $$

Since the adjoint of $\mathcal {H}_L$ is $-\mathcal {H}_R$, we have an immediate Corollary.

#### Corollary 1

In the sense of operator equality on $L^2(J,\rho _1)$ one has

121 $$\begin{aligned} U_1\mathcal {H}_RU_2^*=\frac{b_L}{b_R}{} \mathrm{sech}(\mu \pi ), ~~ U_1\mathcal {H}_L^*\mathcal {H}_LU_1^*=\mathrm{sech}^2(\mu \pi ), \end{aligned}$$

and in the sense of operator equality on $L^2(J,\rho _2)$ one has

122 $$\begin{aligned} U_2\mathcal {H}_R^*\mathcal {H}_RU_2^*=\mathrm{sech}^2(\mu \pi ). \end{aligned}$$

#### Proof

The proof follows quickly from Theorem 12 because

123 $$\begin{aligned} (U_2\mathcal {H}_LU_1^*)^*=U_1\mathcal {H}_L^*U_2^* \end{aligned}$$

and (what follows is the multiplication operator)

124 $$\begin{aligned} \left( -\frac{b_R}{b_L}\text {sech}(\mu \pi )\right) ^*=-\frac{b_R}{b_L}\text {sech}(\mu \pi )\cdot \frac{\rho _2'(\omega )}{\rho _1'(\omega )}=-\frac{b_L}{b_R}\text {sech}(\mu \pi ). \end{aligned}$$

$$\square $$

This corollary can be used to recover Theorem 8. We have now obtained two (seemingly) different diagonalizations of $\mathcal {H}^*_R\mathcal {H}_R$ and $\mathcal {H}^*_L\mathcal {H}_L$ in Theorem 8 and Corollary 1. We show that these diagonalizations are equivalent in the sense of change of spectral variable.

#### Theorem 13

The two diagonalizations of $\mathcal {H}^*_L\mathcal {H}_L$ obtained in Theorem 8 and Corollary1 are equivalent; that is,

125 $$\begin{aligned} U_1^*\mathrm{sech}^2(\mu \pi )U_1=U_L^*\lambda ^2U_L \end{aligned}$$

in the sense of operator equality on $L^2([b_L,0])$. The operators $U_1,U_L$ are defined in (115), (88), respectively and $\mathrm{sech}^2(\mu \pi ),\lambda ^2$ are to be understood as multiplication operators. An identical statement about $U_2$ and $U_R$, defined in (116), (71), respectively, can be made.

#### Proof

We will relate the operators $U_L,U_1$ by using the change of variable $\lambda \rightarrow \omega$ in (97), which implies that

126 $$\begin{aligned} \text {sech}^2(\mu (\omega )\pi )=\lambda ^2, ~~~ \frac{b_R(a+1/2)}{i\pi \lambda ^2b_L(b_R-b_L)}d\lambda ^2=d\rho _1(\omega ). \end{aligned}$$

Now using this change of variable, we see that

127 $$\begin{aligned} U_L^*[\tilde{f}](x)=U_1^*[c(\omega )\tilde{f}(\text {sech}^2(\mu \pi )](x), \end{aligned}$$

where $$c(\omega )=-b_L\sqrt{\pi }|\lambda |D_R(\infty ;\lambda )$$. Similarly,

128 $$\begin{aligned} U_L[f](\lambda ^2)=\frac{1}{c(\omega )}U_1[f](\omega ). \end{aligned}$$

So using (127) and (128) we obtain

129 $$\begin{aligned} U_L^*\lambda ^2U_L[f](x)&=U_1^*\text {sech}^2(\mu \pi )U_1[f](x), \end{aligned}$$

as desired. $$\square $$

## Small $\lambda$ asymptotics of $\varGamma (z;\lambda )$

In this section we only consider the symmetric scenario when $b_R=-b_L=1$, but the results can be obtained for general endpoints via Möbius transformations. The main results of this section are Theorems 15 and 17, which describes the small $\lambda$ asymptotics of $\varGamma (z;\lambda )$ first in a small annulus around $z=0$ and then in the rest of $\mathbb {C}$ respectively. The proof of Theorem 15 is based on the asymptotics of hypergeometric functions that appear in $\varGamma (z;\lambda )$, whereas the prove of Theorem 17 is based on Theorem 15 and the Deift–Zhou nonlinear steepest descent method [10].

### Remark 11

Everywhere in this section we consider $\lambda \in \mathbb {C}{\setminus }[-1/2,1/2]$, where $\lambda$ on upper/lower shores of $[-1/2,1/2]$ is also allowed; such values will be denoted by $\lambda _\pm$ respectively.

### Modified saddle point method uniform with respect to parameters

According to (17), we are interested in $h_\infty (\eta )$, where

130 $$\begin{aligned} \eta =\frac{z+1}{2z}. \end{aligned}$$

In view of the integral representation ([1], 15.3.1)131of $h_\infty (\eta )$, given by (12), where $a(\lambda )\rightarrow \infty$ as $\lambda \rightarrow 0$, we want to use the saddle point method to find the small $\lambda$ asymptotics of $h_\infty (\eta )$. We start with the case $\mathfrak {I}\lambda \ge 0$ which implies $\mathfrak {I}[a]\rightarrow -\infty$ as $\lambda \rightarrow 0$, see Appendix B for more information about $a(\lambda )$. For $\mathfrak {I}\lambda \le 0$ the results are similar, see Remark 18. With that in mind, define function

132 $$\begin{aligned} S_\eta (t)=S\left( t,\eta \right) :=-i\ln \left( \frac{t(1-t)}{1-\frac{t}{\eta }}\right) \end{aligned}$$

where the branch cuts of $S_\eta (t)$ in *t* variable are chosen to be $(-\infty ,0)$, $(1,\infty )$, and the ray from $t=\eta$ to $t=\infty$ with angle $\arg {\eta }$. The integral from (131) can be now written as

133 $$\begin{aligned} \int _0^1\left( \frac{t(1-t)}{1-\frac{t}{\eta }}\right) ^{a(\lambda )}~dt=\int _0^1e^{i\mathfrak {R}[a(\lambda )]S_\eta (t)}e^{-\mathfrak {I}[a(\lambda )]S_\eta (t)}~dt. \end{aligned}$$

Define closed regions

134 $$\begin{aligned} \varOmega&:=\left\{ \eta =\frac{z+1}{2z}:~M\le |\eta |\le 2M\right\} , \end{aligned}$$

135 $$\begin{aligned} \varOmega _+&:=\left\{ \eta =\frac{z+1}{2z}:~M\le |\eta |\le 2M, ~ 0\le \arg (\eta )\le \pi \right\} , \end{aligned}$$

where *M* is a large, positive, fixed number that is to be determined. Notice that the set of all *z* such that $\frac{z+1}{2z}\in \varOmega$ is a small annulus about the origin. The large *a* asymptotics of the integral in (133) that is uniform in $\eta \in \varOmega$ is technically not covered by standard saddle point theorems (see, for example, [12, 25, 30]). Therefore, in Appendix D we present a proof of Theorem 14 for such integrals, that will be used later for the small lambda asymptotics of hypergeometric functions $h_\infty (\eta ), s_\infty (\eta )$ and their derivatives. The obtained results in Theorem 14 leading order term of the hypergeometric function is consistent with the results of Paris [26], where the formal asymptotic expansion in the large parameter $a(\lambda )$ was derived, but the error estimates and uniformity in $\eta$ were not addressed.

The following proposition identifying the saddle points of $S_\eta (t)$ is a simple exercise. We need the saddle point $t_-^*(\eta )$ to state Theorem 14.

#### Proposition 5

For $\eta \in \varOmega _+$, the function $S_\eta (t)$ has exactly two simple saddle points $t_\pm ^*(\eta )$ defined by $S_\eta '(t_\pm ^*(\eta ))=0$. Explicitly,

136 $$\begin{aligned} t_+^*(\eta )&=\eta +\sqrt{\eta ^2-\eta }=2\eta +{\text {O}}\left( 1\right) ~ \text { as } \eta \rightarrow \infty , \end{aligned}$$

137 $$\begin{aligned} t_-^*(\eta )&=\eta -\sqrt{\eta ^2-\eta }=\frac{1}{2}+{\text {O}}\left( \eta ^{-1}\right) ~ \text { as } \eta \rightarrow \infty , \end{aligned}$$

where the branchcut for $t_\pm ^*(\eta )$ is [0, 1]. Moreover,

138 $$\begin{aligned} S_\eta (t^*_\pm (\eta ))=-2i\ln \left( t_\pm ^*(\eta )\right) ~ \text { and } ~~ S_\eta ''(t^*_-(\eta ))=\frac{2i}{t^*_-(\eta )\left( 1-t^*_-(\eta )\right) }. \end{aligned}$$

Let $B(\zeta ,r)$, $B^0(\zeta ,r)$ denote an open disc and a punctured open disc respectively of radius $r>0$ centered at $\zeta \in \mathbb {C}$.

#### Theorem 14

Fix a sufficiently small $\epsilon >0$, a sufficiently large *M* (see (134)) and suppose $F(t,\eta ,\lambda )$ satisfies the following properties:

1. For every $(\eta ,\lambda )\in \varOmega _+\times {B}^0(0,\epsilon )$ , $F(t,\eta ,\lambda )$ is analytic in $t\in B(1/2,1/2)$;
2. $F(t,\eta ,\lambda )$ is continuous in all variables in $B(1/2,1/2)\times \varOmega _+\times {B^0}(0,\epsilon )$ and for every $t\in B(1/2,1/2)$ it is bounded in $(\eta ,\lambda )\in \varOmega _+\times {B^0}(0,\epsilon )$;
3. $F(t,\eta ,\lambda )={\text {O}}\left( t^{c_0}\right)$ as $t\rightarrow 0$, where $c_0>-1$ uniformly in $(\eta ,\lambda )\in \varOmega _+\times {B^0}(0,\epsilon )$;
4. $F(t,\eta ,\lambda )={\text {O}}\left( (1-t)^{c_1}\right)$ as $t\rightarrow 1$, where $c_1>-1$ uniformly in $(\eta ,\lambda )\in \varOmega _+\times {B^0}(0,\epsilon )$;
5. $|F(t_-^*(\eta ),\eta ,\lambda )|$ is separated from zero for all $(\eta ,\lambda )\in \varOmega _+\times {B^0}(0,\epsilon )$.

Then

139 $$\begin{aligned}&\int _0^1F(t,\eta ,\lambda )e^{-\mathfrak {I}[a(\lambda )]S_\eta (t)}dt\nonumber \\&\quad =e^{-\mathfrak {I}[a(\lambda )]S_\eta (t^*_-(\eta ))}F\left( t^*_-(\eta ),\eta ,\lambda \right) \sqrt{\frac{2\pi }{\mathfrak {I}[a(\lambda )]S''_\eta (t^*_-(\eta ))}}\left[ 1+{\text {O}}\left( \frac{M^2}{\mathfrak {I}[a(\lambda )]}\right) \right] , \end{aligned}$$

as $\lambda \rightarrow 0$, where $a(\lambda )$ is defined in (15). This approximation is uniform for $\eta \in \varOmega _+$.

The idea of the proof is as follows: we deform the contour of integration in (139) from [0, 1] to a path we call $\gamma _\eta$, which passes through a relevant saddle point $t_-^*(\eta )$ of $S_\eta (t)$. We then show that the leading order contribution in (139) comes from a small neighborhood of $t_-^*(\eta )$.

#### Remark 12

One can simplify equation (139) in Theorem 14 by substituting (138).

### Small $\lambda$ asymptotics of $\varGamma (z;\lambda )$ for $z\in \tilde{\varOmega }$

In this subsection we use Theorem 14 to calculate the leading order asymptotics of $\varGamma (z;\lambda )$ given by (17) as $\lambda \rightarrow 0$, provided that $z\in \tilde{\varOmega }$, where

140 $$\begin{aligned} \tilde{\varOmega }:=\left\{ z:~\frac{z+1}{2z}\in \varOmega \right\} , ~~~ \tilde{\varOmega }_+:=\left\{ z:~\frac{z+1}{2z}\in \varOmega _+\right\} . \end{aligned}$$

Notice that $\tilde{\varOmega }$ is a small annulus about the origin. In this section, we will often use the variable

141 $$\begin{aligned} \varkappa =-\ln \lambda \end{aligned}$$

instead of $\lambda$ and the function

142 $$\begin{aligned} g(z):=a\left( -\frac{z}{2}\right) =\frac{1}{i\pi }\ln \left( \frac{i+\sqrt{z^2-1}}{-z}\right) . \end{aligned}$$

The properties of the *g*-function can be found in Proposition 6.

#### Corollary 2

In the limit $\lambda =e^{-\varkappa }\rightarrow 0$

143 $$\begin{aligned} h_\infty \left( \frac{z+1}{2z}\right)&= i4^a\frac{\sqrt{2z}(1-z^2)^{1/4}}{1+\sqrt{1-z^2}}e^{\pm \varkappa \left( \frac{1}{2} -g(z)\right) }\left( 1+{\text {O}}\left( \frac{M^2}{\varkappa }\right) \right) , \end{aligned}$$

144 $$\begin{aligned} h_\infty '\left( \frac{z+1}{2z}\right)&=\mp 4^a\frac{\varkappa (2z)^{\frac{3}{2}}e^{\pm \varkappa \left( \frac{1}{2}-g(z)\right) }}{\pi (1-z^2)^{1/4}(1+\sqrt{1-z^2})}\left( 1+{\text {O}}\left( \frac{M^2}{\varkappa }\right) \right) , \end{aligned}$$

145 $$\begin{aligned} s_\infty \left( \frac{z+1}{2z}\right)&= i4^{-a}\frac{(1-z^2)^{1/4}(1+\sqrt{1-z^2})}{(2z)^{\frac{3}{2}}}e^{\pm \varkappa \left( g(z)-\frac{1}{2}\right) }\left( 1+{\text {O}}\left( \frac{M^2}{\varkappa }\right) \right) , \end{aligned}$$

146 $$\begin{aligned} s_\infty '\left( \frac{z+1}{2z}\right)&=\pm 4^{-a}\frac{\varkappa (1+\sqrt{1-z^2})}{\pi \sqrt{2z}(1-z^2)^{1/4}}e^{\pm \varkappa \left( g(z)-\frac{1}{2}\right) }\left( 1+{\text {O}}\left( \frac{M^2}{\varkappa }\right) \right) , \end{aligned}$$

provided $\pm \mathfrak {I}\lambda \ge 0$, where each approximation is uniform in $z\in \tilde{\varOmega }_+$. The functions $h_\infty ,h_\infty '$ and $s_\infty ,s_\infty '$ are defined in (12), (13), respectively. The functions $\sqrt{1-z^2}$ and $(1-z^2)^{1/4}$ have branch cuts on $(-1,1)$ and $(-\infty ,1)$ respectively.

#### Proof

According to Theorem 14,

147 $$\begin{aligned} h_\infty \left( \frac{z+1}{2z}\right)&=\frac{e^{a\pi i}\varGamma (2a+2)}{\varGamma ^2(a+1)}\sqrt{\frac{\pi }{a}}\left( \frac{1-z}{1+z}\right) ^{\frac{1}{4}}\left( \frac{2z}{z+1}\right) ^{a}\left( 1+\sqrt{\frac{1-z}{1+z}}\right) ^{-1-2a}\nonumber \\&\quad \left[ 1+{\text {O}}\left( \frac{M^2}{a}\right) \right] , \end{aligned}$$

where we have used $F(t,\eta ,\lambda )=e^{i\mathfrak {R}[a(\lambda )]S_\eta (t)}$ and $\eta =\frac{z+1}{2z}$. By Proposition 7

148 $$\begin{aligned} \left( \frac{z+1}{2z}\right) ^{-a}\left( 1+\sqrt{\frac{1-z}{1+z}}\right) ^{-2a}&=\exp \left[ -a\pi ig(z)-\frac{a\pi i}{2}\right] \end{aligned}$$

149 $$\begin{aligned}&=\frac{\sqrt{z(1+z)}}{\sqrt{2}(1+\sqrt{1-z^2})}e^{-\varkappa g(z)-\varkappa /2}\left( 1+{\text {O}}\left( \lambda ^2\right) \right) \end{aligned}$$

as $\lambda \rightarrow 0$ with $\mathfrak {I}\lambda \ge 0$ and $z\in \tilde{\varOmega }_+$. Using [24] 5.5.5, 5.11.13 we have

150 $$\begin{aligned} \frac{\varGamma (2a+2)}{\varGamma (a+1)^2}=\frac{4^{a+1/2}}{\sqrt{\pi }}\cdot \frac{\varGamma \left( a+\frac{3}{2}\right) }{\varGamma (a+1)}=4^{a+1/2}\sqrt{\frac{a}{\pi }}\left( 1+{\text {O}}\left( \frac{1}{a}\right) \right) \end{aligned}$$

as $a\rightarrow -i\infty$. Now plugging (149), (150) into (147), we obtain the result for $h_\infty \left( \frac{z+1}{2z}\right)$ when $\mathfrak {I}\lambda \ge 0$. The approximation when $\mathfrak {I}\lambda \le 0$ can be found in an similar manner.

For $h_\infty '(\eta )$ we again use Theorem 14 with $F(t,\eta ,\lambda )=\frac{e^{i\mathfrak {R}[a(\lambda )]S_\eta (t)}}{1-t/\eta }$ to obtain

151 $$\begin{aligned} h_\infty '(\eta )&=-e^{a\pi i}\frac{a\varGamma (2a+2)}{\eta ^{a+1}\varGamma (a+1)^2}\int _0^1\frac{1}{1-t/\eta }\left( \frac{t(1-t)}{1-t/\eta }\right) ^adt \nonumber \\&=-2ae^{a\pi i}4^a\eta ^{-a-1}\left( 1-\frac{1}{\eta }\right) ^{-1/4}\left( 1+\sqrt{1-\frac{1}{\eta }}\right) ^{-1-2a(\lambda )}\left( 1+{\text {O}}\left( \frac{M^2}{a}\right) \right) , \end{aligned}$$

which is equivalent to the stated result. Notice that the functions $s_\infty (\eta ),s_\infty '(\eta )$, as written, only have the integral representation [1] 15.3.1 for $-1/2\le \mathfrak {R}[a(\lambda )]<0$. In this case, we obtain the stated result immediately via the observation ${h_\infty (\eta )\big |_{a\rightarrow -a-1}=s_\infty (\eta )}$. To obtain the results when $0\le \mathfrak {R}[a(\lambda )]\le 1/2$, use [24] 15.5.19 with $z\rightarrow 1/\eta$, $a\rightarrow -a$, $b\rightarrow -a-1$, and $c\rightarrow -2a$ to obtain152Now with $z\rightarrow 1/\eta$, $a\rightarrow -a+1$, $b\rightarrow -a$, and $c\rightarrow -2a+1$, we have153Combining the two previous equations, we see that154The perk of this equation is that the right hand side has an integral representation for ${-1/2\le \mathfrak {R}[a]\le 1/2}$. Thus we can apply Theorem 14 twice and obtain the leading order asymptotics. So we have shown155A similar process can be repeated for $s_\infty '(\eta )$ and we obtain156$\square$

We have an immediate Corollary.

#### Corollary 3

In the limit $\lambda =e^{-\varkappa }\rightarrow 0$,

157 $$\begin{aligned} \hat{\varGamma }\left( \frac{z+1}{2z}\right)&=i(1-z^2)^{\sigma _3/4}\begin{bmatrix} 1 &{} 0 \\ 0 &{} \pm \frac{2z\varkappa }{i\pi } \end{bmatrix}(\mathbf{1}+i\sigma _2)\left( \frac{\sqrt{2z}}{1+\sqrt{1-z^2}}\right) ^{\sigma _3}\begin{bmatrix} 1 &{} 0 \\ 0 &{} \frac{1}{2z} \end{bmatrix}\nonumber \\&\quad \times \left( \mathbf{1}+{\text {O}}\left( \frac{M^2}{\varkappa }\right) \right) 4^{a\sigma _3}e^{\pm \varkappa \left( \frac{1}{2}-g(z)\right) \sigma _3}, \end{aligned}$$

provided $\pm \mathfrak {I}\lambda \ge 0$, which is uniform for $z\in \tilde{\varOmega }_+$.

It remains to find the small $\lambda$ leading order asymptotics of the remaining factors of $\varGamma (z;\lambda )$. This is a tedious, but straightforward exercise.

#### Lemma 2

We have

158 $$\begin{aligned} \hat{\varGamma }^{-1}\left( \frac{1}{2}\right) =e^{-\frac{a\pi i}{2}\sigma _3}4^{-a\sigma _3}\begin{bmatrix} \frac{\varGamma \left( -\frac{a}{2}\right) \varGamma \left( -\frac{1}{2}-a\right) }{4\varGamma \left( \frac{1}{2}-\frac{a}{2}\right) \varGamma (-1-a)} &{} \quad \frac{i\varGamma \left( \frac{1}{2}-\frac{a}{2}\right) \varGamma \left( -\frac{1}{2}-a\right) }{8\varGamma \left( 1-\frac{a}{2}\right) \varGamma (-a)} \\ \frac{i\varGamma (\frac{a}{2}+\frac{1}{2})\varGamma (a+\frac{1}{2})}{\varGamma (\frac{a}{2}+1)\varGamma (a)} &{} \quad \frac{-\varGamma \left( \frac{a}{2}+1\right) \varGamma \left( a+\frac{1}{2}\right) }{\varGamma \left( \frac{a}{2}+\frac{1}{2}\right) \varGamma (a+2)} \end{bmatrix}. \end{aligned}$$

Moreover, in the limit $\lambda =e^{-\varkappa }\rightarrow 0$,

159 $$\begin{aligned} \hat{\varGamma }^{-1}\left( \frac{1}{2}\right) =\sqrt{\frac{2}{i}}e^{\mp \frac{\varkappa }{2}\sigma _3}4^{-a\sigma _3}\begin{bmatrix}\frac{1}{4} &{} \quad 0 \\ 0 &{} \quad 1 \end{bmatrix}\left( \mathbf{1}+i\sigma _2\right) \left( \mathbf{1}+{\text {O}}\left( \varkappa ^{-1}\right) \right) \begin{bmatrix} 1 &{} \quad 0 \\ 0 &{} \quad \pm \frac{\pi }{2\varkappa } \end{bmatrix}, \end{aligned}$$

provided $$\pm \mathfrak {I}\lambda \ge 0$$.

#### Proof

Using [1] 15.3.15 then 15.1.20, we have160where $(-1)^{-a/2}=e^{-a\pi i/2}$. Thus, in view of Theorem 1 and (12), we obtain the (1,1) entry of $\hat{\varGamma }\left( \frac{1}{2}\right)$. Repeating this process for $h_\infty ',s_\infty ,s_\infty '$, we obtain our explicit result. The asymptotics directly follow from the use of Stirling’s formula and Proposition 7. $\square$

According to Theorem 1,

161 $$\begin{aligned} Q(\lambda )=D(\mathbf{1}+ie^{a\pi i}\sigma _2),~~~\text { where}~~~ D:=\begin{bmatrix} -\tan (a\pi ) &{} \quad 0 \\ 0 &{} \quad 4^{2a+1}e^{a\pi i}\frac{\varGamma (a+3/2)\varGamma (a+1/2)}{\varGamma (a)\varGamma (a+2)} \end{bmatrix}. \end{aligned}$$

Combining that with Proposition 7 and 5.11.13 from [24], we obtain the following Lemma.

#### Lemma 3

In the limit $\lambda =e^{-\varkappa }\rightarrow 0$,

162 $$\begin{aligned} D=i\begin{bmatrix} \pm 1 &{}\quad 0 \\ 0 &{} \quad 4^{2a+1}e^{\pm \varkappa } \end{bmatrix}\left( \mathbf{1}+{\text {O}}\left( \varkappa ^{-1}\right) \right) , \end{aligned}$$

provided $$\pm \mathfrak {I}\lambda \ge 0$$.

We are ready to put the pieces from this section together and obtain the asymptotics of $\varGamma (z;\lambda )$ as $\lambda \rightarrow 0$ for $z\in \tilde{\varOmega }$. Define the matrix

163 $$\begin{aligned} \varPhi (z)&:=\frac{1}{2\sqrt{z}(z^2-1)^{1/4}}\begin{bmatrix} i+z+\sqrt{z^2-1} &{} -i-z+\sqrt{z^2-1} \\ i-z+\sqrt{z^2-1} &{} -i+z+\sqrt{z^2-1}\end{bmatrix}\nonumber \\&=\left( \mathbf{1}+\frac{i}{2z}(\sigma _3-i\sigma _2)\right) \left( \frac{z^2-1}{z^2}\right) ^{\sigma _1/4}. \end{aligned}$$

This matrix is a particular solution of the so-called model RHP 4 corresponding to $x=y=i/2$ in (188). As we will see in Theorem 15 below, the limit of $\varGamma (z;\lambda )$ as $\lambda \rightarrow 0$, $\mathfrak {I}\lambda >0$ distinguishes $\varPhi (z)$ among all other solutions of the model RHP.

#### Lemma 4

In the limit $\lambda =e^{-\varkappa }\rightarrow 0$

164 $$\begin{aligned}&D^{-1}\hat{\varGamma }^{-1}\left( \frac{1}{2}\right) \begin{bmatrix} 1 &{} \frac{-1}{2z(a+1)} \\ 0 &{} 1 \end{bmatrix}\hat{\varGamma }\left( \frac{z+1}{2z}\right) e^{\varkappa g(z)\sigma _3}\nonumber \\&\quad D= {\left\{ \begin{array}{ll} \varPhi (z)\left( \mathbf{1}+{\text {O}}\left( \frac{M^2}{\varkappa }\right) \right) , &{}\mathfrak {I}\lambda \ge 0 \\ \sigma _3\varPhi (z)\sigma _3\left( \mathbf{1}+{\text {O}}\left( \frac{M^2}{\varkappa }\right) \right) , &{}\mathfrak {I}\lambda \le 0 \end{array}\right. } \end{aligned}$$

uniformly in $$z\in \tilde{\varOmega }_+$$.

#### Proof

First take $\mathfrak {I}\lambda \ge 0$; the leading order term of $$D^{-1}\hat{\varGamma }^{-1}\left( \frac{1}{2}\right) \begin{bmatrix} 1 &{} \frac{-1}{2z(a+1)} \\ 0 &{} 1 \end{bmatrix}\hat{\varGamma }\left( \frac{z+1}{2z}\right) e^{\varkappa g\sigma _3}D$$ from Lemmas and Corollaries 3, 2, 3, we have

$$\begin{aligned}&\frac{\sqrt{2i}}{4}(\mathbf{1}+i\sigma _2)\begin{bmatrix} 1 &{} \quad -1 \\ 0 &{}\quad -iz \end{bmatrix}(1-z^2)^{\sigma _3/4}(\mathbf{1}+i\sigma _2)\left( \frac{\sqrt{2z}}{1+\sqrt{1-z^2}}\right) ^{\sigma _3}\begin{bmatrix} 1 &{} \quad 0 \\ 0 &{} \quad \frac{2}{z} \end{bmatrix} \\&\quad =\frac{(z^2-1)^{-1/4}}{2z^{3/2}}\left[ \left( \mathbf{1}-\sigma _1\right) +iz\left( \sigma _3-i\sigma _2\right) +\sqrt{1-z^2}\left( \sigma _3+i\sigma _2\right) \right] \\&\qquad \cdot \left[ \mathbf{1}-\sigma _3\sqrt{1-z^2}\right] =\varPhi (z). \end{aligned}$$

When $\mathfrak {I}\lambda \le 0$, observe that the leading order term of $$D^{-1}\hat{\varGamma }^{-1}\left( \frac{1}{2}\right) \begin{bmatrix} 1 &{} \frac{-1}{2z(a+1)} \\ 0 &{} 1 \end{bmatrix}\hat{\varGamma }\left( \frac{z+1}{2z}\right) e^{\varkappa g\sigma _3}D$$ is now

165 $$\begin{aligned} \sigma _3\cdot \frac{\sqrt{2i}}{4}(\mathbf{1}+i\sigma _2)\begin{bmatrix} 1 &{}\quad -1 \\ 0 &{} \quad -iz \end{bmatrix}(1-z^2)^{\sigma _3/4}(\mathbf{1}+i\sigma _2)\left( \frac{\sqrt{2z}}{1+\sqrt{1-z^2}}\right) ^{\sigma _3}\begin{bmatrix} 1 &{} \quad 0 \\ 0 &{} \quad \frac{2}{z} \end{bmatrix}\cdot \sigma _3 \end{aligned}$$

and thus we have the leading order term. Since $\varPhi (z)$ is uniformly bounded away from 0 when $z\in \tilde{\varOmega }$, we immediately obtain the lower order term. $\square$

Define matrix

166 $$\begin{aligned} \varPsi _0(z;\varkappa )={\left\{ \begin{array}{ll} \varPhi (z), &{}\text { for } \mathfrak {I}[\varkappa ]\le 0, \\ \sigma _1\varPhi (z)\sigma _1, &{}\text { for } \mathfrak {I}[\varkappa ]\ge 0. \end{array}\right. } \end{aligned}$$

Note that the matrix $\sigma _1\varPhi (z)\sigma _1$ is also a solution to RHP 4 with $x=y=-i/2$ in (188). Now we are ready to prove one of the main results of this section.

#### Theorem 15

**Fig. 1:**
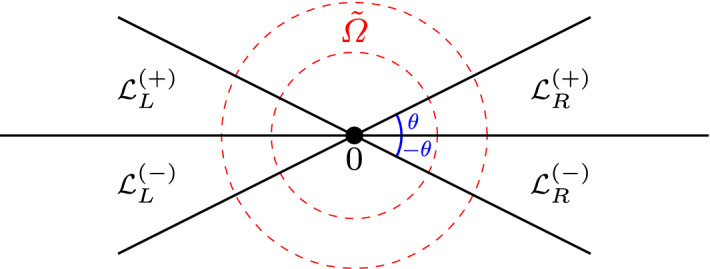
The set $\tilde{\varOmega }$ and lenses

Let $\theta \in (0,\pi /2)$ be fixed. Then

$$\begin{aligned} \varGamma (z;\lambda ) = {\left\{ \begin{array}{ll} \varPsi _0(z;\varkappa )\left( \mathbf{1}+{\text {O}}\left( \frac{M^2}{\varkappa }\right) \right) e^{-\varkappa g(z)\sigma _3}, ~ &{}z\in \tilde{\varOmega }, \theta<|\arg (z)|<\pi -\theta , \\ \varPsi _0(z;\varkappa )\left( \mathbf{1}+{\text {O}}\left( \frac{M^2}{\varkappa }\right) \right) \begin{bmatrix} 1 &{} 0 \\ \pm ie^{\varkappa (2g(z)-1)} &{} 1 \end{bmatrix}e^{-\varkappa g(z)\sigma _3}, &{}z\in \tilde{\varOmega }, ~ \pi -\theta<\arg (z)<\pi +\theta , \\ \varPsi _0(z;\varkappa )\left( \mathbf{1}+{\text {O}}\left( \frac{M^2}{\varkappa }\right) \right) \begin{bmatrix} 1 &{} \mp ie^{-\varkappa (2g(z)+1)} \\ 0 &{} 1 \end{bmatrix}e^{-\varkappa g(z)\sigma _3},&z\in \tilde{\varOmega }, ~ -\theta<\arg (z)<\theta \end{array}\right. } \end{aligned}$$

as $\lambda =e^{-\varkappa }\rightarrow 0$ uniformly in $z\in \tilde{\varOmega }$, provided $\pm \mathfrak {I}\lambda \ge 0$. See Fig. 1 for $\theta , \tilde{\varOmega }$.

#### Proof

First assume $\mathfrak {I}\lambda \ge 0$ and $z\in \tilde{\varOmega }_+$ (this implies $z\in \tilde{\varOmega }$ and $\mathfrak {I}z\le 0$). The following calculation

167 $$\begin{aligned} e^{-\varkappa g\sigma _3}Q=e^{-\varkappa g\sigma _3}D\left( \mathbf{1}+ie^{a\pi i}\sigma _2\right) e^{-\varkappa g\sigma _3}e^{\varkappa g\sigma _3}=D\left( e^{-2\varkappa g\sigma _3}+ie^{a\pi i}\sigma _2\right) e^{\varkappa g\sigma _3} \end{aligned}$$

and use of Lemma 4 give us

168 $$\begin{aligned} \varGamma (z;\lambda )&=\frac{\sigma _2}{1+e^{2a\pi i}}(\mathbf{1}-ie^{a\pi i}\sigma _2)D^{-1}\hat{\varGamma }^{-1}(\infty )\begin{bmatrix} 1 &{} \frac{-1}{2z(a+1)} \\ 0 &{} 1 \end{bmatrix}\hat{\varGamma }\nonumber \\&\quad \left( \frac{z+1}{2z}\right) e^{\varkappa g\sigma _3}D(\sigma _2e^{2\varkappa g\sigma _3}+ie^{a\pi i}I)e^{-\varkappa g\sigma _3} \nonumber \\&=\varPhi (z)\left( \mathbf{1}+{\text {O}}\left( \frac{M^2}{\varkappa }\right) \right) \begin{bmatrix} 1 &{} ie^{-\varkappa (2g+1)} \\ -ie^{\varkappa (2g-1)} &{} 1 \end{bmatrix}e^{-\varkappa g\sigma _3}, \end{aligned}$$

as desired. Now take $\mathfrak {I}\lambda \le 0$ and proceed similar to above. We use the calculation

169 $$\begin{aligned} e^{\varkappa g\sigma _3}Q=e^{\varkappa g\sigma _3}D(\mathbf{1}+ie^{a\pi i}\sigma _2)e^{-\varkappa g\sigma _3}e^{\varkappa g\sigma _3}=D(\mathbf{1}+ie^{a\pi i}e^{2\varkappa g\sigma _3}\sigma _2)e^{\varkappa g\sigma _3} \end{aligned}$$

and Lemma 4 to obtain

170 $$\begin{aligned} \varGamma (z;\lambda )=\sigma _1\varPhi (z)\sigma _1\left( \mathbf{1}+{\text {O}}\left( \frac{M^2}{\varkappa }\right) \right) \begin{bmatrix} 1 &{} ie^{-\varkappa (2g+1)} \\ -ie^{\varkappa (2g-1)} &{} 1 \end{bmatrix}e^{-\varkappa g\sigma _3}. \end{aligned}$$

The results for $z\in \tilde{\varOmega }$ with $\mathfrak {I}z\ge 0$ are immediate via use of the symmetry $\overline{\varGamma (\bar{z};\bar{\lambda })}=\varGamma (z;\lambda )$. Recall that ${\overline{\varPhi (\bar{z})}=\sigma _1\varPhi (z)\sigma _1}$ from Remark 13, $\overline{g(\bar{z})}=g(z)$ and $\varkappa (\lambda )=\overline{\varkappa (\bar{\lambda })}$. $\square$

### Deift–Zhou steepest descent method

The *g*-function, defined in (142), will play an important role so we list its relevant properties, all of which follow directly from Proposition 7.

#### Proposition 6

*g*(*z*) has the following properties:

1. *g*(*z*) is analytic on $\mathbb {\bar{C}}{\setminus } [-1,1]$, Schwarz symmetric and $g(\infty )=0$,
2. $$g_+(z)+g_-(z)=1$$ for $$z\in [-1,0]$$,   $$g_+(z)+g_-(z)=-1$$ for $$z\in [0,1]$$,
3. $\mathfrak {R}{(2g(z)-1)}=0$ for $z\in [-1,0]$ and $\mathfrak {R}{(2g(z)-1)}<0$ for $z\in \overline{\mathbb {C}}{\setminus }[-1,0]$,
4. $\mathfrak {R}{(2g(z)+1)}=0$ for $z\in [0,1]$ and $\mathfrak {R}{(2g(z)+1)}>0$ for $z\in \overline{\mathbb {C}}{\setminus }[0,1]$,

#### Transformation $\varGamma (z;\lambda )\rightarrow Z(z;\varkappa )$

Our first transformation will be

171 $$\begin{aligned} Y(z;\varkappa ):=\varGamma (z;e^{-\varkappa })e^{\varkappa g(z)\sigma _3}, \end{aligned}$$

where $\varGamma (z;\lambda )$ was defined in (17). Since $\varGamma (z;\lambda )$ is the solution of RHP 1, it is easy to show that $Y(z;\varkappa )$ solves the following RHP.

##### Riemann–Hilbert Problem 2

Find a matrix $Y(z;\varkappa )$, $e^{-\varkappa }=\lambda \in \mathbb {C}{\setminus }\{0\}$, analytic for $z\in \bar{\mathbb {C}}{\setminus }[-1,1]$ and satisfying the following conditions:

172 $$\begin{aligned} Y(z_+;\varkappa )=&Y(z_-;\varkappa )\begin{bmatrix}e^{\varkappa (g_+-g_-)} &{} -ie^{-\varkappa (g_++g_--1)} \\ 0 &{} e^{-\varkappa (g_+-g_-)}\end{bmatrix}, ~ z\in (-1,0) \end{aligned}$$

173 $$\begin{aligned} Y(z_+;\varkappa )=&Y(z_-;\varkappa )\begin{bmatrix}e^{\varkappa (g_+-g_-)} &{} 0 \\ ie^{\varkappa (g_++g_-+1)} &{} e^{-\varkappa (g_+-g_-)}\end{bmatrix}, ~ z\in (0,1) \end{aligned}$$

174 $$\begin{aligned} Y(z;\varkappa ) =&1+{\text {O}}\left( z^{-1}\right) \text { as } z\rightarrow \infty , \end{aligned}$$

175 $$\begin{aligned} Y(z;\varkappa )=&\begin{bmatrix} {\text {O}}\left( 1\right)&{\text {O}}\left( \log (z+1)\right) \end{bmatrix} \text { as } z\rightarrow -1, \end{aligned}$$

176 $$\begin{aligned} Y(z;\varkappa )=&\begin{bmatrix} {\text {O}}\left( \log (z-1)\right)&{\text {O}}\left( 1\right) \end{bmatrix} \text { as } z\rightarrow 1, \end{aligned}$$

177 $$\begin{aligned} Y(z;\varkappa )\in&L^2_{loc} \text { as } z\rightarrow 0. \end{aligned}$$

The endpoint behavior is listed column-wise.

The jumps for $Y(z;\varkappa )$ on $(-1,0)$ and (0, 1) can be written as

178 $$\begin{aligned} Y(z_+;\varkappa )&=Y(z_-;\varkappa )\begin{bmatrix} 1 &{} \quad 0 \\ ie^{\varkappa (2g_-(z)-1)} &{} \quad 1 \end{bmatrix}(-i\sigma _1)\begin{bmatrix} 1 &{} \quad 0 \\ ie^{\varkappa (2g_+(z)-1)} &{} \quad 1 \end{bmatrix}, \quad z\in (-1,0) \end{aligned}$$

179 $$\begin{aligned} Y(z_+;\varkappa )&=Y(z_-;\varkappa )\begin{bmatrix} 1 &{}\quad \frac{1}{i}e^{-\varkappa (2g_-(z)+1)} \\ 0 &{} \quad 1 \end{bmatrix}(i\sigma _1)\begin{bmatrix} 1 &{}\quad \frac{1}{i}e^{-\varkappa (2g_+(z)+1)} \\ 0 &{} \quad 1 \end{bmatrix}, \quad z\in (0,1). \end{aligned}$$

**Fig. 2:**
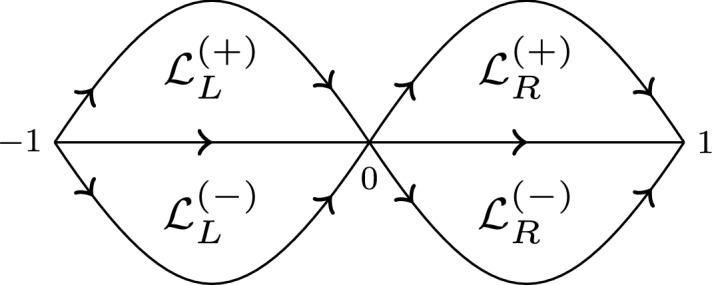
Lense regions $$\mathcal {L}_{L,R}^{(\pm )}$$

This decomposition can be verified by direct matrix multiplication and by using the jump properties of *g*(*z*) in Proposition 6. We define the ‘lense’ regions $\mathcal {L}_{L,R}^{(\pm )}$ as in Fig. 2.

Recall, from Proposition 6, that $\mathfrak {R}[2g(z)+1]\ge 0$ with equality only for $z\in (0,1)$ and $\mathfrak {R}[2g(z)-1]\le 0$ with equality only for $z\in (-1,0)$. Our second and final transformation is

180 $$\begin{aligned} Z(z;\varkappa ) := {\left\{ \begin{array}{ll} Y(z;\varkappa ), &{} \quad \text {{ z} outside the lenses} \\ Y(z;\varkappa )\begin{bmatrix} 1 &{}\quad 0 \\ \mp ie^{\varkappa (2g(z)-1)} &{}\quad 1 \end{bmatrix}, &{} \quad z\in \mathcal {L}_L^{(\pm )} \\ Y(z;\varkappa )\begin{bmatrix} 1 &{}\quad \mp \frac{1}{i}e^{-\varkappa (2g(z)+1)} \\ 0 &{} \quad 1 \end{bmatrix},&\quad z\in \mathcal {L}_R^{(\pm )}. \end{array}\right. } \end{aligned}$$

Since $Y(z;\varkappa )$ solves RHP 2, it is a direct calculation to show $Z(z;\varkappa )$ solves the following RHP.

##### Riemann–Hilbert Problem 3

Find a matrix $Z(z;\varkappa )$, analytic on the complement of the arcs of Fig. 2, satisfying the jump conditions

181 $$\begin{aligned} Z(z_+;\varkappa )=Z(z_-;\varkappa ) {\left\{ \begin{array}{ll} \begin{bmatrix} 1 &{} 0 \\ ie^{\varkappa (2g-1)} &{} 1 \end{bmatrix} &{} z\in \partial \mathcal {L}_L^{(\pm )}{\setminus }\mathbb {R}, \\ \begin{bmatrix} 1 &{} \frac{1}{i}e^{-\varkappa (2g+1)} \\ 0 &{} 1 \end{bmatrix} &{} z\in \partial \mathcal {L}_R^{(\pm )}{\setminus }\mathbb {R}, \\ -i\sigma _1 &{} z\in (-1,0), \\ i\sigma _1 &{} z\in (0,1), \end{array}\right. } \end{aligned}$$

normalized by

182 $$\begin{aligned} Z(z;\varkappa )=1+{\text {O}}\left( z^{-1}\right) , \text { as } z\rightarrow \infty , \end{aligned}$$

and with the same endpoint behavior as $Y(z;\varkappa )$ near the endpoints $z=0,\pm 1$, see (175).

The jumps for $Z(z;\varkappa )$ on $\partial \mathcal {L}_{L,R}^{(\pm )}$ will be exponentially small as long as *z* is a fixed distance away from $0,\pm 1$ due to Proposition 6. If we ‘ignore’ the jumps on the lenses of the RHP for $Z(z;\varkappa )$, we obtain the so-called model RHP.

##### Riemann–Hilbert Problem 4

Find a matrix $\varPsi (z)$, analytic on $\overline{\mathbb {C}}{\setminus } [-1,1]$, and satisfying

183 $$\begin{aligned} \varPsi _+(z)&=\varPsi _-(z)(-i\sigma _1), \text { for } z\in [-1,0], \end{aligned}$$

184 $$\begin{aligned} \varPsi _+(z)&=\varPsi _-(z)(i\sigma _1), \text { for } z\in [0,1], \end{aligned}$$

185 $$\begin{aligned} \varPsi (z)&={\text {O}}\left( |z\mp 1|^{-\frac{1}{4}}\right) , \text { as } z\rightarrow \pm 1, \end{aligned}$$

186 $$\begin{aligned} \varPsi (z)&={\text {O}}\left( |z|^{-\frac{1}{2}}\right) , \text { as } z\rightarrow 0, \end{aligned}$$

187 $$\begin{aligned} \varPsi (z)&=\mathbf{1}+{\text {O}}\left( z^{-1}\right) \text { as } z\rightarrow \infty . \end{aligned}$$

Note that condition (186) does not guarantee the uniqueness of $\varPsi (z)$.

##### Theorem 16

$\varPsi (z)$ is a solution to RHP 4 if and only if there exist $x,y\in \mathbb {C}$ such that

188 $$\begin{aligned} \varPsi (z)=\left( \mathbf{1}+\frac{1}{z}\begin{bmatrix} x &{} -x \\ y &{} -y \end{bmatrix}\right) \left( \frac{z^2-1}{z^2}\right) ^{\sigma _1/4}. \end{aligned}$$

##### Proof

The Sokhotski–Plemelj formula (see [14]) can be applied to this problem to obtain the solution

189 $$\begin{aligned} \varPsi _1(z)=\beta (z)^{\sigma _1}, ~ \text { where } ~ \beta (z)=\left( \frac{z^2-1}{z^2}\right) ^{1/4}. \end{aligned}$$

Take any solution to RHP 4 (different from $\varPsi _1(z)$) and call it $\varPsi _2(z)$. Then it can be seen that the matrix $\varPsi _2(z)\varPsi _1^{-1}(z)$ has no jumps in the complex plane, $\varPsi _2(z)\varPsi _1^{-1}(z)=\mathbf{1}+{\text {O}}\left( z^{-1}\right)$ as $z\rightarrow \infty$ and $\varPsi _2(z)\varPsi _1^{-1}(z)={\text {O}}\left( z^{-1}\right)$ as $z\rightarrow 0$. Then it must be that

190 $$\begin{aligned} \varPsi _2(z)\varPsi _1^{-1}(z)=\mathbf{1}+\frac{A}{z}, \end{aligned}$$

where *A* is a constant matrix. Notice that

191 $$\begin{aligned} \varPsi _1(z)=\frac{\beta (z)}{2}\begin{bmatrix} 1 &{} \quad 1 \\ 1 &{} \quad 1 \end{bmatrix}+\frac{1}{2\beta (z)}\begin{bmatrix} 1 &{} \quad -1 \\ -1 &{} \quad 1 \end{bmatrix}, \end{aligned}$$

so we have

192 $$\begin{aligned} \varPsi _2(z)=\left( \mathbf{1}+\frac{A}{z}\right) \varPsi _1(z)=\left( \mathbf{1}+\frac{A}{z}\right) \left( \frac{\beta (z)}{2}\begin{bmatrix} 1 &{} \quad 1 \\ 1 &{} \quad 1 \end{bmatrix}+\frac{1}{2\beta (z)}\begin{bmatrix} 1 &{}\quad -1 \\ -1 &{} \quad 1 \end{bmatrix}\right) . \end{aligned}$$

Since $\varPsi _2(z)$ is a solution of RHP 4, it must be true that $\varPsi _2(z)={\text {O}}\left( z^{-1/2}\right)$ as $z\rightarrow 0$. Thus the matrix *A* must satisfy

193 $$\begin{aligned} A\cdot \begin{bmatrix} 1 &{} \quad 1 \\ 1 &{} \quad 1 \end{bmatrix}=\begin{bmatrix} 0 &{} \quad 0 \\ 0 &{} \quad 0 \end{bmatrix} ~ \implies ~ A=\begin{bmatrix} x &{}\quad -x \\ y &{} \quad -y \end{bmatrix}. \end{aligned}$$

It is easy now to check that (188) with any $x,y\in \mathbb {C}$ satisfies RHP 4. $\square$

##### Remark 13

Assume $\varPsi (z)$ is a solution of the RHP 4. Then $\det \varPsi (z)\equiv 1$ if and only if $y=x$ in the representation (188). If, additionally, $x\in i\mathbb {R}$ in this representation then $\varPsi (z)$ has the symmetry

194 $$\begin{aligned} \overline{\varPsi (\overline{z})}=\sigma _1\varPsi (z)\sigma _1. \end{aligned}$$

Both properties can be easily verified.

#### Approximation of $Z(z;\varkappa )$ and main result

We will construct a piecewise (in *z*) approximation of $Z(z;\varkappa )$ when $\varkappa \rightarrow \infty$. Our approach is very similar to that in [6]. Denote by $\mathbb {D}_j$ a disc of small radius *l* centered at *j*, $j=0,\pm 1$, where *l* is chosen so that $\partial \mathbb {D}_0\subset \tilde{\varOmega }$. The idea is as follows: on the lenses $\mathcal {L}_{L,R}^{(\pm )}$ (see Fig. 2) outside the discs $\mathbb {D}_j$, $j=0,\pm 1$, the jumps of $Z(z;\varkappa )$ are uniformly close to the identity matrix thus $\varPsi _0(z;\varkappa )$ (a solution to model RHP, see (166)) is a ‘good’ approximation of $Z(z;\varkappa )$. Inside $\mathbb {D}_j$, $j=0,\pm 1$, we construct local approximations that are commonly called ‘parametrices’. The solution of the so-called Bessel RHP is necessary.

##### Riemann–Hilbert Problem 5

Let $\nu \in (0,\pi )$ be any fixed number. Find a matrix $\mathcal {B}_\nu (\zeta )$ that is analytic off the rays $\mathbb {R}_-$, $e^{\pm i\theta }\mathbb {R}^+$ and satisfies the following conditions.

195 $$\begin{aligned} \mathcal {B}_{\nu +}(\zeta )&=\mathcal {B}_{\nu -}(\zeta )\begin{bmatrix} 1 &{} \quad 0 \\ e^{-4\sqrt{\zeta }\pm i\pi \nu } &{} \quad 1 \end{bmatrix}, \quad \zeta \in e^{\pm i\theta }\mathbb {R}_+ \end{aligned}$$

196 $$\begin{aligned} \mathcal {B}_{\nu +}(\zeta )&=\mathcal {B}_{\nu -}(\zeta )\begin{bmatrix} 0 &{} \quad 1 \\ -1 &{} \quad 0 \end{bmatrix}, \quad \zeta \in \mathbb {R}_- \end{aligned}$$

197 $$\begin{aligned} \mathcal {B}_{\nu }(\zeta )&={\text {O}}\left( \zeta ^{-\frac{|\nu |}{2}}\right) \text { for } \nu \ne 0 \text { or } {\text {O}}\left( \log {\zeta }\right) \text { for } \nu =0 \text { as } \zeta \rightarrow 0, \end{aligned}$$

198 $$\begin{aligned} \mathcal {B}_{\nu }(\zeta )&=F(\zeta )\left( 1+{\text {O}}\left( \frac{1}{\sqrt{\zeta }}\right) \right) \text { as } \zeta \rightarrow \infty , \nonumber \\&\quad \text {where}~~ F(\zeta ):=(2\pi )^{-\sigma _3/2}\zeta ^{-\frac{\sigma _3}{4}}\frac{1}{\sqrt{2}}\begin{bmatrix} 1 &{} \quad -i \\ -i &{}\quad 1 \end{bmatrix}. \end{aligned}$$

This RHP has an explicit solution in terms of Bessel functions and can be found in [31]. Define local coordinates at points $z=\pm 1$ as

199 $$\begin{aligned} -4\sqrt{\xi _{-1}(z)}=&\varkappa (2g(z)-1), ~ \text { for }z\in \mathbb {D}_{-1}, \end{aligned}$$

200 $$\begin{aligned} 4\sqrt{\xi _1(z)}=&\varkappa (2g(z)+1), ~ \text { for }z\in \mathbb {D}_1. \end{aligned}$$

We call $\tilde{Z}(z;\varkappa )$ our approximation of $Z(z;\varkappa )$ and define

201 $$\begin{aligned} \tilde{Z}(z;\varkappa ):= {\left\{ \begin{array}{ll} \varPsi _0(z;\varkappa ), &{} \quad z \in \mathbb {C}{\setminus } \displaystyle {\bigcup _{j=-1}^1}\mathbb {D}_j, \\ \varPsi _0(z;\varkappa )i^{-\frac{\sigma _3}{2}}F^{-1}(\xi _{-1})\mathcal {B}_0(\xi _{-1})i^{\frac{\sigma _3}{2}}, &{} \quad z \in \mathbb {D}_{-1}, \\ Z(z;\varkappa ), &{} \quad z \in \mathbb {D}_0, \\ \varPsi _0(z;\varkappa )i^{-\frac{\sigma _3}{2}}\sigma _1F^{-1}(\xi _1)\mathcal {B}_0(\xi _1)\sigma _1i^{\frac{\sigma _3}{2}}, &{} \quad z \in \mathbb {D}_1. \end{array}\right. } \end{aligned}$$

##### Remark 14

The matrix $\tilde{Z}(z;\varkappa )$ was constructed to have exactly the same jumps as $Z(z;\varkappa )$ when $z\in \mathbb {D}_{0,\pm 1}\cup [-1,1]$. For more details see [6], section 4.3.

##### Corollary 4

202 $$\begin{aligned} Z(z;\varkappa )=\varPsi _0(z;\varkappa )\left( \mathbf{1}+{\text {O}}\left( \frac{M^2}{\varkappa }\right) \right) ~~~\text {as}~~~\varkappa \rightarrow \infty \end{aligned}$$

uniformly in $$z\in \tilde{\varOmega }$$,

##### Proof

In Theorem 15, we obtained the leading order behavior of $\varGamma (z;\lambda )$ for $z\in \tilde{\varOmega }$ as $\lambda \rightarrow 0$. This Theorem can easily be written in terms of $Z(z;\varkappa )$ instead of $\varGamma (z;\lambda )$ by applying the transformations (see Sect. 5.3.1) $\varGamma \rightarrow Y\rightarrow Z$. $\square$

Define the error matrix as

203 $$\begin{aligned} \mathcal {E}(z;\varkappa ):=Z(z;\varkappa )\tilde{Z}^{-1}(z;\varkappa ), \end{aligned}$$

It is clear that $\mathcal {E}(z;\varkappa )=\mathbf{1}+{\text {O}}\left( z^{-1}\right)$ as $z\rightarrow \infty$ since both $Z(z;\varkappa ),\tilde{Z}(z;\varkappa )$ have this behavior. $\mathcal {E}(z;\varkappa )$ has no jumps inside $\mathbb {D}_{-1,0,1}$ because $\tilde{Z}(z;\varkappa )$ was constructed to have the same jumps as $Z(z;\varkappa )$ inside $\mathbb {D}_{-1,0,1}$, see Remark 14. Thus $\mathcal {E}(z;\varkappa )$ will have jumps on $\partial \mathbb {D}_{-1,0,1}$, $\partial \mathcal {L}_{L,R}^{(\pm )}{\setminus }\mathbb {D}_{0,\pm 1}$, and be analytic elsewhere. Explicitly,

204 $$\begin{aligned} \mathcal {E}(z_+;\varkappa )=\mathcal {E}(z_-;\varkappa ) {\left\{ \begin{array}{ll} \varPsi _0(z;\varkappa )i^{-\frac{\sigma _3}{2}}F^{-1}(\xi _{-1})B_0(\xi _{-1})i^{\frac{\sigma _3}{2}}\varPsi _0^{-1}(z;\varkappa ), &{}z\in \partial \mathbb {D}_{-1}, \\ \mathbf{1}+\varPsi _0(z;\varkappa )\begin{bmatrix} 0 &{} 0 \\ ie^{\varkappa (2g(z)-1)} &{} 0 \end{bmatrix}\varPsi _0^{-1}(z;\varkappa ), &{}z\in \partial \mathcal {L}_L^{(\pm )}{\setminus }\mathbb {D}_{-1,0}, \\ Z(z;\varkappa )\varPsi _0^{-1}(z;\varkappa ), &{}z\in \partial \mathbb {D}_0, \\ \mathbf{1}+\varPsi _0(z;\varkappa )\begin{bmatrix} 0 &{} -ie^{-\varkappa (2g(z)+1)} \\ 0 &{} 0 \end{bmatrix}\varPsi _0^{-1}(z;\varkappa ), &{}z\in \partial \mathcal {L}_R^{(\pm )}{\setminus }\mathbb {D}_{0,1}, \\ \varPsi _0(z;\varkappa )i^{-\frac{\sigma _3}{2}}\sigma _1F^{-1}(\xi _{1})B_0(\xi _{1})\sigma _1i^{\frac{\sigma _3}{2}}\varPsi _0^{-1}(z;\varkappa ), &{}z\in \partial \mathbb {D}_{1}. \end{array}\right. } \end{aligned}$$

**Fig. 3:**
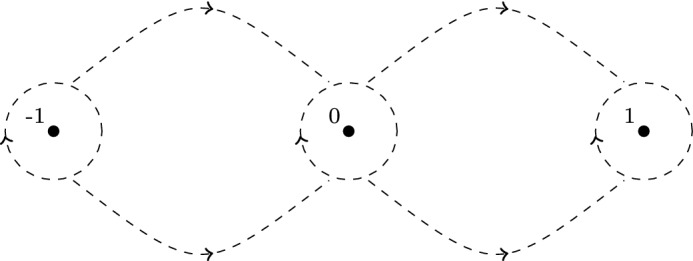
The contour $\varSigma$, where $\mathcal {E}(z;\varkappa )$ has jumps

Call $\varSigma$ the collection of arcs where $\mathcal {E}(z;\varkappa )$ has a jump, as described in Fig. 3.

##### Remark 15

The factors $e^{-\varkappa (2g(z)+1)}$ and $e^{\varkappa (2g(z)-1)}$ in (205) are exponentially small for all *z* in the corresponding set, in light of Proposition 6.

Now revisiting the jumps of $\mathcal {E}(z;\varkappa )$ in (204), we have another Corollary.

##### Corollary 5

For any $$z\in \varSigma $$,

205 $$\begin{aligned} \mathcal {E}(z_+;\varkappa )=\mathcal {E}(z_-;\varkappa ) {\left\{ \begin{array}{ll} \mathbf{1}+{\text {O}}\left( \frac{M^2}{\varkappa }\right) , &{}z\in \partial \mathbb {D}_{-1,0,1}, \\ \mathbf{1}+{\text {O}}\left( e^{\varkappa (2g(z)-1)}\right) , &{}z\in \partial \mathcal {L}_L^{(\pm )}{\setminus }\mathbb {D}_{-1,0}, \\ \mathbf{1}+{\text {O}}\left( e^{-\varkappa (2g(z)+1)}\right) , &{}z\in \partial \mathcal {L}_R^{(\pm )}{\setminus }\mathbb {D}_{0,1}, \end{array}\right. }~~~\text {as}~~~\varkappa \rightarrow \infty \end{aligned}$$

uniformly in $$z\in \varSigma $$.

##### Proof

The behavior for $z\in \partial \mathbb {D}_{\pm 1},\partial \mathbb {D}_{0}$ is a direct consequence of (198), Corollary 4, respectively. The behavior on the lenses is clear via inspection of (204). $\square$

##### Corollary 6

Let *J* be a compact subset of $\mathbb {C}{\setminus }\{-1,0,1\}$, where we distinguish the points on the upper and lower sides of $(-1,0)$ and (0, 1). Then we have the approximation

206 $$\begin{aligned} Z(z;\varkappa )=\varPsi _0(z;\varkappa )\left( \mathbf{1}+{\text {O}}\left( \frac{M^2}{\varkappa }\right) \right) ~~~\text {as}~~\varkappa \rightarrow \infty \end{aligned}$$

uniformly in $$z\in J$$.

##### Proof

Given *J*, the disks $\mathbb {D}_{0,\pm 1}$ can be taken sufficiently small in order to not intersect *J*. Corollaries 4, 5 and the so-called small norm theorem, see [9] Theorem 7.171, can now be applied to conclude that $\mathcal {E}(z;\varkappa )=\mathbf{1}+{\text {O}}\left( \frac{M^2}{\varkappa }\right)$ uniformly for $z\in J$. This is equivalent to the stated result. $\square$

We are now ready to prove the main result of this section.

##### Theorem 17

In the limit $\lambda =e^{-\varkappa }\rightarrow 0$:

1. For *z* in compact subsets of $\mathbb {C}{\setminus }[-1,1]$ we have the uniform approximation
  207 $$\begin{aligned} \varGamma (z;\lambda ) = \varPsi _0(z;\varkappa )\left( \mathbf{1}+{\text {O}}\left( \frac{M^2}{\varkappa }\right) \right) e^{-\varkappa g(z)\sigma _3}; \end{aligned}$$
2. For *z* in compact subsets of $(-1,0)\cup (0,1)$ we have the uniform approximation
  208 $$\begin{aligned} \varGamma (z_\pm ;\lambda ) = {\left\{ \begin{array}{ll} \varPsi _0(z_\pm ;\varkappa )\left( \mathbf{1}+{\text {O}}\left( \frac{M^2}{\varkappa }\right) \right) \begin{bmatrix} 1 &{} 0 \\ \pm ie^{\varkappa (2g(z_\pm )-1)} &{} 1 \end{bmatrix}e^{-\varkappa g(z_\pm )\sigma _3}, &{} z\in (-1,0), \\ \varPsi _0(z_\pm ;\varkappa )\left( \mathbf{1}+{\text {O}}\left( \frac{M^2}{\varkappa }\right) \right) \begin{bmatrix} 1 &{} \mp ie^{-\varkappa (2g(z_\pm )+1)} \\ 0 &{} 1 \end{bmatrix}e^{-\varkappa g(z_\pm )\sigma _3},&z\in (0,1), \end{array}\right. } \end{aligned}$$

where ± denotes the upper/lower shore of the real axis in the *z*-plane.

##### Proof

This Theorem is a direct consequence of Corollary 6. We simply need to revert the transforms that took $\varGamma \rightarrow Z$. Doing so, we find that

209 $$\begin{aligned} \varGamma (z;\lambda )={\left\{ \begin{array}{ll} Z(z;\varkappa )e^{-\varkappa g(z)\sigma _3}, &{} \quad \text {{ z} outside lenses} \\ Z(z;\varkappa )\begin{bmatrix} 1 &{} \quad 0 \\ \pm ie^{\varkappa (2g(z)-1)} &{}\quad 1 \end{bmatrix} e^{-\varkappa g(z)\sigma _3}, &{}\quad z\in \mathcal {L}_L^{(\pm )} \\ Z(z;\varkappa )\begin{bmatrix} 1 &{}\quad \mp ie^{-\varkappa (2g(z)+1)} \\ 0 &{}\quad 1 \end{bmatrix} e^{-\varkappa g(z)\sigma _3},&\quad z\in \mathcal {L}_R^{(\pm )}. \end{array}\right. } \end{aligned}$$

Since *z* is in a compact subset of $\mathbb {C}{\setminus }[-1,1]$ or $(-1,0)\cup (0,1)$, we apply Corollary 6 to obtain the result. $\square$

## Figures and Tables

**Fig. 4:**
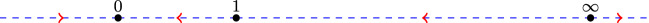
Orientation of the real axis of the $\eta$-plane

**Fig. 5:**
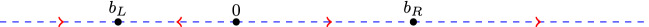
Orientation of the real axis of the *z*-plane

**Fig. 6:**
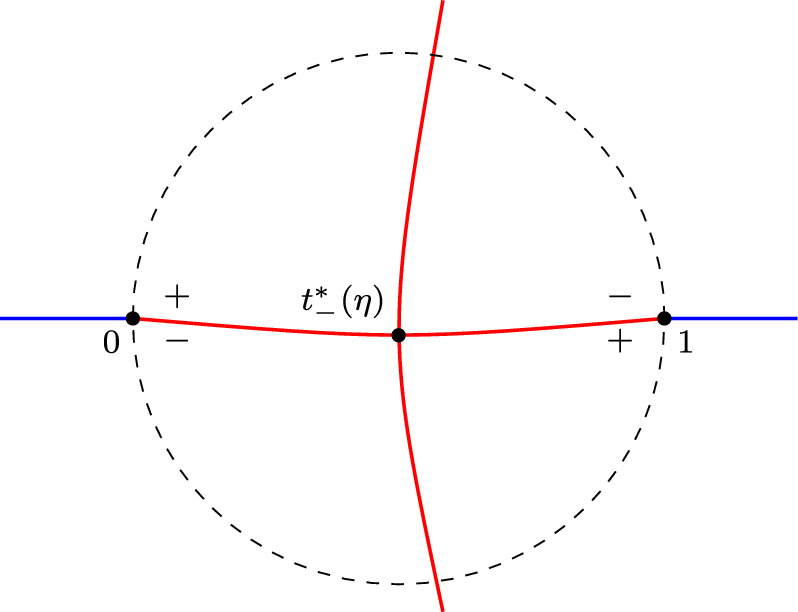
This is a visualization of Lemma 5. The blue lines are the branch cuts of $S_\eta (t)$, the red curves form the level set $\mathfrak {R}\left[ S_\eta (t)-S_\eta (t^*_-(\eta ))\right] =0$ and the black dashed circle has center and radius 1/2. The $+$ denotes regions where $\mathfrak {R}\left[ S_\eta (t)-S_\eta (t^*_-(\eta ))\right] >0$ and − denotes regions where $\mathfrak {R}\left[ S_\eta (t)-S_\eta (t^*_-(\eta ))\right] <0$

**Fig. 7:**
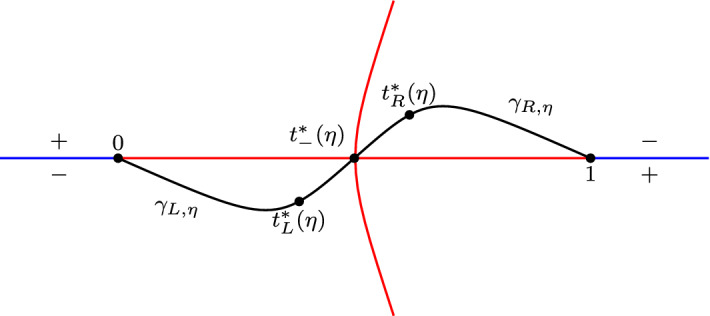
$$\gamma _\eta =\gamma _{L,\eta }+\gamma _{C,\eta }+\gamma _{R,\eta }$$

**Fig. 8:**
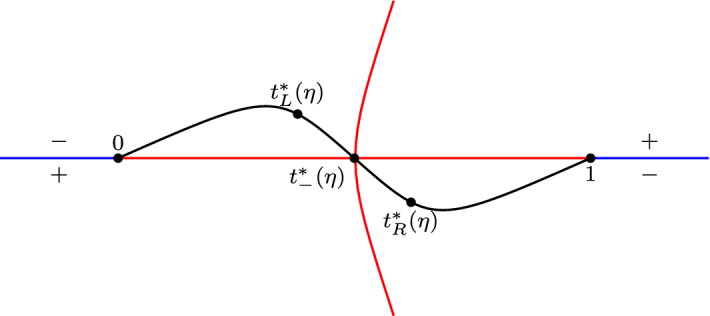
Path of integration when $\lambda$ is in the lower half plane

## Appendix A: Construction of $\varGamma (z;\lambda )$

Recall that the Hypergeometric ODE (see [24] 15.10.1) is

210 $$\begin{aligned} \eta (1-\eta )\frac{{\mathrm {d}}^{2}w}{{\mathrm {d}\eta }^{2}}+\left( c-(a+b+1)\eta \right) \frac{\mathrm {d}w}{\mathrm {d}\eta }-abw=0, \end{aligned}$$

which has exactly three regular singular points at $\eta =0,1,\infty$. The idea is to choose parameters *a*, *b*, *c* so that the monodromy matrices of the fundamental matrix solution solution of the ODE will match (up to similarity transformation) the jump matrices of RHP 1. We orient the real axis of the $\eta -$plane as described in Fig. 4.

### A.1 Solutions of ODE (210) near regular singular points and connection formula

According to [24] 15.10.11–15.10.16, three pairs of linearly independent solutions of ODE (210) when $\eta =0,1,\infty$, respectively, are

211

212

213

From [24] 15.10.7, we have that

214 $$\begin{aligned} W_\eta \left[ h_\infty (\eta ),s_\infty (\eta )\right] =e^{(a+b)\pi i}(a-b)\eta ^{-c}(1-\eta )^{c-a-b-1}. \end{aligned}$$

Kummer’s 20 connection formula are listed in [24] 15.10.17–15.10.36. We will list only what is necessary in this construction. The connection between solutions at $\eta =0$ and $\eta =\infty$ is (see [24] 15.10.19, 15.10.20, 15.10.25, 15.10.26)

215 $$\begin{aligned} \begin{bmatrix} h_0(\eta )&\quad s_0(\eta ) \end{bmatrix}&=\begin{bmatrix} h_\infty (\eta )&\quad s_\infty (\eta ) \end{bmatrix}C_{\infty 0}, \end{aligned}$$

216 $$\begin{aligned} \begin{bmatrix} h_\infty (\eta )&\quad s_\infty (\eta ) \end{bmatrix}&=\begin{bmatrix} h_0(\eta )&\quad s_0(\eta ) \end{bmatrix}C_{0 \infty }, \end{aligned}$$

where

217 $$\begin{aligned} C_{\infty 0}&=\begin{bmatrix} \frac{\varGamma \left( c\right) \varGamma \left( b-a\right) }{\varGamma \left( b\right) \varGamma \left( c-a\right) } &{} \quad e^{(1-c)\pi i}\frac{\varGamma \left( 2-c\right) \varGamma \left( b-a\right) }{\varGamma \left( 1-a\right) \varGamma \left( b-c+1\right) } \\ \frac{\varGamma \left( c\right) \varGamma \left( a-b\right) }{\varGamma \left( a\right) \varGamma \left( c-b\right) } &{} \quad e^{(1-c)\pi i}\frac{\varGamma \left( 2-c\right) \varGamma \left( a-b\right) }{\varGamma \left( 1-b\right) \varGamma \left( a-c+1\right) } \end{bmatrix}, \end{aligned}$$

218 $$\begin{aligned} C_{\infty 0}^{-1}&=C_{0\infty }=\begin{bmatrix} \frac{\varGamma \left( 1-c\right) \varGamma \left( a-b+1\right) }{\varGamma \left( a-c+1\right) \varGamma \left( 1-b\right) } &{}\quad \frac{\varGamma \left( 1-c\right) \varGamma \left( b-a+1\right) }{\varGamma \left( b-c+1\right) \varGamma \left( 1-a\right) } \\ e^{(c-1)\pi i}\frac{\varGamma \left( c-1\right) \varGamma \left( a-b+1\right) }{\varGamma \left( a\right) \varGamma \left( c-b\right) } &{} \quad e^{(c-1)\pi i}\frac{\varGamma \left( c-1\right) \varGamma \left( b-a+1\right) }{\varGamma \left( b\right) \varGamma \left( c-a\right) } \end{bmatrix}. \end{aligned}$$

The connection between solutions at $\eta =1$ and $\eta =\infty$ is (see [24] 15.10.23, 15.10.24, 15.10.27, 15.10.28)

219 $$\begin{aligned} \begin{bmatrix} h_1(\eta )&\quad s_1(\eta ) \end{bmatrix}&=\begin{bmatrix} h_\infty (\eta )&\quad s_\infty (\eta ) \end{bmatrix}C_{\infty 1}, \end{aligned}$$

220 $$\begin{aligned} \begin{bmatrix} h_\infty (\eta )&\quad s_\infty (\eta ) \end{bmatrix}&=\begin{bmatrix} h_1(\eta )&\quad s_1(\eta ) \end{bmatrix}C_{1\infty }, \end{aligned}$$

where

221 $$\begin{aligned} C_{\infty 1}&=\begin{bmatrix} e^{-a\pi i}\frac{\varGamma \left( a+b-c+1\right) \varGamma \left( b-a\right) }{\varGamma \left( b\right) \varGamma \left( b-c+1\right) } &{} \quad e^{(b-c)\pi i}\frac{\varGamma \left( c-a-b+1\right) \varGamma \left( b-a\right) }{\varGamma \left( 1-a\right) \varGamma \left( c-a\right) } \\ e^{-b\pi i}\frac{\varGamma \left( a+b-c+1\right) \varGamma \left( a-b\right) }{\varGamma \left( a\right) \varGamma \left( a-c+1\right) } &{} \quad e^{(a-c)\pi i}\frac{\varGamma \left( c-a-b+1\right) \varGamma \left( a-b\right) }{\varGamma \left( 1-b\right) \varGamma \left( c-b\right) } \end{bmatrix}, \end{aligned}$$

222 $$\begin{aligned} C_{\infty 1}^{-1}&=C_{1\infty }=\begin{bmatrix} e^{a\pi i}\frac{\varGamma \left( a-b+1\right) \varGamma \left( c-a-b\right) }{\varGamma \left( 1-b\right) \varGamma \left( c-b\right) } &{} \quad e^{b\pi i}\frac{\varGamma \left( b-a+1\right) \varGamma \left( c-a-b\right) }{\varGamma \left( 1-a\right) \varGamma \left( c-a\right) } \\ e^{(c-b)\pi i}\frac{\varGamma \left( a-b+1\right) \varGamma \left( a+b-c\right) }{\varGamma \left( a\right) \varGamma \left( a-c+1\right) } &{} \quad e^{(c-a)\pi i}\frac{\varGamma \left( b-a+1\right) \varGamma \left( a+b-c\right) }{\varGamma \left( b\right) \varGamma \left( b-c+1\right) } \end{bmatrix}. \end{aligned}$$

### A.2 Selection of parameters *a*, *b*, *c*

Define

223 $$\begin{aligned} \hat{\varGamma }(\eta ):=\eta ^{\frac{c}{2}}(1-\eta )^{\frac{a+b-c+1}{2}}\begin{bmatrix} h_\infty (\eta ) &{} \quad s_\infty (\eta ) \\ h'_\infty (\eta ) &{} \quad s'_\infty (\eta ) \end{bmatrix}. \end{aligned}$$

Notice that for any $\eta \in \mathbb {C}$

224 $$\begin{aligned} \det \left( \hat{\varGamma }(\eta )\right) =e^{(a+b)\pi i}(a-b) \end{aligned}$$

according to (214). Our solution $\varGamma (z;\lambda )$ to RHP 1 has singular points at $z=b_L,0,b_R$. Notice that the Möbius transform

225 $$\begin{aligned} \eta =M_1(z):=\frac{b_R(z-b_L)}{z(b_R-b_L)} \end{aligned}$$

maps $b_L\rightarrow 0$, $b_R\rightarrow 1$, and $0\rightarrow \infty$ where the orientation of the $z-$axis is described in Fig. 5. Thus we are interested in the matrix

226 $$\begin{aligned} \hat{\varGamma }\left( M_1(z)\right) =\left( \frac{b_R(z-b_L)}{|b_L|(z-b_R)}\right) ^{\frac{c}{2}}\left( \frac{|b_L|(z-b_R)}{z(b_R-b_L)}\right) ^{\frac{a+b+1}{2}}\begin{bmatrix} h_\infty \left( M_1(z)\right) &{} s_\infty \left( M_1(z)\right) \\ h_\infty '\left( M_1(z)\right) &{} s_\infty '\left( M_1(z)\right) \end{bmatrix}. \end{aligned}$$

We need to determine parameters *a*, *b*, *c* such that $\hat{\varGamma }(M_1(z))$ is $L^2_{\text {loc}}$ at $z=b_L,0,b_R$, so we are interested in the bi-resonant case, which is

227 $$\begin{aligned} c\in \mathbb {Z}, \qquad c-b-a\in \mathbb {Z}. \end{aligned}$$

When $z= b_L$, to guarantee that $\hat{\varGamma }$ is $L^2_{loc}$ we must have that (use the connection formula of section A.1 to easily inspect the local behavior)

228 $$\begin{aligned} \frac{c}{2}>-\frac{1}{2}, ~~~ -\frac{c}{2}>-\frac{1}{2}. \end{aligned}$$

Since $c\in \mathbb {Z}$, it must be so that $c=0$. Now for $z=b_R$, we must have that

229 $$\begin{aligned} \frac{a+b-c+1}{2}&=\frac{r+1}{2}>-\frac{1}{2}, \end{aligned}$$

230 $$\begin{aligned} \frac{1-a-b}{2}&=\frac{-r-1}{2}>-\frac{1}{2} \end{aligned}$$

where $a+b=r\in \mathbb {Z}$. Since $r\in \mathbb {Z}$, the only possibility is $r=-1$. So we have that $b=-1-a$ and $c=0$. Lastly, as $z\rightarrow 0$,

231 $$\begin{aligned} h_\infty \left( M_1(z)\right)&={\text {O}}\left( z^a\right) , ~~~ h_\infty '\left( M_1(z)\right) ={\text {O}}\left( z^{a+1}\right) , \end{aligned}$$

232 $$\begin{aligned} s_\infty \left( M_1(z)\right)&=-e^{-a\pi i}\left( \frac{-b_Rb_L}{z(b_R-b_L)}\right) ^{a+1}+{\text {O}}\left( z^{-a}\right) , \end{aligned}$$

233 $$\begin{aligned} s_\infty '\left( M_1(z)\right)&=-(a+1)e^{-a\pi i}\left( \frac{-b_Rb_L}{z(b_R-b_L)}\right) ^{a}+{\text {O}}\left( z^{-a+1}\right) , \end{aligned}$$

so we see that it is not possible for $\hat{\varGamma }(M_1(z))$ to have $L^2$ behavior at $z=0$. On the other hand, observe that

234 $$\begin{aligned} s_\infty \left( M_1(z)\right) +\frac{b_Lb_R}{z(b_R-b_L)(a+1)}s_\infty '\left( M_1(z)\right) ={\text {O}}\left( z^{-a}\right) , ~~ z\rightarrow 0. \end{aligned}$$

Thus the matrix

235 $$\begin{aligned} \begin{bmatrix} 1 &{} \frac{b_Lb_R}{z(b_R-b_L)(a+1)} \\ 0 &{} 1 \end{bmatrix}\hat{\varGamma }(M_1(z)) \end{aligned}$$

is $L^2_{\text {loc}}$ as $z\rightarrow 0$ provided that $|\mathfrak {R}(a)|<1/2$. In the next section, we solve for *a* explicitly in terms of $\lambda$ and the condition $|\mathfrak {R}(a)|<1/2$ will be met provided that $\lambda \notin [-1/2,1/2]$, see Appendix B.

### A.3 Monodromy

The monodromy matrices of $\hat{\varGamma }(z)$ about the singular points $z=0,1,\infty$ are

236 $$\begin{aligned} M_0=C_{\infty 0}e^{i\pi c\sigma _3}C_{0\infty },\qquad M_1=C_{\infty 1}e^{i\pi (a+b-c+1)\sigma _3}C_{1\infty },\qquad M_\infty =e^{i\pi (b-a-1)\sigma _3}, \end{aligned}$$

where $C_{\infty 0}, C_{0 \infty }, C_{\infty 1}, C_{1 \infty }$ are defined in (217), (218), (221), (222), respectively. With some effort it can be shown that

237 $$\begin{aligned} M_0&=\begin{bmatrix} \cos \pi c\left( 1-2i\frac{\sin \pi a\sin \pi b}{\sin \pi (b-a)}\right) &{} \quad \frac{2\pi i\varGamma (b-a)\varGamma (b-a+1)}{\varGamma (b)\varGamma (c-a)\varGamma (b-c+1)\varGamma (1-a)} \\ \frac{2\pi i\varGamma (a-b)\varGamma (a-b+1)}{\varGamma (a)\varGamma (c-b)\varGamma (a-c+1)\varGamma (1-b)} &{} \quad \cos \pi c\left( 1+2i\frac{\sin \pi a\sin \pi b}{\sin \pi (b-a)}\right) \end{bmatrix}\nonumber \\&\quad +\sin \pi c\frac{\sin \pi (a+b)}{\sin \pi (b-a)}\begin{bmatrix} 1 &{} \quad 0 \\ 0 &{} \quad 1 \end{bmatrix}, \end{aligned}$$

238 $$\begin{aligned} M_1&=e^{\frac{i\pi }{2}(b-a)\sigma _3}M_0\big |_{c\rightarrow a+b-c+1}e^{-\frac{i\pi }{2}(b-a)\sigma _3}. \end{aligned}$$

From the previous section, we take $c=0$ and $b=-1-a$. It is important to note that the connection matrices $C_{\infty 0}, C_{0 \infty }, C_{\infty 1}, C_{1 \infty }$ are singular when $c=0$ and/or $b=-1-a$, but we can see that $M_0, M_1$ are not. Taking $c=0$ and $b=-a-1$, we obtain

239 $$\begin{aligned} M_0&=\begin{bmatrix} 1-i\tan (a\pi ) &{}\quad \frac{\tan ^2(a\pi )\varGamma (a)\varGamma (a+2)}{i4^{2a+1}\varGamma (a+\frac{1}{2})\varGamma (a+\frac{3}{2})} \\ \frac{i4^{2a+1}\varGamma (a+\frac{1}{2})\varGamma (a+\frac{3}{2})}{\varGamma (a)\varGamma (a+2)} &{} \quad 1+i\tan (a\pi ) \end{bmatrix}, \end{aligned}$$

240 $$\begin{aligned} M_1&=\sigma _3e^{-i\pi a\sigma _3}M_0e^{i\pi a\sigma _3}\sigma _3, \end{aligned}$$

241 $$\begin{aligned} M_\infty&=e^{-2\pi ia\sigma _3}. \end{aligned}$$

Let

242 $$\begin{aligned} Q(\lambda )=\begin{bmatrix} -\tan (a\pi ) &{} \quad 0 \\ 0 &{} \quad 4^{2a+1}e^{a\pi i}\frac{\varGamma (a+3/2)\varGamma (a+1/2)}{\varGamma (a)\varGamma (a+2)} \end{bmatrix}\begin{bmatrix} 1 &{} \quad e^{a\pi i} \\ -e^{a\pi i} &{} \quad 1 \end{bmatrix} \end{aligned}$$

so then we have

243 $$\begin{aligned} Q^{-1}M_0Q&=\begin{bmatrix} 1 &{} \quad 0 \\ -\frac{e^{2\pi ia}-1}{e^{a\pi i}} &{} \quad 1 \end{bmatrix}, ~~~ Q^{-1}M_1Q=\begin{bmatrix} 1 &{}\quad -\frac{e^{2\pi ia}-1}{e^{a\pi i}} \\ 0 &{}\quad 1 \end{bmatrix}, \end{aligned}$$

244 $$\begin{aligned} Q^{-1}M_\infty Q&=\begin{bmatrix} \frac{e^{4\pi ia}-e^{2\pi ia}+1}{e^{2\pi ia}} &{} \quad \frac{1-e^{2\pi ia}}{e^{a\pi i}} \\ \frac{1-e^{2\pi ia}}{e^{a\pi i}} &{} \quad 1 \end{bmatrix} \end{aligned}$$

The match requires

245 $$\begin{aligned} \frac{e^{2\pi ia}-1}{e^{a\pi i}}=\frac{i}{\lambda } \end{aligned}$$

which implies

246 $$\begin{aligned} a(\lambda )=\frac{1}{i\pi }\ln \left( \frac{i+\sqrt{4\lambda ^2-1}}{2\lambda }\right) . \end{aligned}$$

In Appendix B we have listed all the important properties of $a(\lambda )$.

### A.4 Proof of Theorem 1

We will now construct $\varGamma (z;\lambda )$ so that it is the solution of RHP 1.

#### Proof

Notice that the matrix

247 $$\begin{aligned} \begin{bmatrix} 1 &{} \quad \frac{b_Lb_R}{z(b_R-b_L)(a+1)} \\ 0 &{} \quad 1 \end{bmatrix}\hat{\varGamma }(M_1(z))Q(\lambda )\sigma _2, \end{aligned}$$

where $\hat{\varGamma }, M_1, a$ are defined in (223), (225), (246), respectively, satisfies the following properties:

- $L^2$ behavior at $z=0$, provided $\lambda \not \in [-1/2,1/2]$, due to (235) and properties of $a(\lambda )$ (see Appendix B),
- jump matrix $$\begin{bmatrix} 1 &{} -\frac{i}{\lambda } \\ 0 &{} 1 \end{bmatrix}$$ for $z\in (b_L,0)$ with positive orientation, see (243) and Fig. 5 for orientation,
- jump matrix $$\begin{bmatrix} 1 &{} 0 \\ \frac{i}{\lambda } &{} 1 \end{bmatrix}$$ for $z\in (0,b_R)$ with positive orientation, see (243),
- column-wise behavior $$\begin{bmatrix} {\text {O}}\left( 1\right)&{\text {O}}\left( \ln (z-b_L)\right) \end{bmatrix}$$ as $z\rightarrow b_L$, because the first column has no jump on $(b_L,0)$ and is analytic for $z\not \in [b_L,b_R]$ so the first column is ${\text {O}}\left( 1\right)$ as $z\rightarrow b_L$. The Sokhotski–Plemelj formula can be used to inspect the behavior of the second column.
- column-wise behavior $$\begin{bmatrix} {\text {O}}\left( \ln (z-b_R)\right)&{\text {O}}\left( 1\right) \end{bmatrix}$$ as $z\rightarrow b_R$, same idea as above,
- behavior $$\hat{\varGamma }(M_1(\infty ))Q(\lambda )\sigma _2(\mathbf{1}+{\text {O}}\left( z^{-1}\right) )$$ as $$z\rightarrow \infty $$,
- analytic for $z\in \mathbb {C}{\setminus }[b_L,b_R]$, due to properties of hypergeometric functions.

Thus, we conclude that the matrix

248 $$\begin{aligned} \varGamma (z;\lambda ):=\sigma _2Q^{-1}(\lambda )\hat{\varGamma }^{-1}(M_1(\infty ))\begin{bmatrix} 1 &{} \frac{b_Lb_R}{z(b_R-b_L)(a+1)} \\ 0 &{} 1 \end{bmatrix}\hat{\varGamma }(M_1(z))Q(\lambda )\sigma _2 \end{aligned}$$

is a solution of RHP 1 provided that $\lambda \notin [-1/2,1/2]$. Uniqueness follows immediately from the $L^2$ behavior of $\varGamma (z;\lambda )$ at the endpoints $z=b_L,0,b_R$. $\square$

## Appendix B: Definition and properties of $a(\lambda )$

Recall, from (15), that

249 $$\begin{aligned} a(\lambda )=\frac{1}{i\pi }\ln \left( \frac{i+\sqrt{4\lambda ^2-1}}{2\lambda }\right) \end{aligned}$$

where $\sqrt{4\lambda ^2-1}=2\lambda +{\text {O}}\left( 1\right)$ as $\lambda \rightarrow \infty$ and the principle branch of the logarithm is taken. The following proposition lists all relevant properties of $a(\lambda )$, none of which are difficult to prove.

### Proposition 7

The function $a(\lambda )$ has the following properties:

1. $a(\lambda )$ is analytic and Schwarz symmetric for $\lambda \in \mathbb {C}{\setminus }[-1/2,1/2]$.
2. $$a_+(\lambda )+a_-(\lambda )={\left\{ \begin{array}{ll} 1, &{}\lambda \in (0,1/2) \\ -1, &{}\lambda \in (-1/2,0)\end{array}\right. }$$
3. $$\mathfrak {R}[a_\pm (\lambda )]={\left\{ \begin{array}{ll} \frac{1}{2}, &{}\lambda \in (0,1/2) \\ -\frac{1}{2}, &{}\lambda \in (-1/2,0) \end{array}\right. }$$ and $$-\frac{1}{2}<\mathfrak {R}\left[ a(\lambda )\right] <\frac{1}{2}$$ for $$\lambda \in \overline{\mathbb {C}}{\setminus }\left[ -\frac{1}{2},\frac{1}{2}\right] $$.
4. For $$\lambda \in (-1/2,1/2)$$, $$\mathfrak {I}[a_+(\lambda )]<0$$, $$\mathfrak {I}[a_-(\lambda )]>0$$, and $$\mathfrak {I}[a_+(\lambda )]=-\mathfrak {I}[a_-(\lambda )]$$.
5. If $\lambda \rightarrow 0$, provided that either $\mathfrak {I}\lambda \ge 0$ or $\mathfrak {I}\lambda \le 0$, then
  250 $$\begin{aligned} a(\lambda )={\left\{ \begin{array}{ll} -\frac{1}{i\pi }\ln (\lambda )+\frac{1}{2}+{\text {O}}\left( \lambda ^2\right) =\frac{\varkappa }{i\pi }+\frac{1}{2}+{\text {O}}\left( e^{-2\varkappa }\right) , &{}\mathfrak {I}\lambda \ge 0, \\ \frac{1}{i\pi }\ln (\lambda )+\frac{1}{2}+{\text {O}}\left( \lambda ^2\right) =-\frac{\varkappa }{i\pi }+\frac{1}{2}+{\text {O}}\left( e^{-2\varkappa }\right) , &{}\mathfrak {I}\lambda \le 0, \end{array}\right. } \end{aligned}$$
  where $$\varkappa =-\ln \lambda $$.
6. As $\lambda \rightarrow 0$, provided that either $\mathfrak {I}\lambda \ge 0$ or $\mathfrak {I}\lambda \le 0$, then
  251 $$\begin{aligned} e^{i\pi a(\lambda )}={\left\{ \begin{array}{ll} ie^{\varkappa }(1+{\text {O}}\left( e^{-2\varkappa }\right) ), &{}\mathfrak {I}\lambda \ge 0, \\ ie^{-\varkappa }(1+{\text {O}}\left( e^{-2\varkappa }\right) ), &{}\mathfrak {I}\lambda \le 0. \end{array}\right. } \end{aligned}$$

## Appendix C: Properties of $d_L(z;\lambda ),d_R(z;\lambda )$

In this Appendix we compute and list the important properties of the functions $d_L(z;\lambda )$ and $d_R(z;\lambda )$. Recall that [from Eq. (37)]

252 $$\begin{aligned} d_R(z;\lambda )&:=\alpha (\lambda )h_\infty '\left( \frac{b_R(z-b_L)}{z(b_R-b_L)}\right) +\beta (\lambda )s_\infty '\left( \frac{b_R(z-b_L)}{z(b_R-b_L)}\right) , \end{aligned}$$

253 $$\begin{aligned} d_L(z;\lambda )&:=-e^{a\pi i}\alpha (\lambda )h_\infty '\left( \frac{b_R(z-b_L)}{z(b_R-b_L)}\right) +e^{-a\pi i}\beta (\lambda )s_\infty '\left( \frac{b_R(z-b_L)}{z(b_R-b_L)}\right) , \end{aligned}$$

where

254 $$\begin{aligned} \alpha (\lambda ):=\frac{e^{-a\pi i}\tan (a\pi )\varGamma (a)}{4^{a+1}\varGamma (a+3/2)}, ~~ \beta (\lambda ):=\frac{4^{a}e^{a\pi i}\varGamma (a+1/2)}{\varGamma (a+2)} \end{aligned}$$

and $h_\infty ',s_\infty '$ are defined in (12), (13).

### Proposition 8

For $$\lambda \in (-1/2,0)$$,

1. $$d_R\left( M_2(x);\lambda \right) =d_L(x;\lambda )$$, where $$M_2(x)=\frac{b_Rb_Lx}{x(b_R+b_L)-b_Rb_L}$$,
2. $d_L(z;\lambda )$ and $d_R(z;\lambda )$ are single-valued in $\lambda$,
3. $d_L(z;\lambda )$ is analytic for $z\in \overline{\mathbb {C}}{\setminus }[0,b_R]$, $\overline{d_L(z;\lambda )}=d_L(\overline{z};\overline{\lambda })$ for $z\in \overline{\mathbb {C}}$, and
  255 $$\begin{aligned} d_L(z_+;\lambda )-d_L(z_-;\lambda )=\frac{i}{\lambda }d_R(z;\lambda ), ~~~ \text {for } z\in (0,b_R), \end{aligned}$$
4. $d_R(z;\lambda )$ is analytic for $z\in \overline{\mathbb {C}}{\setminus }[b_L,0]$, $\overline{d_R(z;\lambda )}=d_R(\overline{z};\overline{\lambda })$ for $z\in \overline{\mathbb {C}}$, and
  256 $$\begin{aligned} d_R(z_+;\lambda )-d_R(z_-;\lambda )=-\frac{i}{\lambda }d_L(z;\lambda ), ~~~ \text {for } z\in (b_L,0), \end{aligned}$$
5. We have the SVD system
  257 $$\begin{aligned} \mathcal {H}_R\left[ \frac{d_R(y;\lambda )}{y}\right] (x)=2\lambda \frac{d_L(x;\lambda )}{x}, ~~~ \mathcal {H}_L\left[ \frac{d_L(x;\lambda )}{x}\right] (y)=2\lambda \frac{d_R(y;\lambda )}{y} \end{aligned}$$
  where $$x\in (b_L,0)$$ and $$y\in (0,b_R)$$.
6. $$\displaystyle {\int _0^{b_R}\frac{d_R(x;\lambda )}{x}~dx=2\pi \lambda d_L(\infty ;\lambda )}$$, $$\displaystyle {\int _{b_L}^0\frac{d_L(x;\lambda )}{x}~dx=-2\pi \lambda d_R(\infty ;\lambda )}$$

We obtain the corresponding identities for $D_L(z;\lambda ), D_R(z;\lambda )$ when $\lambda \in (-1,1)$ via the relation $D_R(z;\lambda ):=d_R(z;-|\lambda |/2)$ and $D_L(z;\lambda ):=d_L(z;-|\lambda |/2)$.

### Proof

1. Recall that $M_j(x)$, $j=1,2,3$, are defined in Remark 4. It is easy to show that $1-M_3(x)=M_1(x)$, $1-\frac{1}{M_3(x)}=\frac{-M_1(x)}{M_3(x)}$. From [1] 15.5.7, 15.5.13, (taking $a:=a_-(\lambda )$ and $-1=e^{-i\pi }$) we obtain
  258
  thus we have
  259
  Taking $a\rightarrow -a-1$ in the identity above yields
  260 $$\begin{aligned} s_\infty '(M_3(x))=e^{-a\pi i}s_\infty '(M_1(x)), \end{aligned}$$
  because $h_\infty (\eta )|_{a\rightarrow -a-1}=s_\infty (\eta )$. Now using the definition of $d_R(x;\lambda )$, (259), (260), and that $M_1(M_2(x))=M_3(x)$, we obtain the result.
2. Notice that $h_\infty (z)\big |_{a\rightarrow -a-1}=s_\infty (z)$ so we have $h_\infty '(z,\lambda _+)=s_\infty '(z,\lambda _-)$. To compute the jumps of the coefficients $\alpha ,\beta$, we use [24] 5.5.3 to obtain $\alpha _+(\lambda )=\beta _-(\lambda )$ and, similarly, $\beta _+(\lambda )=\alpha _-(\lambda )$. This can now be used to prove the statement.
3. Recall, from the proof of Theorem 4, that $d_L(z;\lambda ), d_R(z;\lambda )$ was defined in terms of the (2, 1), (2, 2) element, respectively, of the matrix
  261 $$\begin{aligned} M(z,\lambda )=\hat{\varGamma }(M_1(z),\lambda )Q(\lambda )\sigma _2. \end{aligned}$$
  Using the definition of $\varGamma (z;\lambda )$, see (17), some simple algebra shows that
  262 $$\begin{aligned} M(z,\lambda )=\begin{bmatrix} 1 &{} \frac{-b_Lb_R}{z(b_R-b_L)(a+1)} \\ 0 &{} 1 \end{bmatrix}\hat{\varGamma }^{-1}(M_1(\infty ))Q\sigma _2\varGamma (z;\lambda ). \end{aligned}$$
  Since $\varGamma (z;\lambda )$ is a solution of RHP 1, we know $M(z,\lambda )$ is analytic for $z\in \overline{\mathbb {C}}{\setminus }[b_L,b_R]$ and
  263 $$\begin{aligned} M_+=M_-\begin{bmatrix} 1 &{} \quad -\frac{i}{\lambda } \\ 0 &{}\quad 1 \end{bmatrix},\quad z\in (b_L,0), \quad M_+=M_-\begin{bmatrix} 1 &{} \quad 0 \\ \frac{i}{\lambda } &{} \quad 1 \end{bmatrix},\quad z\in (0,b_R), \end{aligned}$$
  which immediately gives the jumps and analyticity of both $d_L,d_R$. For the symmetry, notice that, by definition,
  264
  and $a=a(\lambda ), M_1(z)$ are Schwarz symmetric. Thus $\overline{d_R(z;\lambda )}=d_R(\overline{z};\overline{\lambda })$ and the symmetry of $d_L(z;\lambda )$ follows from the relation $d_R(M_2(x);\lambda )=d_L(x;\lambda )$.
4. This was proven above.
5. Let $\gamma _R$ be the circle with center and radius of $b_R/2$ with negative orientation. Then,
  265 $$\begin{aligned} \mathcal {H}_R\left[ \frac{d_R(y;\lambda )}{y}\right] (x)&=\frac{2\lambda }{2\pi i}\int _0^{b_R}\frac{\frac{i}{\lambda }d_R(y;\lambda )}{y(y-x)}~dy=\frac{2\lambda }{2\pi i}\int _0^{b_R}\frac{\varDelta _yd_L(y;\lambda )}{y(y-x)}~dy \nonumber \\&=\frac{2\lambda }{2\pi i}\int _{\gamma _R}\frac{d_L(y;\lambda )}{y(y-x)}~dy=2\lambda d_L(x;\lambda ) \end{aligned}$$
  where we have deformed $\gamma _R$ through $z=\infty$ and into the circle of center and radius $b_L/2$ with positive orientation and then apply residue theorem. The remaining computation is nearly identical.
6. The idea is similar to that of the last proof;
  266 $$\begin{aligned} \int _0^{b_R}\frac{d_R(x;\lambda )}{x}~dx&=\frac{2\pi \lambda }{2\pi i}\int _0^{b_R}\frac{\frac{i}{\lambda }d_R(x;\lambda )}{x}~dx=\frac{2\pi \lambda }{2\pi i}\int _{\gamma _R}\frac{d_L(x;\lambda )}{x}~dx\nonumber \\&=2\pi \lambda d_L(\infty ;\lambda ), \end{aligned}$$
  and the remaining identity is proven analogously.

$$\square $$

## Appendix D: Proof of Theorem 14

### D.1 Deformation of [0, 1] to $\gamma _\eta$

For any $\eta \in \varOmega _+$, we want the path $\gamma _\eta$ to have the property

267 $$\begin{aligned} \mathfrak {R}\left[ S_\eta (t)-S_\eta (t^*_-(\eta ))\right] \le 0 \end{aligned}$$

for all $t\in \gamma _\eta$ with equality only when $t=t_-^*(\eta )$, so it is important to understand the collection of the level curves of

268 $$\begin{aligned} \mathfrak {R}\left[ S_\eta (t)-S_\eta (t^*_-(\eta ))\right] =0, \end{aligned}$$

which obviously depends on $\eta$.

#### Lemma 5

For each $\eta \in \varOmega _+$ we have the following:

1. There is exactly one curve $l_0$ emitted from $t=0$. Moreover, $l_0$ lies in the sector $|\arg (t)|\le \pi /4$ for sufficiently small |*t*|.
2. There is exactly one curve $l_1$ emitted from $t=1$. Moreover, $l_0$ lies in the sector $|\arg (1-t)|\le \pi /4$ for sufficiently small $|1-t|$.
3. There exists exactly one $\theta =\theta _{u,\eta }\in (0,\pi )$ such that $\mathfrak {R}\left[ S_\eta (1/2+1/2e^{i\theta _{u,\eta }})\right] =\mathfrak {R}\left[ S_\eta (t^*_-(\eta ))\right]$. Moreover, for *M* sufficiently large, $\theta _{u,\eta }\in (\pi /4,3\pi /4)$.
4. There exists exactly one $\theta =\theta _{l,\eta }\in (-\pi ,0)$ such that $\mathfrak {R}\left[ S_\eta (1/2+1/2e^{i\theta _{l,\eta }})\right] =\left[ S_\eta (t^*_-(\eta ))\right]$. Moreover, for *M* sufficiently large, $\theta _{l,\eta }\in (-3\pi /4,-\pi /4)$.

#### Proof

For brevity, define

269 $$\begin{aligned} \alpha _\eta (t)&:=\mathfrak {R}\left[ S_\eta (t)-S_\eta (t^*_-(\eta ))\right] =\arg \left( \frac{\eta t(1-t)}{\eta -t}\right) -2\arg \left( t^*_-(\eta )\right) . \end{aligned}$$

The statements listed above are equivalent to

1. For any $r>0$ sufficiently small, $\alpha _\eta (re^{i\theta })$ is increasing for $-\pi<\theta <\pi$ and ${\exists !\theta _{0,\eta ,r}\in (-\pi /4,\pi /4)}$ such that ${\alpha _\eta (re^{i\theta _{0,\eta ,r}})=0}$,
2. For any $r>0$ sufficiently small, $\alpha _\eta (1-re^{i\theta })$ is increasing for $-\pi<\theta <\pi$ and ${\exists !\theta _{1,\eta ,r}\in (-\pi /4,\pi /4)}$ such that ${\alpha _\eta (1-re^{i\theta _{1,\eta ,r}})=0}$,
3. $\alpha _\eta \left( \frac{1}{2}+\frac{1}{2}e^{i\theta }\right)$ is increasing for $0<\theta <\pi$ and $\exists !\theta _{u,\eta }\in (\pi /4,3\pi /4)$ such that ${\alpha _\eta \left( \frac{1}{2}+\frac{1}{2}e^{i\theta _{u,\eta }}\right) =0}$,
4. $\alpha _\eta \left( \frac{1}{2}+\frac{1}{2}e^{i\theta }\right)$ is increasing for $-\pi<\theta <0$ and $\exists !\theta _{l,\eta }\in (-3\pi /4,-\pi /4)$ such that ${\alpha _\eta \left( \frac{1}{2}+\frac{1}{2}e^{i\theta _{l,\eta }}\right) =0}$.

The proofs of each of the four claims above are similar so we prove the first.

270 $$\begin{aligned} \alpha _\eta (re^{i\theta })&=\arg (re^{i\theta })+\arg (1-re^{i\theta })-\arg (\eta -re^{i\theta })+\arg (\eta )-2\arg \left( t^*_-(\eta )\right) \equiv \theta \nonumber \\&\quad +\tan ^{-1}\left( \frac{r\sin \theta }{r\cos \theta -1}\right) -\tan ^{-1}\left( \frac{r\sin \theta -\mathfrak {I}\eta }{r\cos \theta -\mathfrak {R}\eta }\right) \nonumber \\&\quad +\arg (\eta )-2\arg \left( t^*_-(\eta )\right) \pmod {\pi } \end{aligned}$$

Differentiating with respect to $\theta$, we have

271 $$\begin{aligned} \frac{d}{d\theta }\left[ \alpha _\eta (re^{i\theta })\right]&=1+\frac{r^2-r\cos \theta }{(r\cos \theta -1)^2+r^2\sin ^2\theta }\nonumber \\&\quad -\frac{r^2-r(\mathfrak {R}(\eta )\cos \theta +\mathfrak {I}(\eta )\sin \theta )}{(r\cos \theta -\mathfrak {R}\eta )^2+(r\sin \theta -\mathfrak {I}\eta )^2}\rightarrow 1 \end{aligned}$$

as $r\rightarrow 0$. So with *r* small enough, $\frac{d}{d\theta }\left[ \alpha _\eta (re^{i\theta })\right] >0$ for any $\theta$ and for any $\eta \in \varOmega _+$. Take *M* sufficiently large so that

272 $$\begin{aligned} |\arg \left( t^*_-(\eta )\right) |<\pi /8 \end{aligned}$$

for all $\eta \in \varOmega _+$. Thus when *r* is sufficiently small,

273 $$\begin{aligned} \alpha _\eta (re^{i\pi /4})=\frac{\pi }{4}+\tan ^{-1}\left( \frac{r/\sqrt{2}}{r/\sqrt{2}-1}\right) -\arg \left( 1-\frac{re^{i\pi /4}}{\eta }\right) -2\arg \left( t^*_-(\eta )\right) >0 \end{aligned}$$

and similarly it can be shown that $\alpha _\eta (re^{-i\pi /4})<0$. Intermediate Value Theorem can now be applied to show $\exists !\theta _{0,\eta ,r}\in (-\pi /4,\pi /4)$ such that $\alpha _\eta (re^{i\theta _{0,\eta ,r}})=0$. $\square$

#### Remark 16

According to Lemma 5, for every $\eta \in \varOmega _+$, the set $t\in B(1/2,1/2)$ is split into 4 sectors by the level curve $\mathfrak {R}\left[ S_\eta (t)-S_\eta (t^*_-(\eta ))\right] =0$, see Fig. 6.

We have now proven that we can deform [0, 1] to an ‘appropriate’ path $\gamma _\eta$, as described in the following Theorem.

#### Theorem 18

For each $\eta \in \varOmega _+$, the path [0, 1] can be continuously deformed to a path $\gamma _\eta$ which begins at $t=0$ (with $\mathrm{arg}(t)<-\pi /4$ for small |*t*|), passes through $t=t_-^*(\eta )$, ends at $t=1$ (with $\mathrm{arg}(t-1)<3\pi /4$ for small $|t-1|$), and ${\gamma _\eta \subset B(1/2,1/2)}$. Moreover, $\mathfrak {R}\left[ S_\eta (t)-S_\eta (t^*_-(\eta ))\right] \le 0$ for all $t\in \gamma _\eta$ with equality only when $t=t_-^*(\eta )$. See (135), (132) for $\varOmega _+, S_\eta (t)$, respectively.

#### Proof

Let us fix some $\eta \in \varOmega _+$. The function $\mathfrak {R}S_\eta (t)$ is a harmonic function in $t\in B(1/2,1/2)$, so the level curves $\mathfrak {R}S_\eta (t)=\mathfrak {R}S_\eta (t^*_-(\eta ))$ can not form closed loops there. Since, according to Lemma 5, there exactly four level curves $\mathfrak {R}S_\eta (t)=\mathfrak {R}S_\eta (t^*_-(\eta ))$ entering the disc *B*(1/2, 1/2), and exactly four legs of this level curve emanating from $t=t^*_-(\eta )$, the only possible topology of these level curves is shown on Fig. 6. We can now deform [0, 1] to an ‘appropriate’ path $\gamma _\eta$ as stated in the theorem. The new path of integration, $\gamma _\eta$, is the black curve in Fig. 7. $\square$

### D.2 Estimates

Here we will split $\gamma _\eta$ into 3 pieces (see Fig. 7) and show that the leading order contribution in Theorem 14 comes from the piece containing $t^*_-(\eta )$. Define radius

274 $$\begin{aligned} r:=r_M=\max _{\eta \in \varOmega _+}\left| \frac{1}{2}-t^*_-(\eta )\right| =O(M^{-1}) \end{aligned}$$

so that $t^*_-(\eta )\in \overline{B}(1/2,r)$ for all $\eta \in \varOmega _+$. The latter estimate follows from Prposition 5. Also define function

275 $$\begin{aligned} v_\eta (t):=v\left( t,\eta \right) =\sqrt{S_\eta (t^*_-(\eta ))-S_\eta (t)} \end{aligned}$$

where the square root is defined so that $\mathfrak {R}v_\eta (t)>0$ along the path $\gamma _\eta$ lying between $t^*_-(\eta )$ and 1. The function $v_\eta (t)$ will be an essential change of variables in the integral (139) so we mention its important properties.

#### Lemma 6

If $M>0$ is sufficiently large, the function $v_\eta (t)$ for every $\eta \in \varOmega _+$ is:( 1) biholomorphic in $t\in B(1/2,2r)$; (2) satisfies the condition

276 $$\begin{aligned} \left| v_\eta (t_1)-v_\eta (t_2)\right| = (2+O(M^{-1}))\left| t_1-t_2\right| \end{aligned}$$

for any $t_1,t_2\in B(1/2,2r)$. Moreover, if $I_\eta$ is the image of *B*(1/2, 2*r*) under the map $v_\eta (t)$, then there exists a $\delta _*>0$ such that $\displaystyle {{B(0,\delta _*)\subset \bigcap \nolimits _{\eta \in \varOmega _+}I_\eta }}$.

#### Proof

Statement (1) follows directly from

277 $$\begin{aligned} v_\eta (t)=\left( t-t^*_-(\eta )\right) \sqrt{-\frac{1}{2}S_\eta ''(t^*_-(\eta ))+{\text {O}}\left( t-t^*_-(\eta )\right) }, ~~ t\in B(1/2,2r). \end{aligned}$$

To prove statement (2), we observe that Proposition 5 implies $S_\eta ''(t^*_-(\eta ))=8i+O(M^{-1})$. Then, taking into account (277),

278 $$\begin{aligned} v'_\eta (t)=\frac{-S_\eta '(t)}{2v_\eta (t)}=\sqrt{-\frac{1}{2}S_\eta ''(t^*_-(\eta ))}+O(M^{-1}), \end{aligned}$$

in *B*(1/2, 2*r*), so that $|v'_\eta (t)|=2+O(M^{-1})$ there. That implies (276). To prove the last statement, define the function

279 $$\begin{aligned} \delta _*(\eta )=\min \{v\, : ~ v\in \partial I_\eta \} \end{aligned}$$

for all $\eta \in \varOmega _+$. Since $\delta _*(\eta )$ is a continuous positive function on the compact boundary $\partial \varOmega _+$, it must attain its minimal value $\delta _*>0$. $\square$

Let $\delta =\frac{\delta _*}{2}$ and define

280 $$\begin{aligned} t^*_L(\eta ):=v^{-1}_\eta (-\delta ), ~~~ t^*_R(\eta ):=v^{-1}_\eta (\delta ). \end{aligned}$$

With the previous Lemma in mind, we now write ${\gamma _\eta =\gamma _{L,\eta }+\gamma _{C,\eta }+\gamma _{R,\eta }}$, where $\gamma _{C,\eta }$ is the image of $[-\delta ,\delta ]$ under the map $v^{-1}_\eta$, $\gamma _{L,\eta }$ is the portion of $\gamma$ beginning at $t=0$ and ending at $t=t^*_L(\eta )$, and $\gamma _{L,\eta }$ is the portion of $\gamma$ beginning at $t=t^*_R(\eta )$ and ending at $t=1$. The curves $\gamma _{L,\eta },\gamma _{C,\eta },\gamma _{R,\eta }$ are pictured in Fig. 7.

#### Remark 17

Notice that

281 $$\begin{aligned} \left| t_-^*(\eta )-t_R^*(\eta )\right| =\left| v_\eta ^{-1}(0)-v_\eta ^{-1}(\delta )\right| \ge \frac{2}{9}|\delta -0|>0 \end{aligned}$$

for all $\eta \in \varOmega _+$ by use of (276). An identical statement holds for $t_L^*(\eta )$.

#### Lemma 7

Choose sufficiently large *M*. Then, under the hypotheses of Theorem 14

282 $$\begin{aligned}&\int _{\gamma _{C,\eta }}F(t,\eta ,\lambda )e^{-\mathfrak {I}[a(\lambda )]S_\eta (t)}dt \nonumber \\&\quad =e^{-\mathfrak {I}[a(\lambda )]S_\eta (t^*_-(\eta ))}F\left( t^*_-(\eta ),\eta ,\lambda \right) \sqrt{\frac{2\pi }{\mathfrak {I}[a(\lambda )]S''_\eta (t^*_-(\eta ))}} \left[ 1+{\text {O}}\left( \frac{M^2}{\mathfrak {I}[a(\lambda )]}\right) \right] \end{aligned}$$

as $\lambda \rightarrow 0$, $\mathfrak {I}\lambda \ge 0$, uniformly in $\eta \in \varOmega _+$.

#### Proof

By definition, $\gamma _{C,\eta }$ is the path of steepest descent for $S_\eta (t)-S_\eta (t^*_-(\eta ))$ and thus $\mathfrak {I}[S_\eta (t)-S_\eta (t^*_-(\eta ))]=0$ and $\mathfrak {R}[S_\eta (t)-S_\eta (t^*_-(\eta ))]\le 0$ on $\gamma _{C,\eta }$ with equality only when $t=t_-^*(\eta )$. Using change of variables

283 $$\begin{aligned} v=v_\eta (t)=\sqrt{S_\eta (t^*_-(\eta ))-S_\eta (t)}, \end{aligned}$$

we obtain

284 $$\begin{aligned} \int _{\gamma _{C,\eta }}F(t,\eta ,\lambda )e^{-\mathfrak {I}[a]S_\eta (t)}dt&=e^{-\mathfrak {I}[a]S_\eta (t^*_-)}\int _{\gamma _{C,\eta }}F(t,\eta ,\lambda )e^{-\mathfrak {I}[a][S_\eta (t)-S_\eta (t^*_-)]}dt \nonumber \\&=e^{-\mathfrak {I}[a]S_\eta (t^*_-)}\int _{-\delta }^\delta e^{\mathfrak {I}[a]v^2}F(t(v),\eta ,\lambda )\frac{dv}{v'_\eta (t(v))}. \end{aligned}$$

Since $v_\eta (t)$ is biholomorphic in *B*(1/2, 2*r*) for every $\eta \in \varOmega _+$, both $\frac{dt}{dv}$ and $F(t(v),\eta ,\lambda )$ are analytic for $v\in B(0,\delta _*)$ by Lemma 6. So

285 $$\begin{aligned} \frac{F(t(v),\eta ,\lambda )}{v'_\eta (t(v))}=\sum _{n=0}^\infty b_{n}(\eta ,\lambda )v^{n}=:b_0(\eta ,\lambda )+vb_1(\eta ,\lambda )+v^2\hat{R}_2(v,\eta ,\lambda ) \end{aligned}$$

where

286 $$\begin{aligned} b_0(\eta ,\lambda )&=F(t^*_-(\eta ),\eta ,\lambda )\sqrt{\frac{2}{-S_\eta ''(t^*_-(\eta ))}}, ~~~ b_{n}(\eta ,\lambda )=\frac{1}{2\pi i}\int _{|w|=\delta }\frac{F(t(w),\eta ,\lambda )}{v'_\eta (t(w)) w^{n+1}}dw \end{aligned}$$

for $n\ge 1$, where, by construction, $\delta <\delta _*$. We have

287 $$\begin{aligned}&e^{-\mathfrak {I}[a]S_\eta (t^*_-)}\int _{-\delta }^\delta e^{\mathfrak {I}[a]v^2}F(t(v),\eta ,\lambda ) \frac{dv}{v'_\eta (t(v))}\nonumber \\&\quad =2e^{-\mathfrak {I}[a]S_\eta (t^*_-)}\int _0^\delta e^{\mathfrak {I}[a]v^2}\left[ b_0(\eta ,\lambda )+v^2 R_2(v,\eta ,\lambda )\right] dv, \end{aligned}$$

where $R_2$ is the even part of $\hat{R}_2$. By the assumptions of the Lemma,

288 $$\begin{aligned} K:=\max _{(\eta ,\lambda )\in \varOmega _+\times B^0(0,\delta _*)}\max _{v\in \partial {B}(0,\delta _*)}\left| \frac{F(t(v),\eta ,\lambda )}{v'_\eta (t(v))}\right| . \end{aligned}$$

*K* is finite. By Cauchy’s estimate we now have

289 $$\begin{aligned} \left| b_{2n}(\eta ,\lambda )\right| \le \frac{K}{\delta _*^{2n}} \end{aligned}$$

so that for $v\in [0,\delta ]$,

290 $$\begin{aligned} \left| R_2(v,\eta ,\lambda )\right| =\left| \sum _{n=1}^\infty b_{2n}(\eta ,\lambda )v^{2(n-1)}\right| \le \frac{K}{\delta _*^{2}}\sum _{n=1}^\infty \left( \frac{\delta }{\delta _*}\right) ^{2(n-1)}\le \frac{K}{\delta _*^2-\delta ^2}=:R. \end{aligned}$$

Note that $R=O(M^2)$. We fix a sufficiently large *M*. Using a second change of variable $-\tau =\mathfrak {I}[a]v^2$, we rewrite (287) as

291 $$\begin{aligned}&e^{-\mathfrak {I}[a]S_\eta (t^*_-)}\int _{-\delta }^\delta e^{\mathfrak {I}[a]v^2}F(t(v),\eta ,\lambda ) \frac{dv}{v'_\eta (t(v))}= \nonumber \\&\frac{e^{-\mathfrak {I}[a]S_\eta (t^*_-)}}{\sqrt{-\mathfrak {I}[a]}}\int _0^{-\mathfrak {I}[a]\delta ^2}e^{-\tau }\left[ \frac{b_0(\eta ,\lambda )}{\sqrt{\tau }}-\frac{\sqrt{\tau }}{\mathfrak {I}[a]}R_2\left( t\left( \sqrt{\frac{-\tau }{\mathfrak {I}[a]}}\right) ,\eta ,\lambda \right) \right] d\tau \end{aligned}$$

Using now the asymptotics of the Incomplete Gamma function (see [24] 8.2.2, 8.2.11), we have

292 $$\begin{aligned} \int _0^{-\mathfrak {I}[a]\delta ^2}e^{-\tau }\tau ^{1/2-1}d\tau =\varGamma (1/2)-\varGamma (1/2,-\mathfrak {I}[a]\delta ^2)=\sqrt{\pi }+{\text {O}}\left( \sqrt{-\mathfrak {I}[a]}e^{\mathfrak {I}[a]\delta ^2}\right) \end{aligned}$$

as $$\lambda \rightarrow 0$$ and

293 $$\begin{aligned}&\left| \int _0^{-\mathfrak {I}[a]\delta ^2}e^{-\tau }\tau ^{3/2-1}R_2\left( t\left( \sqrt{\frac{-\tau }{\mathfrak {I}[a]}}\right) ,\eta ,\lambda \right) d\tau \right| \nonumber \\&\quad \le R\int _0^{-\mathfrak {I}[a]\delta ^2}e^{-\tau }\tau ^{3/2-1}d\tau =R\left[ \varGamma (3/2)+{\text {O}}\left( \left( -\mathfrak {I}[a]\right) ^{3/2}e^{\mathfrak {I}[a]\delta ^2}\right) \right] \end{aligned}$$

with the error term being uniform with respect to $\eta ,\lambda$. Now we have

294 $$\begin{aligned} \int _{\gamma _{C,\eta }}F(t,\eta ,\lambda )e^{-\mathfrak {I}[a(\lambda )]S_\eta (t)}dt=\frac{e^{-\mathfrak {I}[a]S_\eta (t^*_-)}}{\sqrt{-\mathfrak {I}[a]}}\left[ b_0(\eta ,\lambda )\sqrt{\pi }+{\text {O}}\left( \frac{M^2}{\mathfrak {I}[a]}\right) \right] , \end{aligned}$$

which is equivalent to the statement of the Lemma since, by the hypotheses of the Lemma, $b_0(\eta ,\lambda )$ is uniformly bounded away from 0. $\square$

Next we show that the contribution away from the saddle point is negligible.

#### Lemma 8

Assume $F(t,\eta ,\lambda )$ satisfies the hypotheses of Theorem 14. Then, as $\lambda \rightarrow 0$ with $\mathfrak {I}\lambda \ge 0$, we have the bound

295 $$\begin{aligned} \left| \int _{\gamma _{L+R,\eta }}F(t,\eta ,\lambda )e^{-\mathfrak {I}[a(\lambda )]S_\eta (t)}~dt\right| \le e^{-\mathfrak {I}[a(\lambda )]\mathfrak {R}[S_\eta (t^*_-(\eta ))]}\cdot e^{-\mathfrak {I}[a(\lambda )]c_*} K_{LR}, \end{aligned}$$

where $$\gamma _{L+R,\eta }=\gamma _{L,\eta }\cup \gamma _{R,\eta }$$ and constants $$c_*<0$$ and $$ K_{LR}>0$$ are $$\eta ,\lambda $$ independent.

#### Proof

Define

296 $$\begin{aligned} c_*(\eta )=\max _{t\in \gamma _{L+R,\eta }}\mathfrak {R}\left[ S_\eta (t)-S_\eta (t_-^*(\eta ))\right] . \end{aligned}$$

Obviously $c_*(\eta )$ is a continuous and negative function of $\eta \in \varOmega _+$. Since $\varOmega _+$ is a compact set, the maximal value $c_*$ of $c_*(\eta )$ on $\varOmega _+$ is negative. Then

297 $$\begin{aligned}&\left| \int _{\gamma _{L+R,\eta }}F(t,\eta ,\lambda )e^{-\mathfrak {I}[a(\lambda )]S_\eta (t)}~dt\right| \nonumber \\&\quad \le e^{-\mathfrak {I}[a(\lambda )]\mathfrak {R}[S_\eta (t^*_-(\eta ))]}\int _{\gamma _{L+R,\eta }}\left| F(t,\eta ,\lambda )\right| e^{-\mathfrak {I}[a(\lambda )]\mathfrak {R}\left[ S_\eta (t)-S_\eta (t^*_-(\eta ))\right] }~dt \nonumber \\&\quad \le e^{-\mathfrak {I}[a(\lambda )]\mathfrak {R}[S_\eta (t^*_-(\eta ))]}e^{-\mathfrak {I}[a(\lambda )]c_*}\int _{\gamma _{L+R,\eta }}\left| F(t,\eta ,\lambda )\right| ~dt\nonumber \\&\quad \le e^{-\mathfrak {I}[a(\lambda )]\mathfrak {R}[S_\eta (t^*_-(\eta ))]}\cdot e^{-\mathfrak {I}[a(\lambda )]c_*} K_{LR} \end{aligned}$$

where the constant

298 $$\begin{aligned} K_{LR}:=\max _{\eta \in \varOmega _+}\max _{\lambda \in \overline{B}(0,\epsilon )}\int _{\gamma _{L+R,\eta }}\left| F(t,\eta ,\lambda )\right| ~dt. \end{aligned}$$

is finite by hypotheses of Theorem 14. $\square$

The combination of Lemmas 7 and 8 proves Theorem 14 provided that $\lambda$ is in the upper half plane.

#### Remark 18

When $\lambda \rightarrow 0$ in the lower half plane, the key difference is that $\mathfrak {I}[a(\lambda )]\rightarrow \infty$ (see Proposition 7). With that in mind, rewrite the integrand of (139) as

299 $$\begin{aligned} F(t,\eta ,\lambda )e^{-\mathfrak {I}[a(\lambda )]S_\eta (t)}=F(t,\eta ,\lambda )e^{\mathfrak {I}[a(\lambda )][-S_\eta (t)]}. \end{aligned}$$

We can replace *S* with $-S$ and carry out the same analysis as before. The only difference will be that the regions in the *t* plane where $\mathfrak {R}[S_\eta (t)-S_\eta (t^*_-(\eta ))]<0$ and $\mathfrak {R}[S_\eta (t)-S_\eta (t^*_-(\eta ))]>0$ will swap, so we deform [0, 1] to a different contour, see Fig. 8. All previous ideas from this section can now be applied using the new contour $\gamma$.
